# Dynamic patterns of repeats and retrotransposons in the centromeres of *Humulus lupulus* L.

**DOI:** 10.1111/nph.70380

**Published:** 2025-07-15

**Authors:** Lucie Horáková, Pavel Jedlička, Radim Čegan, Pavla Navrátilová, Hiroyuki Tanaka, Atsushi Toyoda, Takehiko Itoh, Takashi Akagi, Eiichiro Ono, Vojtěch Hudzieczek, Josef Patzak, Jan Šafář, Roman Hobza, Václav Bačovský

**Affiliations:** ^1^ Department of Plant Developmental Genetics Institute of Biophysics of the Czech Academy of Sciences 61200 Brno Czech Republic; ^2^ Department of Experimental Biology, Faculty of Science Masaryk University 62500 Brno Czech Republic; ^3^ Institute of Experimental Botany of the Czech Academy of Sciences Centre of Plant Structural and Functional Genomics 77900 Olomouc Czech Republic; ^4^ School of Life Science and Technology Institute of Science Tokyo Tokyo 152‐8550 Japan; ^5^ Advanced Genomics Center National Institute of Genetics Mishima 411‐8540 Shizuoka Japan; ^6^ Graduate School of Environmental and Life Science Okayama University 1‐1 Tsushima‐naka, Kita‐ku Okayama 700‐8530 Japan; ^7^ Research Institute, Suntory Global Innovation Center Ltd 8‐1‐1 Seikadai, Seika, Soraku Kyoto 619‐0284 Japan; ^8^ Hop Research Institute Co. Ltd 43846 Žatec Czech Republic

**Keywords:** asymmetric cell division, Cannabaceae, centromere, retrotransposons, sex chromosomes

## Abstract

- The centromere has a conserved function across eukaryotes; however, the associated DNA sequences exhibit remarkable diversity in both size and structure. In plants, some species possess well‐defined centromeres dominated by tandem satellite repeats and centromeric retrotransposons, while others have centromeric regions composed almost entirely of retrotransposons.
- Using a combination of bioinformatic, molecular, and cytogenetic approaches, we analyzed the centromeric landscape of *Humulus lupulus*. We identified novel centromeric repeats and characterized two types of centromeric organization. Cytogenetic localization on metaphase chromosomes confirmed the genomic distribution of the major repeats and revealed unique centromeric organization specifically on chromosomes 2, 8, and Y.
- Two centromeric types are composed of the major repeats SaazCEN and SaazCRM1 (Ty3/*Gypsy*) which are further accompanied by chromosome‐specific centromeric satellites, Saaz40, Saaz293, Saaz85, and HuluTR120. Chromosome 2 displays unbalanced segregation during mitosis and meiosis, implicating an important role for its centromere structure in segregation patterns. Moreover, chromosome 2‐specific centromeric repeat Saaz293 is a new marker for studying aneuploidy in hops.
- Our findings provide new insights into chromosome segregation in hops and highlight the diversity and complexity of the centromere organization in *H. lupulus*.

## Introduction

Centromeres are the sites of kinetochore assembly, ensuring faithful segregation of chromosomes to daughter cells during mitosis and meiosis. Active regional centromeres are defined by the presence of the centromere‐specific histone variant H3 (termed as CENH3 in plants). The CENH3‐positive chromatin domain is localized within the primary constriction of monocentric chromosomes, allowing precise centromere localization in association with centromeric DNA (Houben *et al*., 2007; Mendiburo *et al*., 2011; Gent *et al*., 2017; Maheshwari *et al*., 2017). Despite the evolutionary conservation of centromere function across eukaryotes (Henikoff *et al*., 2001), the amino acid sequence of CENH3, the size of centromeres, and the identity of centromeric repeat sequences exhibit remarkable divergence among closely related species (Melters *et al*., 2013; Maheshwari *et al*., 2017). In plants, centromeric DNA is usually composed of tandem repeats, organized into higher‐order repeat structures, along with centrophilic long terminal repeat (LTR) retrotransposons, mostly belonging to the Ty3/*Gypsy* superfamily (reviewed in Naish & Henderson, 2024). Retrotransposon‐based centromeres have been identified among other in barley (Presting *et al*., 1998), einkorn wheat (Ahmed *et al*., 2023), apple (Zhang *et al*., 2019), the moss *Physcomitrella patens* (Bi *et al*., 2024), highlighting rapid centromere evolution and diversity across the plant kingdom (Lee *et al*., 2005). Centromeres are further embedded in pericentromeric chromatin, typically defined by histone modifications such as H3S10ph or H2At120ph, which contribute to the formation of a repressive chromatin state and have a crucial role in kinetochore assembly (Neumann *et al*., 2016).

The role of (peri)centromeric chromatin and the kinetochore complex in atypical chromosome behavior has been described in interspecific plant hybrids of *H. vulgare* × *H. bulbosum* (Sanei *et al*., 2011) and *Festuca* × *Lolium* (Majka *et al*., 2023). In the latter study, both parental sexes were shown to influence non‐Mendelian inheritance, illustrating the role of centromere structure and function in chromosome elimination and genome dominance. In addition to the essential centromere function, centromeres can experience a phenomenon known as centromere shift, where the original centromere relocates to a new position. Recent studies on einkorn wheat have shown that its centromeres are composed almost entirely of two major retrotransposons, *RLG_Cereba* and *RLG_Quinta*, which exhibit recent insertions into einkorn's functional centromeres (Ahmed *et al*., 2023; Heuberger *et al*., 2024). At least four chromosomes in the einkorn genome have been found to undergo structural chromosome rearrangements, where existing centromeres have shifted to a new position (Ahmed *et al*., 2023; Heuberger *et al*., 2024). Besides, another important evolutionary mechanism is centromere repositioning. This involves the formation of *de novo* centromeres in different positions on the same chromosome, accompanied by the inactivation of the original centromere. This phenomenon has been identified in various plant species, such as Arabideae (Mandáková *et al*., 2020), soybean (Liu *et al*., 2023), maize (Schneider *et al*., 2016), cucurbit species (Han *et al*., 2009) and wheat (Zhao *et al*., 2023), among others. On a broader scale, centromere repositioning has been shown to modify recombination frequency and gene expression, driving karyotype diversity in plants (Mandáková *et al*., 2020).

*Humulus lupulus* L. (common hop) is a perennial dioecious plant with heteromorphic sex chromosomes (20, XX in females and 20, XY in males; Winge, 1923). Its genome is relatively large (2.8 Gb), with a considerable amount of repetitive DNA (64%) and a high level of heterozygosity (Natsume *et al*., 2015; Padgitt‐Cobb *et al*., 2023). Female inflorescences, known as hop cones, contain important secondary metabolites (bitter acids, polyphenols and terpenes) with antimicrobial properties, making hops a necessary commodity used in the brewing and pharmaceutical industries (Neve, 1991; Zanoli & Zavatti, 2008). The genus *Humulus* includes *Humulus japonicus* Siebold & Zuccarini (Japanese hop) and five *Humulus* varieties: *H. lupulus* var. *lupulus*, *H. lupulus* var. *lupuloides*, *H. lupulus* var. *neomexicanus*, *H. lupulus* var. *pubescens*, and *H. lupulus* var. *cordifolius* (Small, 1978). This diversity makes this genus a valuable model for studying sex chromosome evolution and intraspecific sex chromosome genome dynamics.

Previous genetic studies of *H. lupulus* have shown that a significant proportion of molecular markers deviate from expected Mendelian segregation ratios (Seefelder *et al*., 2000; McAdam *et al*., 2013; Zhang *et al*., 2017). Cytogenetic evidence has revealed the presence of multivalent chromosome complexes and unusual chromosome morphology during prophase I that lead to the formation of anaphase bridges during male hop meiosis (Sinotô, 1929; Ono, 1955; Neve, 1958; Haunold, 1974; Zhang *et al*., 2017; Easterling *et al*., 2018, 2020). These abnormalities were demonstrated further as non‐Mendelian segregation patterns, increasing genetic diversity and genome size. However, such instabilities pose significant challenges for the breeding of new cultivars, compounded by the absence of accessible tools to identify chromosome instabilities.

Despite the availability of genomic resources and cytogenetic data on chromosome irregularities, the mechanism underlying non‐Mendelian segregation in *H. lupulus* is still under debate. Earlier studies hypothesized that this phenomenon could be a conserved genomic feature of *H. lupulus*, a consequence of breeding, or associated with an unusual centromere structure, even involving the presence of a dicentric chromosome. Thus, understanding centromere function in *H. lupulus* will be crucial for the selection and identification of stable parental lines, enhancing hop breeding programs and marker‐assisted selection. Beyond its practical applications, the study of centromere structure in *H. lupulus* may provide insights into genomic changes during the evolution of the Cannabaceae family.

To clarify whether the centromere organization influences aberrant segregation of chromosomes in *H. lupulus* var. *lupulus*, we developed a *H. lupulus* CENH3 antibody and analyzed the centromeric landscape of autosomes and sex chromosomes. Utilizing ChIP‐seq data on the female and male *H. lupulus* genomes, combined with detailed karyotypic analysis, we identified novel centromeric repeats within centromeric subdomains and estimated the recent insertion of a CRM retrotransposons belonging to the Ty3/*Gypsy* family, which is associated with CENH3 domains. In addition, we characterized an autosomal pair involved in aberrant segregation, leading to the formation of inviable microspores, micronuclei, and reduced fertility. We propose a possible mechanism that would explain atypical segregation patterns and processes affecting the mis‐segregating autosomes. These new findings contribute to a deeper understanding of the complexity of the hop genome and the behavior of chromosomes during cell division.

## Materials and Methods

### Plant material

Female *Humulus lupulus* L. var. *lupulus* cv Saaz – Osvald's clone 72 (2*n* = 20, XX), male named ‘Liběšice’ (Lib male), and other three male accessions (15 246, 15 249, 15 276, wild hop II., dwarf hop; 2*n* = 20, XY) used in this study (Supporting Information Table S1) were provided by the Hop Research Institute Co. Ltd in Žatec (Czech Republic). All plants were grown in a glasshouse under 16 h : 8 h, daylight : dark conditions at the Department of Plant Developmental Genetics in Brno (Czech Republic).

### Generation of *H. lupulus* CENH3 antibody

The gene coding for the CENH3 protein was identified in HopBase (Hill *et al*., 2017) and the NCBI database using the tblastn algorithm. The resulting sequence was compared to the published CENH3 library (Fig. S1). HlCENH3 primers were designed in Geneious Prime (2023.1.1) and synthesized in GeneriBiotech (Table S2). The HlCENH3 gene was amplified using Q5 High‐fidelity DNA polymerase (M0491S; NEB, Ipswich, MA, USA), following the manufacturer's instructions. Resultant PCR products were purified using the QIAquick PCR Purification Kit (28104; Qiagen) and used for blunt cloning using the CloneJET PCR Cloning Kit (K1231; ThermoFisher, Brno, Czech Republic). Sequencing primers (pJET1.2F/R) were used in all reactions. Samples were sequenced by Macrogen (Amsterdam, the Netherlands). Multiple sequence alignments and sequence conservation were verified using Geneious Prime (Fig. S2). The peptide for antibody synthesis was selected based on its hydrophilic profile determined using the Kyte‐Doolittle algorithm with linear weight variation model (Kyte & Doolittle, 1982). The histone core domains were determined in HistoneDB 2.0 (Draizen *et al*., 2016). The antibody against the CENH3 protein of *H. lupulus* was custom raised using the peptide N‐SPATTPKKAARTK‐C. Peptide synthesis, immunization of rabbit to produce anti‐HlCENH3 antibody, and final peptide affinity purification of antisera were performed by Genescript (USA).

### Indirect immunostaining

Interphase nuclei of *H. lupulus* Saaz female were prepared as described in Houben *et al*. (2003) and Bačovský *et al*. (2019) with some modifications. After the fixation of roots in 4% paraformaldehyde and 1× PBS, roots were incubated in 2% PVP‐40 (polyvinylpyrrolidone) and 1% Triton X‐100 dissolved in 1× PBS for 15 min on ice (Lunerová & Vozárová, 2023). For immunostaining, slides were incubated overnight at 4°C with HlCENH3 antibody (diluted 1 : 1000). After washing the primary antibody, anti‐rabbit secondary antibody (FITC‐conjugated, diluted 1 : 200) in 1% blocking solution was used for 60 min at 37°C. Slides were washed in 1× PBS, dehydrated in an ethanol series, and mounted in Vectashield Antifade Mounting Medium supplemented with 4′,6‐diamidino‐2‐phenylindole (DAPI). Images were captured using an Olympus AX70 epifluorescence microscope equipped with a cooled cube camera and processed using Adobe Photoshop. Immunostaining was performed in two independent experiments.

### Chromatin immunoprecipitation with sequencing (ChIP‐seq)

Nuclei for ChIP experiments were isolated from young leaves of Lib male *H. lupulus*. The ChIP‐seq protocol with anti‐HlCENH3 antibody was performed as described in Navrátilová *et al*. (2022) in two biological replicates. The sequencing of the libraries was conducted using an Illumina NovaSeq instrument with 150 bp paired‐end reads and a NovaSeq S1 flowcell (Illumina Inc., San Diego, CA, USA) at the Centre of Plant Structural and Functional Genomics in Olomouc, Czech Republic.

### Analysis of ChIP‐seq data

The reads of HlCENH3‐ChIP and input control were quality checked and filtered as genomic DNA using FastQC and Trimmomatic 0.32 (Bolger *et al*., 2014). To identify centromeric candidates and evaluate the enrichment of repetitive sequences in sequencing data from the HlCENH3‐ChIP experiment, we applied the approach based on RepeatExplorer2 (Novák *et al*., 2013, 2020) and the ChIP‐seq Mapper tool (Neumann *et al*., 2012) similarly to Navrátilová *et al*. (2022), with the repeat contig sequences of *H. lupulus* identified by Repeatexplorer2 as a reference. For further analyses, we used either the consensus sequence of the candidate cluster (if available) or the individual contigs from the candidate cluster identified by blastn search (if the Repeatexplorer2 output did not show the consensus sequence of the cluster). In parallel, the trimmed reads of input and HlCENH3‐ChIP datasets were used for centromere identification and characterization in the *Humulus* genomes. Briefly, reads were mapped to Saaz female and male ‘10–12’ published in Akagi *et al*. (2025) using Bwa‐Mem2 (Vasimuddin *et al*., 2019) and subsequently analyzed by MACS3 (Zhang *et al*., 2008). The most enriched regions on each chromosome were considered as the center of the centromeric region and from this site we extracted 3 Mbp upstream and downstream (in total 6 Mbp) for detailed analysis. Both *H. lupulus* genomes were assembled using PacBio HiFi and Illumina Hi‐C reads. The assembly size for Saaz is 5.24 Gb in diploid genome, with the N50 of 248.86 Mb, and 4.90 Gb for ‘10–12’, with a N50 of 250.50 Mb. Busco analysis confirms high completeness of genome assembly for both accessions, with the 98.24% and 98.28% in Saaz and ‘10–12’, respectively. We identified no gaps in the selected 6 Mbp and neighboring regions in either the male or female. Both genomes are, therefore, reliable for the centromere annotation and repeat diversity analysis (Akagi *et al*., 2025).

### Repeat annotation in the centromeric regions of *Humulus* species

We used genome assembly of female and male *H. lupulus* var. *lupulus* (Akagi *et al*., 2025), *H. lupulus* var. *lupulus* cv Cascade (Padgitt‐Cobb *et al*., 2023; GCA_023660075.1) and European hops (SAMEA7522047; GCA_963169125.1) generated by the Darwin Tree of Life Project for mapping of RepeatExplorer generated repeat clusters using Repeatmasker (v.4.1.1; Tarailo‐Graovac & Chen, 2009). For identification of intact LTR retrotransposons insertions were used Domain‐based Annotation of Transposable Elements (DANTE) for long terminal repeat (DANTE_LTR; Novák *et al*., 2024). The insertion age was estimated using Long Tandem Repeats divergence (Jedlicka *et al*., 2020) and synonymous substitution rate 6.1 × 10^−9^ (Padgitt‐Cobb *et al*., 2023). In order to distinguish possible lineages of autonomous and nonautonomous CRM Ty3/*Gypsy* elements, all intact CMR transposons were aligned using Mafft v.7 (Katoh & Standley, 2013) and a Maximal‐Likelihood tree was generated using Fasttree 2 (Price *et al*., 2010). To determine which region of SaazCEN predominates in CRM LTRs, all repeat fragments present in CRM LTRs were remapped using the Bwa aligner (Li, 2013).

### Preparation of DNA probes

The total genomic DNA of *H. lupulus* was isolated from young leaves using a NucleoSpin Plant II kit (740770‐50; Macherey‐Nagel GmbH and Co. KG., Düren, Germany), according to the manufacturer's instructions. The primers for centromeric repeats were designed using Geneious Prime (2023.1.1) based on the RepeatExplorer2 consensus sequences. The primer sequences are listed in Table S2. DNA probes were amplified using PCR in a mixture containing 1× PCR buffer, 0.0001 M dNTPs, 0.0001 M of each primer, 0.5 U Taq polymerase (Top Bio, Vestec, Czech Republic), 10–15 ng of template DNA in a total of 20 μl. PCR cycling conditions were according to the manufacturer's instructions (95°C for 4 min followed by 35 cycles of 94°C for 30 s, 55–60°C for 35 s, 72°C for 30 s and final extension step at 72°C for 10 min). The annealing temperature was optimized for each primer pair. PCR products were separated on a 1% agarose gel with EtBr staining and purified using the QIAquick PCR Purification Kit (28104; Qiagen). Purified DNA (1 μg) was labeled with Atto488 NT (PP‐305L‐488), Atto550 NT (PP‐305L‐550) or Cy5 (PP‐305L‐647N) using Nick translation labeling kits (Jena Bioscience, Jena, Germany) following the manufacturer's instructions. After 90 min at 15°C, the NICK translation products were analyzed using a 1% agarose gel with EtBr staining. The reaction of well‐labeled DNA probes was stopped with the addition of 0.5 M EDTA and incubation at 85°C. The labelled probes were then used directly in the hybridization mixture.

### Mitotic and meiotic chromosome preparation

Mitotic chromosomes were prepared as described in Divashuk *et al*. (2014) with some modifications. Young leaves (2–5 mm in length) were collected from intensively grown female and male plants of *H. lupulus*. The leaves were pretreated with 0.002M 8‐hydroxyquinoline for 4 h (2 h at RT and 2 h at 4°C in the dark) and then fixed in Clarke's fixative (ethanol : glacial acetic acid, 3 : 1, v/v) at 4°C overnight. The fixative was replaced with 70% ethanol and the leaves were used directly for squashing (or stored at −20°C until use). Fixed leaves were washed 1× 5 min in distilled water, 1× 5 min in 45% acetic acid, 2× 5 min in 0.001 M citrate buffer and macerated in 1% enzyme mixture (Table S3) diluted in 0.001 M citrate buffer for 50 min at 37°C. Leaves were then squashed in 60% acetic acid. After freezing in liquid nitrogen, the coverslip was removed, and slides were incubated in freshly prepared Clarke's fixative for 3–5 min. Prepared slides with well‐preserved metaphase were used for FISH or stored at −20°C in 96% ethanol until use.

Male panicles were fixed in Clarke's fixative for 24 h at room temperature, the fixative was replaced, and material was prepared using the squashing method above or stored at −20°C until used. Single anthers were washed 2× 5 min in 0.001 M citrate buffer and macerated in 1% enzyme mixture (Table S3) diluted in 0.001 M citrate buffer for 30 min at 37°C. Slides with meiotic chromosomes were prepared using the same procedure as for mitotic chromosomes, as described above.

### Fluorescence *in situ* hybridization (FISH)

FISH was performed on mitotic and meiotic chromosomes as described in Sacchi *et al*. (2024). The hybridization mixture (stringency 77%) contained 50% formamide, 10% dextran sulfate, 2× SCC, and 1.5 ng μl^−1^ of each probe per slide. Chromosomes were counterstained with DAPI in Vectashield Antifade Mounting Medium. Images were captured using an Olympus AX70 epifluorescence microscope equipped with a CCD camera and processed using Adobe Photoshop. FISH with specific DNA probes was performed in at least three individual experiments, and at least 10 metaphases were analyzed per experiment.

### Pollen grain viability

Male panicles of three plants Lib male, F_1_ progeny (Osvald's clone 72 × Lib male), and wild hops I. grown in Brno‐Horní Heršpice (Table S1) were fixed in Carnoy's fixative (ethanol : chloroform : acetic acid, 6 : 3 : 1, v/v). Pollen tetrads and pollen grains were stained according to (Peterson *et al*., 2010) with minor modifications. Fixed anthers were squeezed in the staining solution. Slides with anthers were heated on a hot plate at 83°C for 10 min. Images of pollen tetrads and pollen grains were captured using an Olympus CX43 microscope. The number of tetrads produced by microsporogenesis and the number of viable (purple) and nonviable (gray) pollen grains were counted in ImageJ Fiji (1.52) using the Multi point Tool function.

## Results

### Analysis of centromeric landscape in *H. lupulus* and position of the pseudoautosomal region (PAR)

**Fig. 1:**
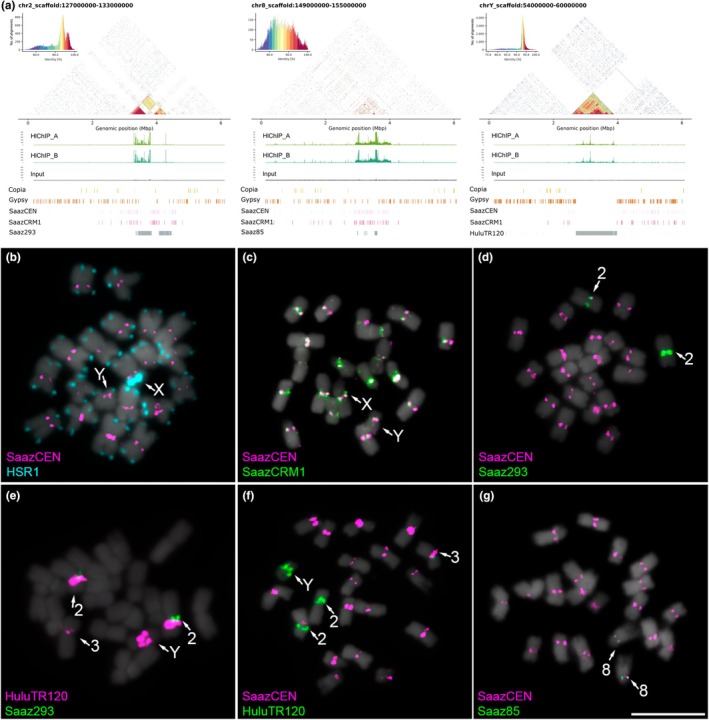
Characterization of the centromeric regions in *Humulus lupulus*. (a) Centromere characterization of chromosomes 2, 8, and X in *H. lupulus*. Heatmaps display pairwise sequence identity with dot plots revealing organization on all three chromosomes colored by percent identity. The first three lines compare HlCENH3 ChIP‐seq data replicates (green and blue) against the input control. The subsequent lines show the detailed distribution of long terminal repeat retrotransposons (Ty1/*Copia*, Ty3/*Gypsy*, and SaazCRM1) and chromosome‐specific centromeric satellites Saaz293 and HuluTR120. Notably, chromosomes 2 and Y show a unique satellite array pattern. (b) Distribution of centromeric and centromere‐related repeats on metaphase chromosomes of male *Humulus lupulus*. The centromeric repeat SaazCEN (magenta) is shown together with the subtelomeric tandem repeat HSR1 (cyan). The HSR1 repeat allows the X and Y chromosomes (arrows) to be distinguished. HSR1 is present in the subtelomeric region of all autosomes, except the p‐arm of chromosome 8, and the X chromosome (HSR1 signal in pericentromeric region). The Y chromosome displays the HSR1 satellite in the subtelomeric region of the p‐arm. (c) Distribution of retrotransposon SaazCRM1 (green) and its colocalization with SaazCEN (magenta) in centromeric regions of all chromosomes. (d) Saaz293 satellite (green) colocalization with SaazCEN (magenta) in the centromeric region of chromosome 2. (e) Saaz293 satellite (green) and HuluTR120 satellite (magenta) are localized together on chromosome 2. HuluTR120 is additionally present in the pericentromeric region of one chromosome 3 and in centromeric and pericentromeric regions of chromosome Y. (f) Localization of the HuluTR120 satellite (green) in the pericentromeric region of chromosome pair 2 and of one chromosome 3, while SaazCEN (magenta) marks the centromeric region. The Y chromosome exhibits both centromeric and pericentromeric signals of HuluTR120. (g) The Saaz85 satellite (green), together with the SaazCEN (magenta), constitutes the centromere of chromosome 8. Mitotic chromosomes were counterstained with 4′,6‐diamidino‐2‐phenylindole (DAPI). Bar, 10 μm.

To investigate the centromeric structure of female and male *H. lupulus*, we developed a *H. lupulus*‐specific antibody targeting the centromeric histone variant of histone H3, designated as HlCENH3 (Figs S1, S2). Immunostaining of HlCENH3 revealed 20 round‐shaped structures in the interphase nucleus, correlating with the number of chromosomes observed in *H. lupulus* Saaz female and Lib male (2*n* = 20; Fig. S3). This supports previous observations of the monocentric organization of *Humulus* chromosomes. The validated functional HlCENH3 antibody was then used for ChIP‐seq analysis. We found monocentric localization of HlCEHN3 enrichment in all 10 chromosome pairs of *H. lupulus* (Figs 1, S4, S5). These CENH3 regions and their surrounding areas resulted in 6 Mb‐long sequences, which we classified as centromeric domains for further detailed analysis. ChIP‐seq data analysis using Repeatexplorer2 (Novák *et al*., 2013, 2020) and ChIP‐seq Mapper tool (Neumann *et al*., 2012) revealed six centromere‐specific repeat candidates – major centromeric sequence SaazCEN (named as Saaz hop centromere), the most abundant centrophilic TEs – SaazCRM1, and the centromeric‐associated satellites – Saaz293, HuluTR120, Saaz85, and Saaz40 (Table 1). The centromeric association of one of these repeats, Saaz293, was also revealed by the analysis of the repetitive DNA fraction in both male and female genomes of *H. lupulus* (Methods S1; Notes S1; Tables S4, S5).

**Table 1:** Centromeric repetitive sequences in the *Humulus lupulus* genome.

Repeats	Monomer length (bp)	ChIP Hits	Input hits	Normalized ratio ChIP/input	Annotation
SaazCEN	284	89 401	6006	31.0	Satellite
Saaz293	323	206 894	6777	63.6	Satellite
Saaz85	320	22 376	137 676	0.34	Satellite
Saaz40	324	42 832	906	98.5	Satellite
HuluTR120 (Cl6)	120	402 834	36 562	23.0	Satellite (MN537570; Easterling *et al*., 2020)
SaazCRM1	Variable^a^	713 546	37 509	39.6	Ty3/*Gypsy*_CRM

^a^ Depends on whether it is an autonomous or nonautonomous CRM.

The centromeric localization of centromere‐specific repeat candidates was confirmed by FISH on metaphase chromosomes in both Saaz female (Fig. S6) and Lib male accessions (Fig. 1), and bioinformatic analysis (Fig. 1a). Two newly identified repeats, designated in this study as SaazCEN and SaazCRM1, were enriched in the centromeric region of all chromosomes, including X and Y chromosomes (Figs 1a–c, S4, S5). An exception to the general pattern is chromosome 2, which showed lower densities for both dominant repeats (Fig. S5). The position of the centromere‐specific repeat SaazCEN suggests that the *H. lupulus* karyotype consists of four metacentric (1, 2, 5, and 7), four submetacentric (3, 4, 9, and X), and three acrocentric (6, 8, and Y) chromosomes (Fig. S7a,b). Chromosome nomenclature corresponds to the pseudomolecules in Akagi *et al*. (2025). The centromere of chromosome 2 is primarily enriched for the Saaz293 satellite (Figs 1a,d, S5). Additionally, the pericentromeric region of this chromosome is composed of HuluTR120 (Fig. 1e,f), a satellite with high sequence similarity to GenBank database accession MN537570 (Easterling *et al*., 2020). Physical localization of HuluTR120 revealed additional enrichment on chromosome 3, but only on one chromosome of the homologous pair (Figs 1e,f, S7b). Comparison of three additional male accessions revealed differences in the distribution of HuluTR120, indicating intraspecies variability (Fig. S8). The Lib (Fig. 1e) and 15246 (Fig. S8a) male accessions display HuluTR120 in the pericentromeric region of only one chromosome 3 in the homologous pair, similar to the Saaz female, suggesting structural chromosome heterozygosity and hybrid origin. By contrast, male accessions 15 249 and 15 276 (Fig. S8b,c) exhibit an even number of HuluTR120 signals on both homologous chromosomes. Furthermore, HuluTR120 shows strong enrichment along the whole (peri)centromeric region of the Y chromosome (Figs 1a,e,f, S7b). This pattern is consistent in all three male accessions 15 246, 15 249, and 15 276 (Fig. S8), confirming an ancestral origin of the HuluTR120 satellite on the Y chromosome. Similarly to chromosome 2, the Y chromosome showed lower densities of SaazCEN and SaazCRM1 repeats (Fig. S5). The centromere of chromosome 8 contains the satellites Saaz85 (Fig. 1g) and Saaz40 (Fig. S9a,b), in addition to SaazCEN and SaazCRM1. This chromosome simultaneously possesses 45S rDNA on the p‐arm in both sexes (Fig. S10a,b). The positions of SaazCEN and SaazCRM1 indicate that the HSR1 locus (previously linked to the PAR) is located on the short arm of the Y chromosome (Fig. 1b,c). This locates the PAR on the p‐arm instead of on the q‐arm. During diakinesis, the X and Y chromosomes are associated in an end‐to‐end bivalent conformation, with a subtelomeric HSR1 probe at the chromosome ends (Fig. S11a) and HuluTR120 on the Y chromosome (Fig. S11b). This bivalent conformation supports PAR localization on the p‐arm of the Y chromosome. No differences were observed in the distribution of all repeats on autosomes between sexes (Figs S6, S12). For clarification, all metaphase figures with HSR1 probe are shown in Fig. S12.

### Two centromere types in *H. lupulus* genome organization

**Fig. 2:**
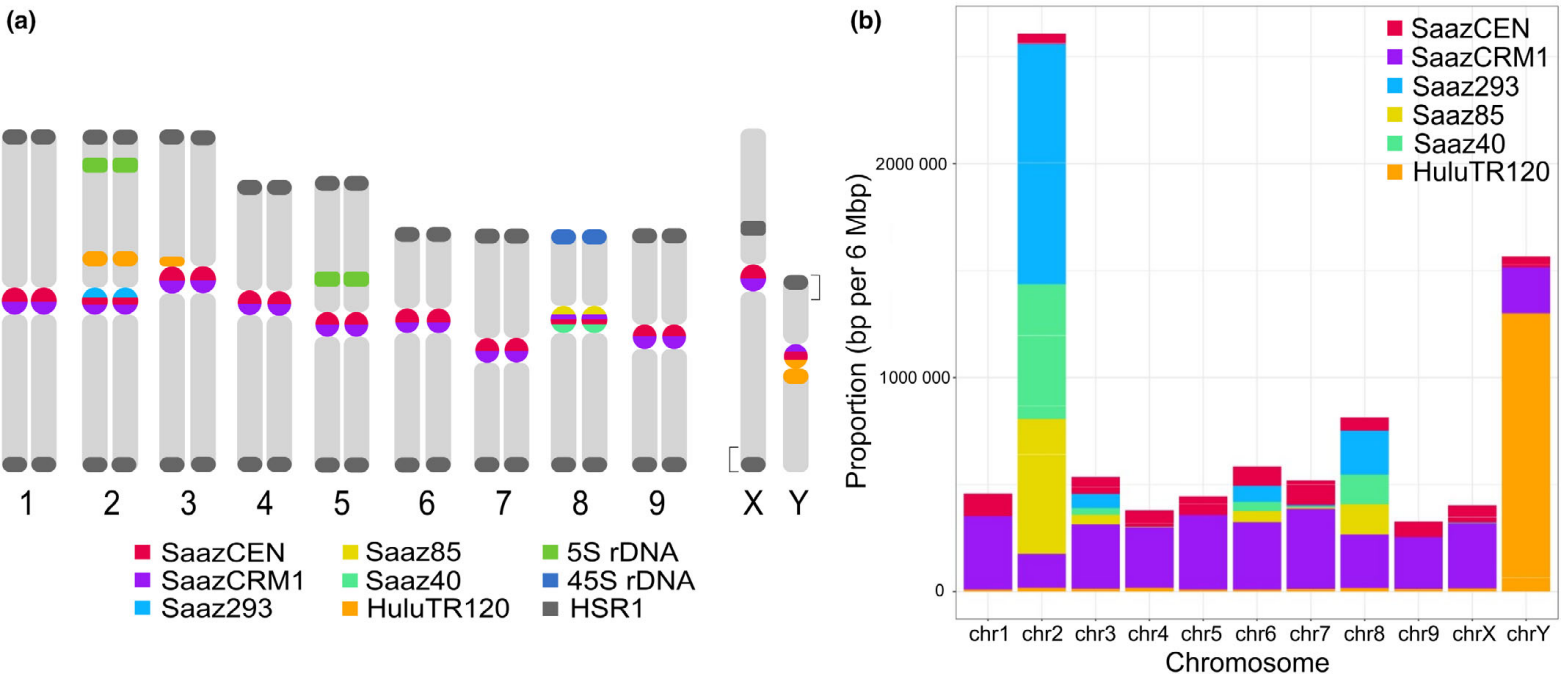
Proportion of identified tandem repeats and long terminal repeat retrotransposon within the centromeres of *Humulus lupulus*. (a) Idiogram of the *H. lupulus* showing FISH localization of centromeric repeats and other *Humulus*‐specific repetitive sequences (Fig. 1, Supporting Information Figs S8–S12). Brackets indicate the positions of pseudoautosomal regions on the sex chromosomes. (b) Proportion of major centromeric repeats (bp per 6 Mbp) for each of the autosomes and sex chromosomes. Centromeres of all chromosomes, including sex chromosomes, are composed of SaazCEN (red) and SaazCRM1 (purple) repeats. Notably, the centromere of chromosomes 2, 8, and Y shows enrichment for large satellite arrays compared to the other autosomes. This is particularly evident in the presence of Saaz293, Saaz40, and Saaz85 on chromosomes 2 and 8, as well as HuluTR120 on the Y chromosome.

The idiogram illustrates the overall distribution of *Humulus* repeats, including HSR1, 5S rDNA, 45S rDNA, and major centromeric repeats newly identified in this work, on the metaphase chromosome of Lib male of *H. lupulus* (Fig. 2a). The proportion of each centromeric repeat in each chromosome, including sex chromosomes, defines two centromere types (Fig. 2b). The first type predominantly contains two major repeats – SaazCEN and SaazCRM1 (Fig. S5). The second type additionally includes Saaz293, Saaz85, and Saaz40 arrays, particularly in chromosomes 2, 3, 6, and 8 (Figs 2b, S5), and the satellite HuluTR120 present specifically on the Y chromosome (Figs 1a, 2b, S7b, S8). However, physical localization defines Saaz293 as a centromere‐specific satellite for chromosome 2 (Fig. 1d), and Saaz40 and Saaz85 as specific for chromosome 8 (Figs 1g, S9). A dot plot diagram of these three satellites revealed an average 50–60% sequence similarity, with conserved and diverged regions along the whole length (Fig. S13). The physical localization and sequence comparison indicated that HuluTR120 is in the pericentromeric regions of chromosomes 2 and 3, and both centromeric and pericentromeric regions of the Y chromosome (Figs 1e,f, 2b, S6). Compared to other autosomes or sex chromosomes (Figs 1a, S4), the strongest enrichment of Saaz293 is within the centromere of chromosome 2 (Figs 1a, 2b, S5). Taken together, our data indicate that chromosomes 2, 8, and Y of *H. lupulus* var. *lupulus* display a unique satellite repeat structure compared to the other autosomes or the X chromosome (Figs 1a, S4).

To further test whether centromeric organization is consistent across *Humulus* accessions, we aligned (peri)centromeric sequences to two additional female reference genomes (Cascade and drHumLupu1) to examine the distribution of six (peri)centromeric repeats in various *Humulus* accessions. Major centromeric repeats, SaazCEN and SaazCRM1, were significantly enriched in centromeres of all *Humulus* accessions (Fig. S14). Centromere positions, based on the SaazCEN localization, were consistent across all four tested accessions on chromosomes 7 and 9, as well as the remaining chromosomes in Saaz, the male accession ‘10–12’, and drHumLupu1. The distribution and localization of Saaz293, Saaz85, Saaz40, and HuluTR120 follows similar patterns, except Cascade. Notably, in Cascade all four satellites were underrepresented on chromosome 2. Chromosomes 1, 4, 6, and X displayed additional SaazCEN‐ and SaazCRM1‐enriched domains, a pattern that we hypothesize results from gaps in genome assembly (Fig. S14). In summary, the centromere organization across *Humulus* cultivars is consistent regarding the presence of two major satellites, SaazCEN and SaazCRM1. However, the abundance of chromosome‐specific (peri)centromeric satellites (Saaz293, Saaz85, Saaz40, and HuluTR120) varies among the studied accessions.

### Diversity of LTR retrotransposons in *Humulus* centromeres

The newly identified centromeric repeat SaazCEN with basic monomer units of 284 bp is part of LTR domains that are flanking the protein coding genes of CRM retrotransposons. Our analysis revealed that SaazCEN includes a 39 bp subunit located at the 3′ end, as observed in the dot plot (Fig. S15a,b). Most SaazCEN contains three or four 39 bp subunits, and their chromosomal distribution aligns with CRM density (Fig. S15c). We analyzed 6 Mb‐long regions along the centromeres (see the Materials and Methods section) of Saaz female and ‘10–12’ male using DANTE‐LTR. This analysis revealed five clades (Ale, Angela, Ikeros, SIRE, and TAR) of the Ty1/*Copia* family and TEs from chromovirus (Tekay and CRM) and nonchromovirus (Retand and Athila) lineages of the Ty3/*Gypsy* family in all centromeres (Fig. S16). Among Ty1/*Copia*, we found Angela to be the most abundant clade, although Ty1/*Copia* exhibited overall lower copy numbers compared to Ty3/*Gypsy* families. The lowest proportion of the Ty1/*Copia* family was found in the centromere of the Y chromosome (Fig. S16a). Within the Ty3/*Gypsy* family, Tekay and CRM, which possess chromodomains at the integrase C‐terminal region (Neumann *et al*., 2019), were the most dominant sequences (Fig. S16b). Genome‐wide analysis of retrotransposons revealed CRM at the centromeres of all tested *Humulus* accessions (Fig. S17). The distribution of CRMs outside the centromeric domain was rare. Based on LTR similarity, the youngest CRM copies were identified within the HlCENH3 binding domains, with the average insertion time estimated to range from 0.0 to 1.0 million years ago (Ma) (Figs S18, S19). This pattern is consistent again across the tested *Humulus* accessions, with the majority of CRM insertions within the past 1 million years (Fig. S20). By contrast, Tekay elements are generally older, with insertion ages between 0.0–10.0 Ma and are more dispersed within the centromeric region (Figs S18, S20). Interestingly, chromosomes 1, 2, 3, 5, 7, X, and Y display an expanded HlCENH3 binding domain (Figs S5, S18). The high density of recent CRM insertions in the immediate vicinity of HlCENH3 of chromosomes 1, 5, and Y indicates an expansion of CRMs and a potential shift in centromere position (Fig. S18).

### Autonomous and nonautonomous CRMs in *Humulus* centromeres

**Fig. 3:**
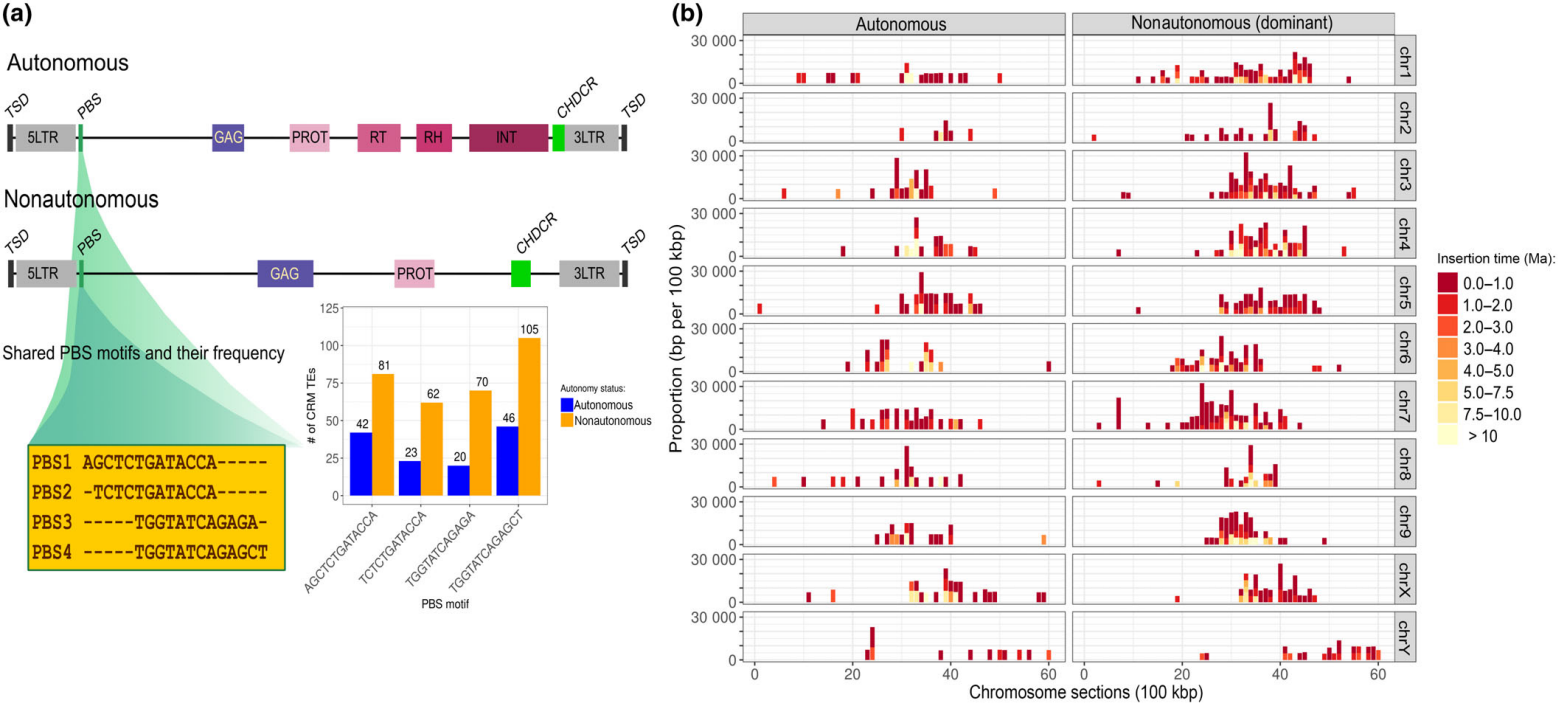
Characterization of CRM retrotransposons in the centromeres of *Humulus lupulus*. (a) The structures of autonomous and nonautonomous centromeric CRM retrotransposons in *H. lupulus*. Both retrotransposons have two direct long terminal repeats (5' long terminal repeat (LTR) and 3′ LTR) and a primer binding site (PBS). Shared PBS motifs are shown in the yellow box. PBS4 is the most frequent in nonautonomous CRM. Autonomous CRMs contain six protein domains: GAG, protease (PRO), reverse transcriptase (RT), RNase H (RH), integrase (INT), and chromodomain (CHDCR type). By contrast, nonautonomous CRMs lack all three domains (RT, RH, and INT). Both retrotransposons are flanked by target site duplication (TSD). (b) Distribution of autonomous and nonautonomous (dominant) CRM retrotransposons and their insertion time (color scale from red to yellow) across *Humulus* centromeres. Note the recent insertions of both CRM groups in the positions of CENH3 enrichment (see Supporting Information Fig. S18).

We identified a total of 671 CRM copies in *Humulus* centromeres, categorized as autonomous and nonautonomous CRM retrotransposons (noaCRM). Autonomous CRMs are composed of one large open reading frame (ORF) that encodes all canonical proteins required for retrotransposition. These include the GAG protein, which forms virus‐like particles in which reverse transcription takes place, reverse transcriptase (RT), RNase H (RH), integrase (INT), chromodomain (CHDCR), and protease (Fig. 3a). The dominant noaCRM in *H. lupulus* identified in this work lacks essential proteins, namely RT, RH, and INT (Fig. 3a; Langdon *et al*., 2000; Nagaki *et al*., 2005). Minor noaCRMs, a subclass of dominant noaCRM in *H. lupulus*, lack further proteins, resulting in an incomplete ORF (Fig. S21). Autonomous and noaCRMs represent 189 and 482 of all identified CRMs, respectively. Among noaCRMs, 446 copies are dominant, while 36 are minor elements. From 671 CRMs, the primer binding site (PBS) motif was detected in 169 autonomous CRMs and 374 noaCRMs, accounting for 80.9% of both groups. Comparison of the PBS sequence structure between noaCRMs and autonomous CRMs revealed that, of 543 TEs, 449 (82.7%) belong to one of the four dominant PBS motifs. The sequences of 5′ LTR in groups with similar PBS motifs are identical in both autonomous and noaCRMs (Fig. 3a), suggesting that reverse transcription and even replication may be triggered by the same mechanism in both CRM groups. These findings provide evidence of autonomous CRM‐dependent retrotransposition of noaCRMs. Comparison of insertion time and CRM distribution across individual centromeres showed no large differences between autonomous and nonautonomous categories (Fig. 3b). Phylogenetic and clustering analysis of both autonomous CRMs and noaCRMs, along with insertion time data, revealed that the most recent insertions (0.0–1.0 Ma) occurred within noaCRMs or in subdomains of autonomous CRMs (Fig. S22a,b). Finally, SaazCEN repeats are part of the majority of both CRM categories (94.4%), including autonomous and noaCRMs (Fig. S23). Based on the HlCENH3 affinity and the localization of the CENH3 binding domain within CRMs, we found that the HlCENH3 binding domains preferentially associate with noncoding regions (spacer) of both CRM categories. The second highest frequency of interactions was observed within LTR regions (Fig. S24).

### The segregation of chromosome 2 during mitosis and meiosis

**Fig. 4:**
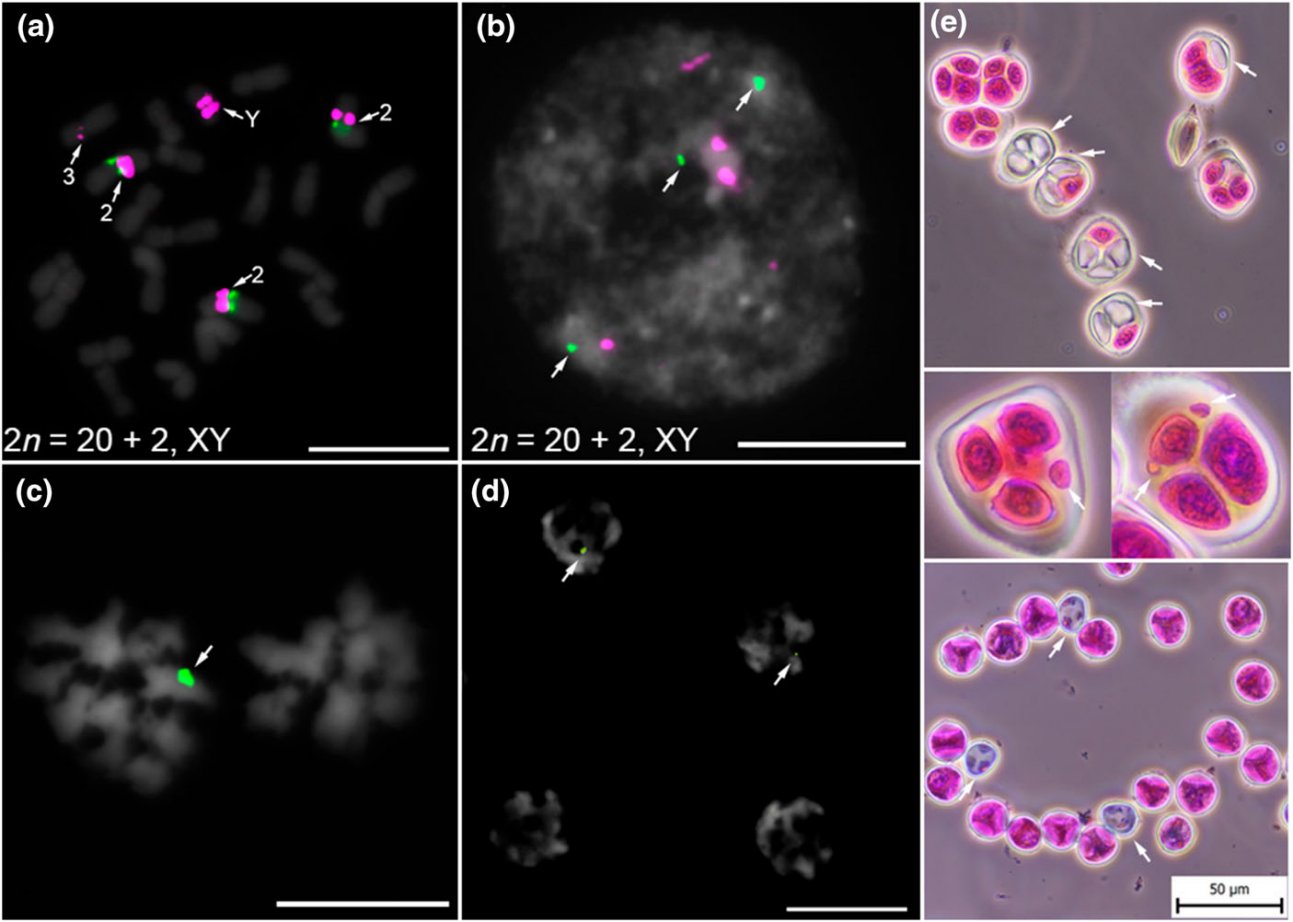
Effects of chromosome 2 aberrant segregation on mitosis, meiosis, and pollen grain development in Lib male *Humulus lupulus*. (a) Aneuploidy (2*n* + 2) in *H. lupulus* is characterized by three copies of chromosome 2 identified by centromeric satellite Saaz293 (green) and HuluTR120 (magenta). Bar, 10 μm. (b) Three green signals of satellite Saaz293 (arrows) were observed in the male nucleus. The presence of five HuluTR120 signals (magenta) suggests aneuploidy within the nucleus. Bar, 10 μm. (c) Unequal segregation of chromosomes during anaphase I. Arrow indicates single signal of Saaz293 (green). Bar, 10 μm. (d) Asymmetrical distribution of Saaz293 signals (green, arrows) observed during telophase II. Chromosomes and nuclei were counterstained with 4′,6‐diamidino‐2‐phenylindole (DAPI). Bar, 10 μm. (e) Irregularities in meiosis and pollen grain development observed in three male *H. lupulus* accessions. Inviable microspores (gray, arrows), micronuclei (one or two per cell, arrows), viable pollen grains (stained purple), and nonviable pollen grains (gray, arrows). Bar, 50 μm. Frequencies of meiotic errors and inviable microspores are detailed in Supporting Information Tables S6 and S7.

The term somatic aneuploidy or aneusomaty refers to the nuclear condition of plant meristems in which additional euploid or aneuploid chromosome numbers of certain chromosomes occur within one individual. This occurs in addition to the regular mitoses with the diploid chromosome number (reviewed in D'amato, 1985). We tested the level of somatic aneuploidy in Lib male accession and observed chromosome number variation among the leaf tissue within one plant (2*n*; 2*n* + 2). We identified an accessory chromosome 2 in metaphase (2*n* + 2) based on the simultaneous localization of newly identified satellites Saaz293 and HuluTR120 (Fig. 4a) and 5S rDNA (Fig. S25). We observed varying levels of aneusomaty for chromosome 2 (two to three foci of Saaz293) in male interphase nuclei (Figs 4b, S26a). Interestingly, we detected aneusomaty exclusively in male hop plants, whereas female individuals consistently exhibited even numbers of Saaz293, with minimal or no variability per plant (Fig. S26b).

Based on previous observations of non‐Mendelian inheritance in hop plants (Easterling *et al*., 2018, 2020), we screened PMCs at meiosis I (metaphase and anaphase) and meiosis II (tetrad stage) in three hop genotypes (Lib male, dwarf, and wild hop II.). We found bivalents excluded from the metaphase plate during metaphase I, unbalanced segregation of homologous chromosomes to the opposite poles of the cell, and the presence of lagging chromosomes in late anaphase I and telophase I (Fig. S27). FISH analysis of meiotic chromosomes in anaphase I (Figs 4c, S27e,f) and in telophase II (Fig. 4d) revealed an unequal distribution of Saaz293 foci. One Saaz293 locus observed in anaphase I suggests potential nondisjunction, leading to chromosome mis‐segregation (Fig. 4c). As a result of this division, only two Saaz293 foci were detected in telophase II (Fig. 4d). To assess the impact on microsporogenesis, we analyzed three *H. lupulus* male accessions (Lib male, F_1_ progeny (Osvald's clone 72 x Lib male), and wild hops I.). In addition to balanced tetrad formation, we observed unreduced cells (monads, dyads, and triads) ranging from 5.36% to 9.24%, micronuclei (0.40–0.87%), and inviable microspores (2.50–6.96%) across the three *Humulus* accessions, resulting in a total of 10.57 to 12.72% meiotic errors (Fig. 4e; Table S6). Despite the number of meiotic abnormalities, significant reduction in pollen viability was observed only in the Lib male accession (Fig. 4e; Table S6), which exhibited 3.95% inviable pollen grains. By contrast, the F1 male progeny (Osvald's clone 72 x Lib male) and wild hop I showed much lower rates of inviable pollen grains, with averages of 0.55% and 0.09%, respectively (Table S7). Meiotic abnormalities in Lib male may be more severe or functionally disruptive than in other genotypes (F1 male progeny and wild hop I.) which may possess more stable chromosome constitutions. Based on these cytogenetic analyses, we conclude that chromosome 2 may be involved in the observed aneuploidy and hypothesize that it is involved in meiotic errors (Fig. 4). However, further investigation is needed to confirm such association.

## Discussion

Aberrant meiosis in plants can have both positive and negative consequences. Aside from aneuploidy, reduced fertility or sterility, altered seed development, and genetic instability, it also contributes to increased genetic diversity. Subsequently, balanced translocation (chromosomal exchange without any genetic material being lost or gained) can be transmitted through meiotic cell division, decreasing fertility and sometimes being passed as an unbalanced form to offspring. These processes make aberrant meiosis an important force in promoting speciation and adaptation (reviewed in De Storme & Mason, 2014; Zamariola *et al*., 2014). Previous studies of the *Humulus* genome revealed an unusual pattern of chromosome segregation and non‐Mendelian inheritance patterns during male meiosis in the North American hop variety (*H. lupulus* var. *neomexicanus*). The same study proposed that aberrant meiosis may be a natural feature, a consequence of breeding genetically divergent *Humulus* varieties, or a result of unusual centromere structure (Easterling *et al*., 2018, 2020). In this study, we assess centromere organization in hop and ask whether the aberrant chromosome segregation is linked to unusual centromere organization, the existence of dicentric chromosomes, or other genomic factors.

### Structural features of centromeric landscape in *H. lupulus*

The centromeres of *H. lupulus* lack the higher‐order repeats commonly found in most plants, such as *Cen178* in *Arabidopsis thaliana* (Wlodzimierz *et al*., 2023). ChIP‐seq analysis using HlCENH3 revealed that the centromeres of all *H. lupulus* chromosomes are primarily composed of SaazCRM1, CRM retrotransposons (the major centrophilic TE), and the SaazCEN repeat (Figs 1ac, 2b). This composition is consistent across all four tested accessions: Saaz, the male ‘10–12’, Cascade, and drHumLupu1. The basic centromeric monomer unit in *H. lupulus* is 284 bp, with short subarrays of 39 bp subunits at the 3′SaazCEN terminus. Although the role of these subunits remains unclear, the average monomer length is consistent with those of other species, which range from 23 bp in *Chionographis japonica* (Kuo *et al*., 2023) to 882 bp in *Pisum sativum* (Macas *et al*., 2023), and the higher‐order satellite array in *Vigna unguiculata* (Yang *et al*., 2023). The centromeric SaazCEN repeat is embedded within the LTR region of the centrophilic SaazCRM1 retrotransposon (Fig. S15). LTRs contain key functional regions, including the U3 enhancer, R and U5 regions (reviewed in Hassan *et al*., 2023). In *H. lupulus*, CENH3 preferentially binds to noncoding regions and the LTRs of CRMs (Fig. S24). Thus, we propose that SaazCRM1 carrying SaazCEN undergoes dynamic amplification and facilitates the targeting of SaazCEN (and new potentially novel variants) into centromeric regions. This integration into the centromere both supports and reflects functional adaptation to centromere activity, again in all four tested accessions. The integrase chromodomain of CRMs is thought to mediate their targeting to centromeric regions (Kordiš, 2005; Neumann *et al*., 2011). Although noaCRMs predominate in *Humulus* centromeres, sequence similarity in the PBS regions of both CRM categories (autonomous and noaCRMs) suggests that noaCRM relies on autonomous CRMs for their function. This dependency is further supported by their similarity to other protein domains. Because the integrase, possessing a putative targeting domain in the C‐terminus (Neumann *et al*., 2011), is present only in autonomous CRMs of *H. lupulus*, they are essential for the spreading of noaCRMs. However, the exact mechanism by which CRMs target centromeres and their preferential motifs remains poorly understood. Notably, we observed very recent transposition activity of noaCRM. The estimated insertion times of *Humulus* CRMs, ranging from 0.0 to 1.0 Ma, are consistent with those of CRR elements in rice (Nagaki *et al*., 2005) and CRW elements in wheat (Liu *et al*., 2008). In wheat, centromeres are primarily composed of retrotransposons (Li *et al*., 2013; Ahmed *et al*., 2023), with the *Quinta* element representing recent insertion in both diploid and hexaploid wheats compared to other CRW elements (Li *et al*., 2013). Similarly, the insertion times of *RLG_Cereba* and *RLG_Quinta* chromovirus families in einkorn wheat have been estimated to be 0.0–1.0 Ma (Ahmed *et al*., 2023), closely matching those of the centrophilic SaazCRMs in *H. lupulus* and suggesting evolutionary parallels. Our findings underscore the potential role of chromoviruses in centromere function and the preservation of centromeric integrity, as reviewed in Lisch, 2013; Naish & Henderson, 2024. Centromere repeats are known to be transcribed into noncoding centromere RNAs (Talbert & Henikoff, 2018), which can form R‐loops composed of a DNA:RNA duplex and a displaced single‐strand DNA. In maize, R‐loops predominantly originate from TEs and are formed within CENH3‐binding regions, particularly in CRM1 and CRM2 retrotransposons, which are strongly associated with R‐loop formation. This structure may contribute to the maintenance of a stable chromatin environment conducive to CENH3 localization and centromere function (Liu *et al*., 2020, 2021). It will be of particular interest to investigate whether similar mechanisms operate in *H. lupulus*, especially in relation to SaazCEN and SaazCRM1 in future studies.

Two types of centromeres, consisting of SaazCEN and SaazCRM1 only (first type – chromosomes 1, 4, 5, 7, and X), or additionally carrying Saaz293, Saaz40, and Saaz85 (second type – chromosomes 2, 3, 6, and 8). Physical localization of the latter three centromeric repeats confirmed their colocalization only on chromosomes 2 and 8. Notably, chromosome Y possesses HuluTR120, enriched within the CENH3 binding domain (Fig. 1), making this satellite evolutionarily important during sex chromosome divergence (Figs 1e,f, S8, S11). The presence of additional centromeric satellites is not surprising as most plant species typically have centromeres composed of several satellite tandem arrays (reviewed in Naish & Henderson, 2024). However, it remains elucidated whether these repeats, and major CRMs together with SaazCEN, are shared with other species in the Cannabaceae family. It is intriguing to speculate that the burst of CRM elements in *H. lupulus* centromeres may have occurred in the context of chromosome rearrangements and genome evolution, particularly in the context of sex chromosome differentiation (Akagi *et al*., 2025). Lynch *et al*. (2024) reported that in *Cannabis sativa*, the centromere of chromosome 7 is enriched with Harbinger TEs, with a divergence time estimated at *c*. 20 Ma. They propose that these TEs contribute to genome rearrangements and centromeric evolution between *C. sativa* and *H. lupulus*. Similarly, Zhang *et al*. (2023) identified centromeric satellite Hssat1 in *Humulus japonicus*, which is present in the centromeres of all chromosomes except for two Y chromosomes. The authors suggested that sex chromosomes in *H. japonicus* originated from centric fission events, potentially leading to the loss of centromere‐specific satellites on the Y chromosomes. In this study, however, we did not find any sequences homologous to Hssat1. Although centromere evolution in related species remains largely unresolved, it is becoming apparent that the two Y chromosomes in *H. japonicus*, which lack major centromeric repeats, may illustrate a case of genomic convergence in centromere evolution comparable to *H. lupulus*. Given the relatively recent insertion of CRM elements in the centromeric regions identified in this study, we propose that these elements in *H. lupulus* and potentially in the Y chromosomes of *H. japonicus* may play a crucial role in maintaining centromere integrity, similar to the function of *Cereba* and *Quinta* in einkorn wheat (Ahmed *et al*., 2023). Therefore, a comprehensive understanding of centromere organization in closely related species, such as *C. sativa* and *H. japonicus*, will shed light on the evolutionary dynamics of centromere structure and function.

As mentioned above, the centromeric region of chromosome 2 is enriched with the Saaz293 satellite array (Fig. 1d). The centromere of this chromosome exhibits a higher‐order array structure compared to other autosomes (Figs 1, S4). Remarkably, chromosome 2 shows low enrichment for both major centromeric repeats, SaazCEN and SaazCRM1 (Fig. S5). A similar centromere composition has been reported in sunflower, where all chromosomes are enriched with LINE elements, with the exception of one chromosome that harbors centromeric satellites (Nagaki *et al*., 2005). This leads us to speculate that the Saaz293 may have diverged from the last common ancestor, hinting at a potential for neocentromere formation. A similar scenario has been proposed for the unique centromeric landscape in potato, which has two types of centromeres (Gong *et al*., 2012). While the origin of the newly identified repeats remains unclear, our findings point to a possible involvement of chromosome 2 in the non‐Mendelian segregation patterns reported by Easterling *et al*. (2018, 2020).

### Aberrant segregation patterns and distinctive characteristics of chromosome 2

We detected aneuploidy (2*n* + 2) with one accessory chromosome 2 in the Lib male accession, consistent with previously reported meiotic abnormalities. These metaphases were identified based on the distribution of Saaz293 and HuluTR120 (Fig. 4a). We confirmed aberrant chromosome segregation of chromosome 2 by screening PMCs during meiosis I and II (Fig. 4c,d). These observations corroborate the non‐Mendelian ratio of 5S rDNA at the tetrad stage (Easterling *et al*., 2018), as both satellites Saaz293 and HuluTR120 are localized on chromosome 2, which also harbors the 5S rDNA. Using the available FISH DNA markers for individual chromosomes, we were unable to identify the second accessory chromosome in metaphase (2*n* + 2). We hypothesize that this chromosome may originate from homologous chromosome pair 3, which exhibits structural chromosome heterozygosity, as indicated by the presence of HuluTR120 in only one chromosome of the pair (Figs 1e,f, S12d,e). This structural heterozygosity likely results in irregular chromosome pairing, leading to meiotic irregularities (Ostberg *et al*., 2013). To test this hypothesis, it will be necessary to develop unique DNA oligo painting probes, as previously described in *Silene latifolia* (Bačovský *et al*., 2020) or chickpea (Doležalová *et al*., 2022), and reviewed by Jiang (2019) and Hobza *et al*. (2024).

Although the centromeres of chromosomes 8 and Y are composed of satellites Saaz40 and Saaz85, and HuluTR120, respectively, we did not observe non‐Mendelian segregation of these chromosomes. Interestingly, the utility of Saaz293 lies in its unique chromosomal localization (compared to 5S rDNA or HuluTR120) that allows easy tracking of non‐Mendelian segregation patterns across various hop accessions in future studies. In combination with techniques like high‐content imaging and cell population screening (Hobza *et al*., 2024), Saaz293 could serve as a promising marker for hop breeding, providing a convenient test for tissue culture stability and a way to monitor spontaneous chromosome instability during *in vitro* cultivation (Abugammie *et al*., 2024). In the context of previous studies, we propose that the unique centromere structure of chromosome 2, namely the unusual abundance of the additional tandem repeat (Saaz293), plays a crucial role in chromosome segregation and the observed non‐Mendelian segregation patterns (Fig. 4). We hypothesize that non‐Mendelian segregation patterns result from either nondisjunction of two chromosome pairs or lagging chromosomes during metaphase (Fig. S28). Nondisjunction or lagging chromosomes may result in the observed division defects and the formation of aneuploid cells and micronuclei, collectively reducing pollen viability.

This study provides the first detailed survey of the centromeric landscape of the dioecious plant *H. lupulus* and sheds new light on previously published non‐Mendelian segregation patterns. The precise localization of centromeric repeats SaazCEN and SaazCRM1 on the Y chromosome in the Lib male further refines the position of the HSR1 subtelomeric probe (Fig. S11) compared to what was previously described by Divashuk *et al*. (2011). The HSR1 positive regions are located in the subtelomeric region on the p‐arm of the Y chromosome and in both the pericentromeric and subtelomeric regions on the X chromosome. These results can be used to posit the existence of PAR on the p‐arm of the Y chromosome. The use of self‐genomic *in situ* hybridization (self‐GISH), where genomic DNA from the same species is hybridized to its own chromosomes (She *et al*., 2007), highlighted the male‐specific region of the Y chromosome in *H. lupulus* while excluding the PAR at the p‐arm terminus. This finding further supports our results (Razumova *et al*., 2023). Additionally, our results enable the testing of other male accessions for non‐Mendelian segregation and pave the way for understanding the evolutionary processes that led to species divergence within the Cannabaceae family.

## Competing interests

None declared.

## Author contributions

VB, LH, VH, PJ, JŠ and RH planned and designed research. LH, VB and PN performed experiments. RČ and PJ analyzed the data. LH, VB and PJ wrote the main text of the manuscript. HT, AT and TI performed genome sequence and assembly of *Humulus* plants. TA and EO provided sequencing data. JP provided plant material. LH and PJ contributed equally to this work. All authors read and approved the final version of the manuscript.

## Disclaimer

The New Phytologist Foundation remains neutral with regard to jurisdictional claims in maps and in any institutional affiliations.

## Supporting information


**Fig. S1** CENH3 blast‐n of *Humulus lupulus* and multiple alignment of the HlCENH3 sequence with other plant species.
**Fig. S2** The sequence variability of the HlCENH3 gene in *Humulus lupulus*.
**Fig. S3** The localization of HlCENH3 antibody in interphase nuclei of Saaz female *Humulus lupulus*.
**Fig. S4** Centromere characterization of each chromosome in the *Humulus lupulus* genome.
**Fig. S5** The distribution of major centromeric repeat arrays for each chromosome in the *Humulus lupulus* genome.
**Fig. S6** Distribution of major centromeric repeats on metaphase chromosomes of *Humulus lupulus* Saaz female.
**Fig. S7** Male karyotype of *Humulus lupulus*, 2*n* = 20, XY.
**Fig. S8** Detailed analysis of centromere composition in three different *Humulus lupulus* male accessions.
**Fig. S9** Localization of the centromeric satellite Saaz40 and 45S rDNA on chromosome 8 in *Humulus lupulus*.
**Fig. S10** Localization of the centromeric satellite Saaz85 and 45S rDNA on chromosome 8 in *Humulus lupulus*.
**Fig. S11** Chromosome pairing and organization during diakinesis in *Humulus lupulus* Lib male.
**Fig. S12** Distribution of major centromeric repeats on metaphase chromosomes of *Humulus lupulus* Lib male.
**Fig. S13** Dot plot analysis and sequence similarity of three major centromeric satellites, Saaz85, Saaz293, and Saaz40, which are specific for chromosomes 2, 3, 6, and 8 in *Humulus lupulus*.
**Fig. S14** The distribution of centromeric repeats in *Humulus lupulus* accessions (Saaz, male ‘10–12’, Cascade, and drHumLupu1).
**Fig. S15** Sequence analysis of the SaazCEN repeat localized within LTR regions of *Humulus lupulus* CRMs.
**Fig. S16** LTR retrotransposons composition of all centromeres of *Humulus lupulus* genome.
**Fig. S17** The distribution of seven transposable element clades (Athila, CRM, Galadriel, Ogre, Reina, Retand, and Tekay) of Ty3/*Gypsy* family in *Humulus lupulus* accessions (Saaz, male ‘10–12’, Cascade, and drHumLupu1).
**Fig. S18** Distribution and estimated insertion time of Ty1/*Copia* and Ty3/*Gypsy* LTR retrotransposons families across all chromosomes in the *Humulus lupulus* centromere.
**Fig. S19** Insertion times of autonomous (red), dominant nonautonomous (green), and minor nonautonomous (blue) CRM retrotransposons across all chromosomes in the *Humulus lupulus* genome.
**Fig. S20** Insertion time of the Ty3/*Gypsy* family of LTR retrotransposons in *Humulus lupulus* accessions.
**Fig. S21** The overall comparison and sequence length of centromeric Ty3/*Gypsy* CRM retrotransposons in *Humulus lupulus*.
**Fig. S22** Phylogenetic analysis of autonomous and nonautonomous CRM retrotransposons in the *Humulus lupulus* centromere.
**Fig. S23** Distribution of the centromeric repeat SaazCEN within autonomous and nonautonomous CRM retrotransposons in the *Humulus lupulus* centromere.
**Fig. S24** Localization of HlCENH3 summits within CRM retrotransposons in the *Humulus lupulus* centromere.
**Fig. S25** Localization of satellite repeats on chromosomes 2 and 8 in *Humulus lupulus*.
**Fig. S26** Positioning of chromosome 2 within the interphase nucleus of male and female *Humulus lupulus*.
**Fig. S27** Meiotic abnormalities observed during male meiosis.
**Fig. S28** Schematic model of chromosome 2 aberrant segregation in *Humulus lupulus*.
**Methods S1** Extraction of DNA, genome sequencing, and characterization of repetitive DNA.
**Notes S1** Repeatome analysis of Saaz female and Lib male of *Humulus lupulus*.
**Table S1** List of *Humulus lupulus* plants used in this study.
**Table S2** List of primers used for PCR and preparation of FISH probes specific to *Humulus lupulus*.
**Table S3** Enzyme mixture used for the digestion of young leaves of *Humulus lupulus*.
**Table S4** Genomic fraction of repetitive DNA in the *Humulus lupulus* Saaz female and Lib male genomes.
**Table S5** Tandem repeats in *Humulus lupulus* Saaz female and Lib male genomes identified using the RepeatExplorer2 pipeline.
**Table S6** Number and frequency of meiotic abnormalities in three male accessions of *Humulus lupulus*.
**Table S7** Number and frequency of viable and inviable pollen grains in three male accessions of *Humulus lupulus*.
Please note: Wiley is not responsible for the content or functionality of any Supporting Information supplied by the authors. Any queries (other than missing material) should be directed to the *New Phytologist* Central Office.

## Data Availability

All sequencing data are available in the European Nucleotide Archive (ENA) under the accession number PRJEB81858. Reference genomes of *H. lupulus* are deposited in the DDBJ database (BioProject IDs PRJDB17941, PRJDB17942, and PRJDB18715). Raw data is freely available in the Zenodo data repository doi: 10.5281/zenodo.15640256.
