# Supplementary material for: Dynamic patterns of repeats and retrotransposons in the centromeres of Humulus lupulus L
Source: New Phytol. 2025 Jul 15;247(6):2766–80. doi: 10.1111/nph.70380 (PMC12371157; doi:10.1111/nph.70380)
Supplement: Supplementary file 1 — Fig. S1 CENH3 blast‐n of Humulus lupulus and multiple alignment of the HlCENH3 sequence with other plant species. Fig. S2 The sequence variability of the HlCENH3 gene in Humulus lupulus. Fig. S3 The localization of HlCENH3 antibody in interphase nuclei of Saaz female Humulus lupulus. Fig. S4 Centromere characterization of each chromosome in the Humulus lupulus genome. Fig. S5 The distribution of major centromeric repeat arrays for each chromosome in the Humulus lupulus genome. Fig. S6 Distribution of major centromeric repeats on metaphase chromosomes of Humulus lupulus Saaz female. Fig. S7 Male karyotype of Humulus lupulus, 2n = 20, XY. Fig. S8 Detailed analysis of centromere composition in three different Humulus lupulus male accessions. Fig. S9 Localization of the centromeric satellite Saaz40 and 45S rDNA on chromosome 8 in Humulus lupulus. Fig. S10 Localization of the centromeric satellite Saaz85 and 45S rDNA on chromosome 8 in Humulus lupulus. Fig. S11 Chromosome pairing and organization during diakinesis in Humulus lupulus Lib male. Fig. S12 Distribution of major centromeric repeats on metaphase chromosomes of Humulus lupulus Lib male. Fig. S13 Dot plot analysis and sequence similarity of three major centromeric satellites, Saaz85, Saaz293, and Saaz40, which are specific for chromosomes 2, 3, 6, and 8 in Humulus lupulus. Fig. S14 The distribution of centromeric repeats in Humulus lupulus accessions (Saaz, male ‘10–12’, Cascade, and drHumLupu1). Fig. S15 Sequence analysis of the SaazCEN repeat localized within LTR regions of Humulus lupulus CRMs. Fig. S16 LTR retrotransposons composition of all centromeres of Humulus lupulus genome. Fig. S17 The distribution of seven transposable element clades (Athila, CRM, Galadriel, Ogre, Reina, Retand, and Tekay) of Ty3/Gypsy family in Humulus lupulus accessions (Saaz, male ‘10–12’, Cascade, and drHumLupu1). Fig. S18 Distribution and estimated insertion time of Ty1/Copia and Ty3/Gypsy LTR retrotransposons families across all [file NPH-247-2766-s001.pdf]

New Phytologist

New Phytologist Supporting Information

Article title: **Dynamic Patterns of Repeats and Retrotransposons in the Centromeres of *Humulus lupulus* L.**

Authors: Lucie Horáková[1,2], Pavel Jedlička[1], Radim Čegan[1], Pavla Navrátilová[3], Hiroyuki Tanaka[4], Atsushi Toyoda[5], Takehiko Itoh[4], Takashi Akagi[6], Eiichiro Ono[7], Vojtěch Hudzieczek[1], Josef Patzak[8], Jan Šafář[3], Roman Hobza[1*], Václav Bačovský[1*]

Article acceptance date: 19 June 2025

The following Supporting Information is available for this article:

Fig. S1 CENH3 blast-n of *Humulus lupulus* and multiple alignment of the HlCENH3 sequence with other plant species. The position of the selected peptide for polyclonal antibody of HlCENH3 is shown in pink, the position of other histone domains - alphaN, alpha1, alpha2, alpha3 and linker are shown in orange, green and light green, respectively.

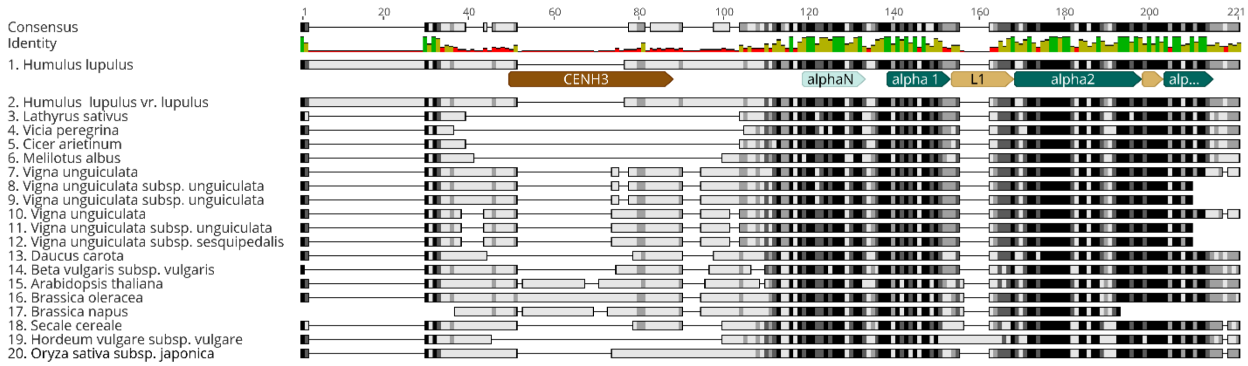

New Phytologist

Fig. S2 The sequence variability of the HICENH3 gene in *Humulus lupulus*. Eight sequences were amplified from colony PCR. The HICENH3 gene display little or no variability within the *H. lupulus* genome.

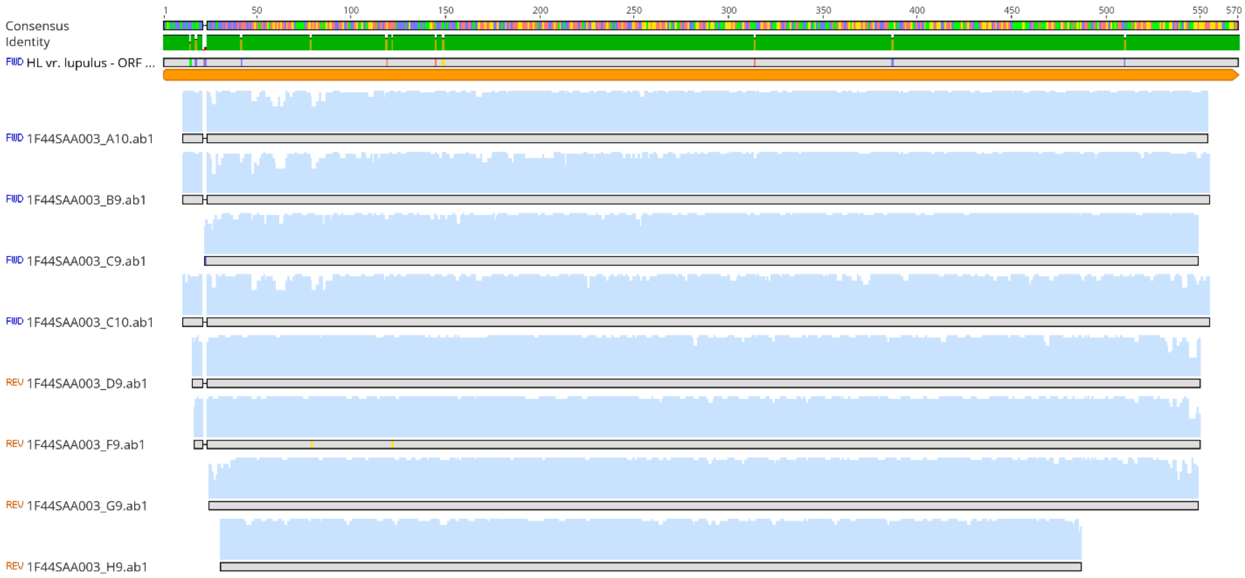

Fig. S3 The localization of HlCENH3 antibody in interphase nuclei of Saaz female *Humulus lupulus*. Immunostaining with HlCENH3 displays distinct signals (green) in interphase nuclei. Nuclei were derived from two independent experiments. Interphase nuclei were counterstained with DAPI. Scale bar = 10 µm.

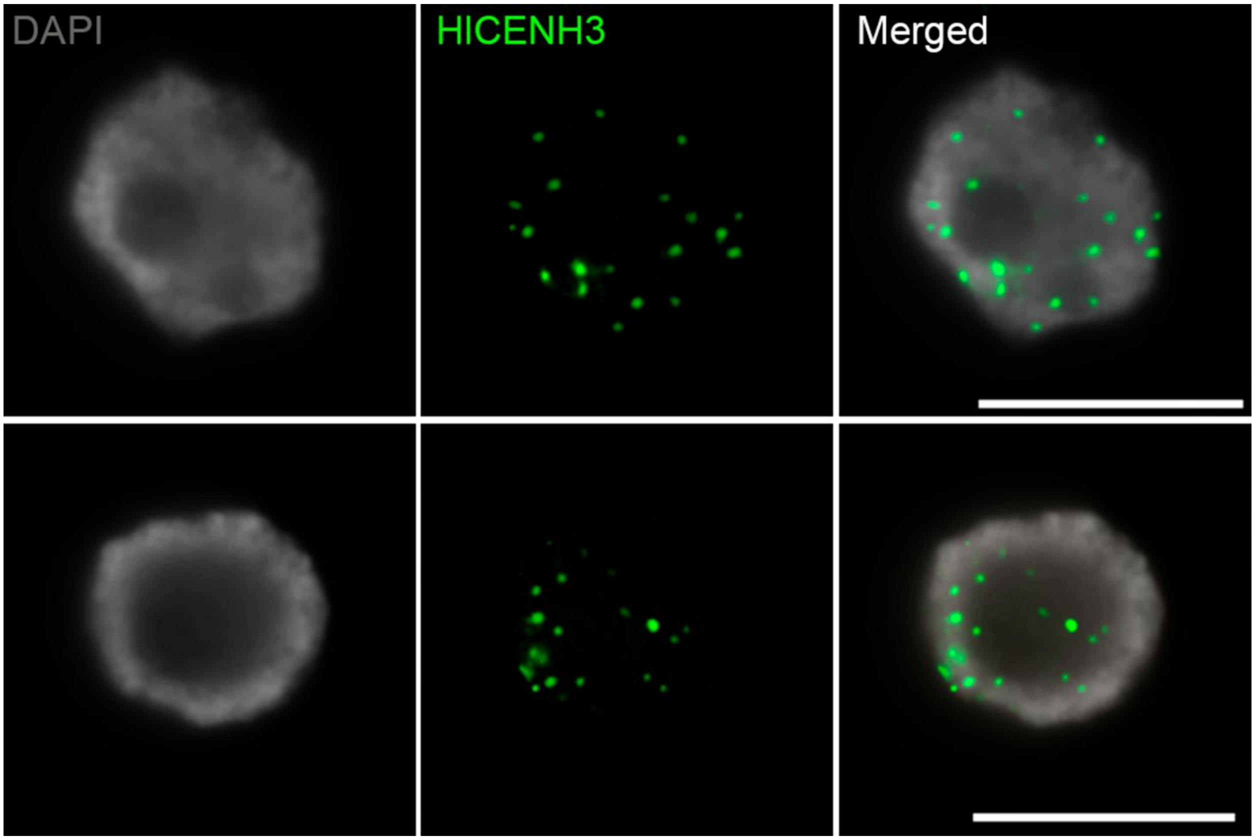

Fig. S4_part 1 Centromere characterization of each chromosome in the *Humulus lupulus* genome. Heatmaps display pairwise sequence identity with dot plots revealing organization on all chromosomes colored by percent identity. The first three lines compare HICENH3 ChIP-seq data replicates (green and blue) against the input control. The subsequent four lines show the detailed distribution of long terminal repeat (LTR) retrotransposons (Copia and Gypsy) and centromere-specific repeats SaazCEN and SaazCRM1.

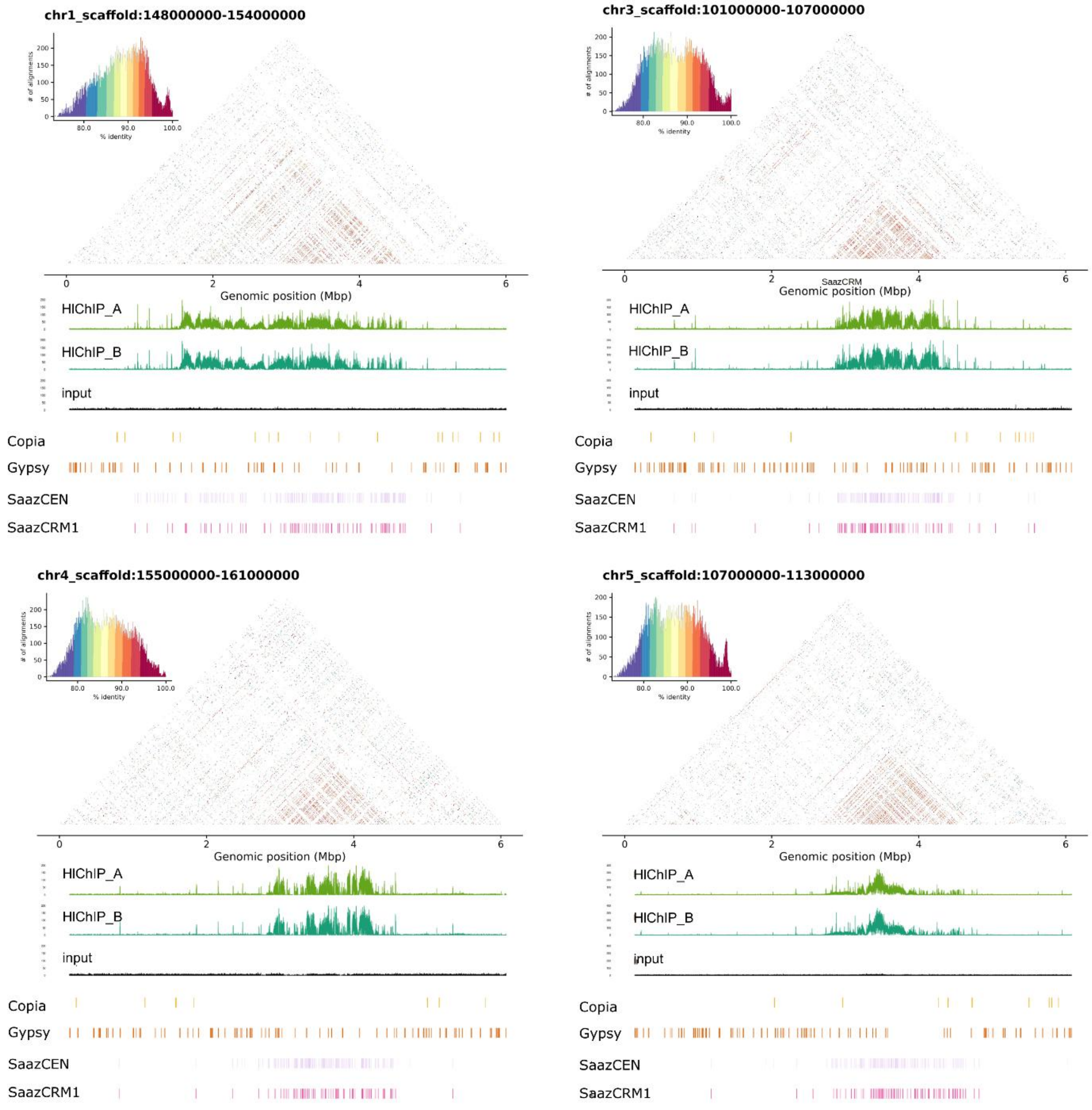

Fig. S4_part 2 Centromere characterization of each chromosome in the *Humulus lupulus* genome. Heatmaps display pairwise sequence identity with dot plots revealing organization on all chromosomes colored by percent identity. The first three lines compare HICENH3 ChIP-seq data replicates (green and blue) against the input control. The subsequent four/five lines show the detailed distribution of LTR retrotransposons (Copia and Gypsy) and centromere-specific repeats SaazCEN, SaazCRM1, and Saaz40.

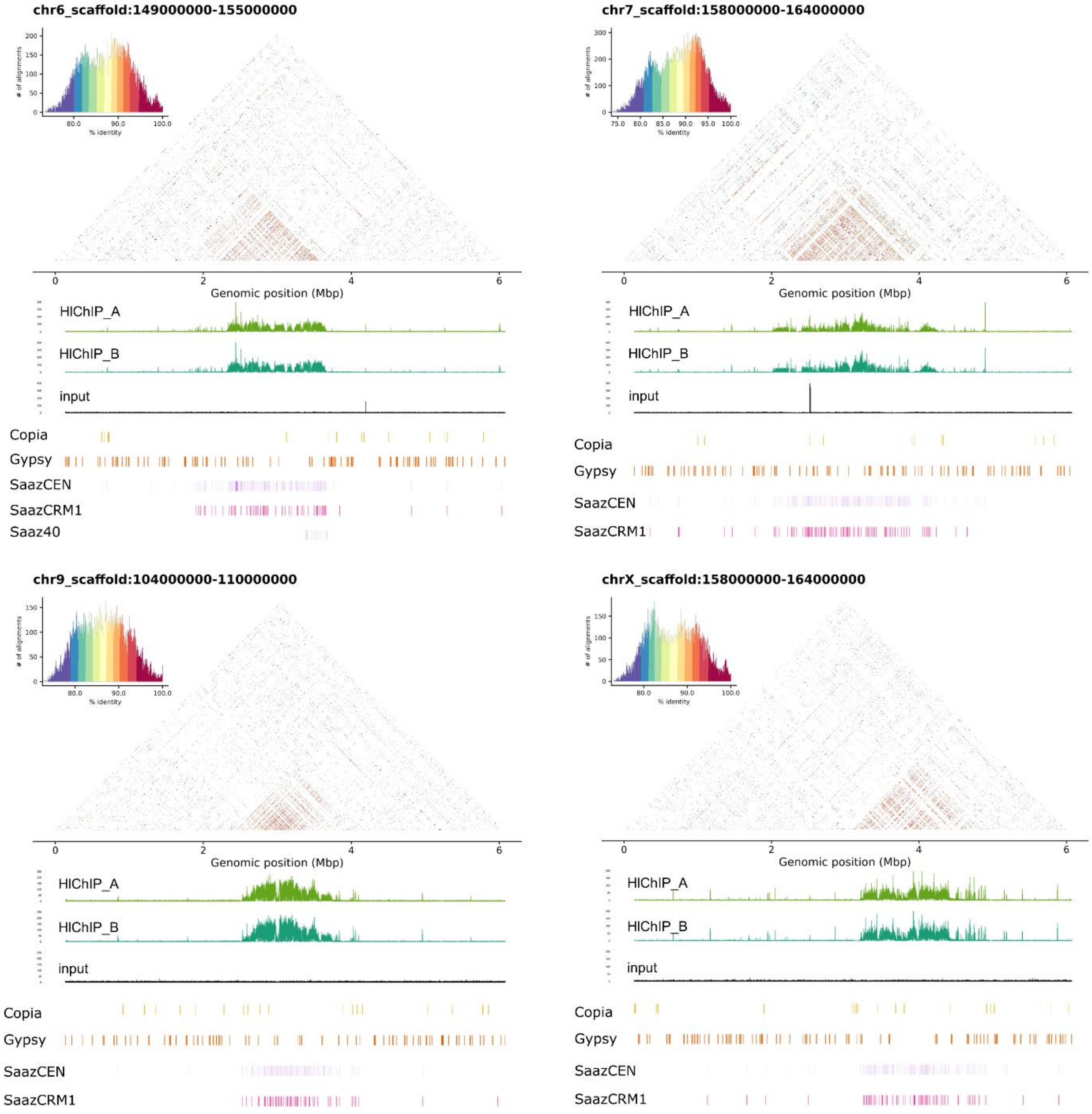

Fig. S5_part 1 The distribution of major centromeric repeat arrays for each chromosome in the *Humulus lupulus* genome. The first line represents the HlCENH3 binding region domain. The second line shows the distribution of major centromere-specific repeats across chromosomes. Main centromeric repeat SaazCEN and centromere-specific retrotransposon SaazCRM1, were found to be shared among all chromosomes. Note the specific distribution of three satellites Saaz40, Saaz85, and Saaz293 on chromosome 2. Interestingly, chromosome 2 possesses two HlCENH3-positive regions, consistent with the distribution of chromosome 2-specific centromeric satellite.

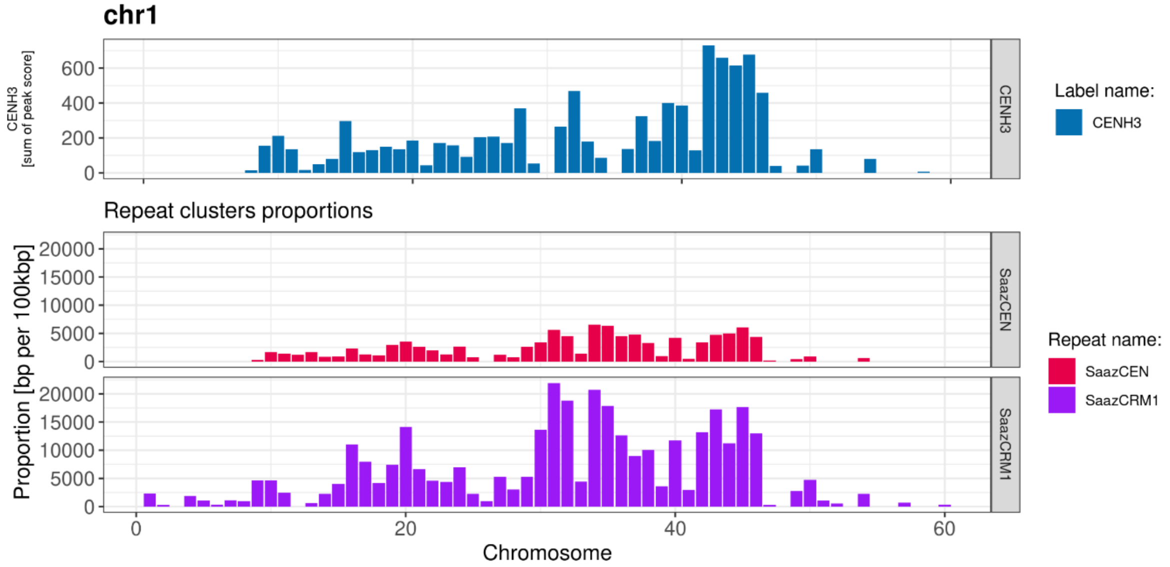

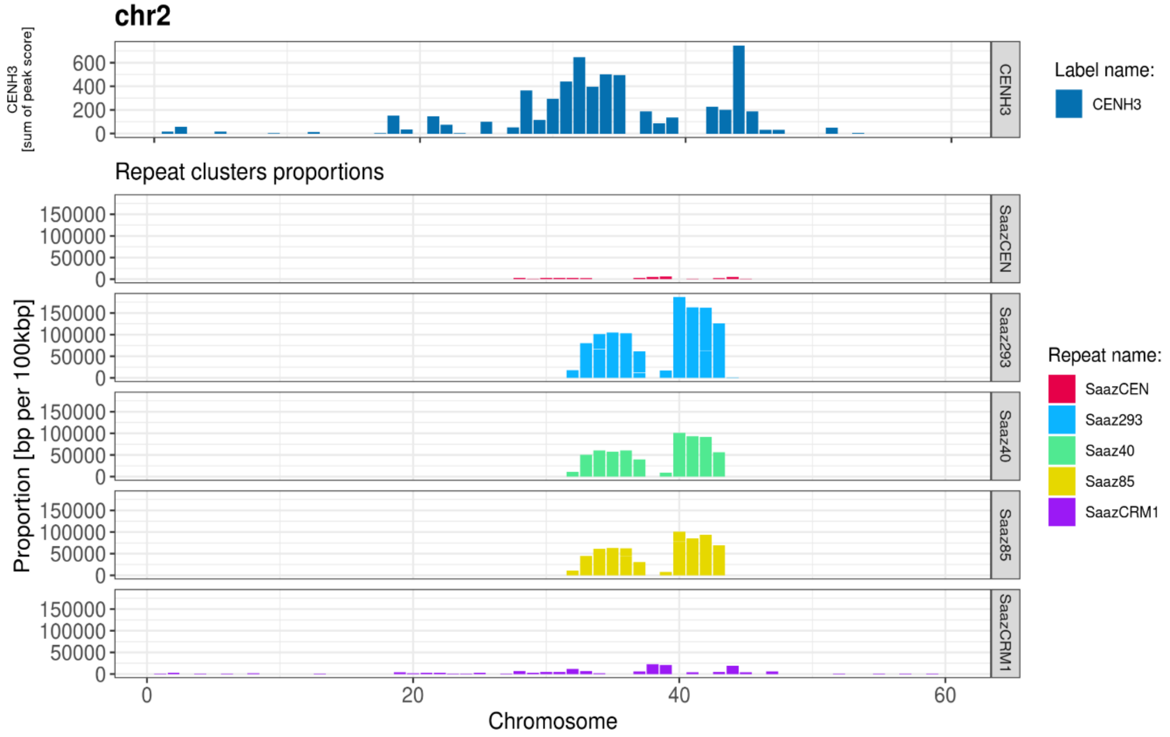

Fig. S5_part 2 The distribution of major centromeric repeat arrays for each chromosome in the *Humulus lupulus* genome. The first line represents the HICENH3 binding region domain. The second line shows the distribution of major centromere-specific repeats across chromosomes. Main centromeric repeat SaazCEN and centromere-specific retrotransposon SaazCRM1, were found to be shared among all chromosomes. Note the specific distribution of three satellites Saaz40, Saaz85, and Saaz293 on chromosome 3.

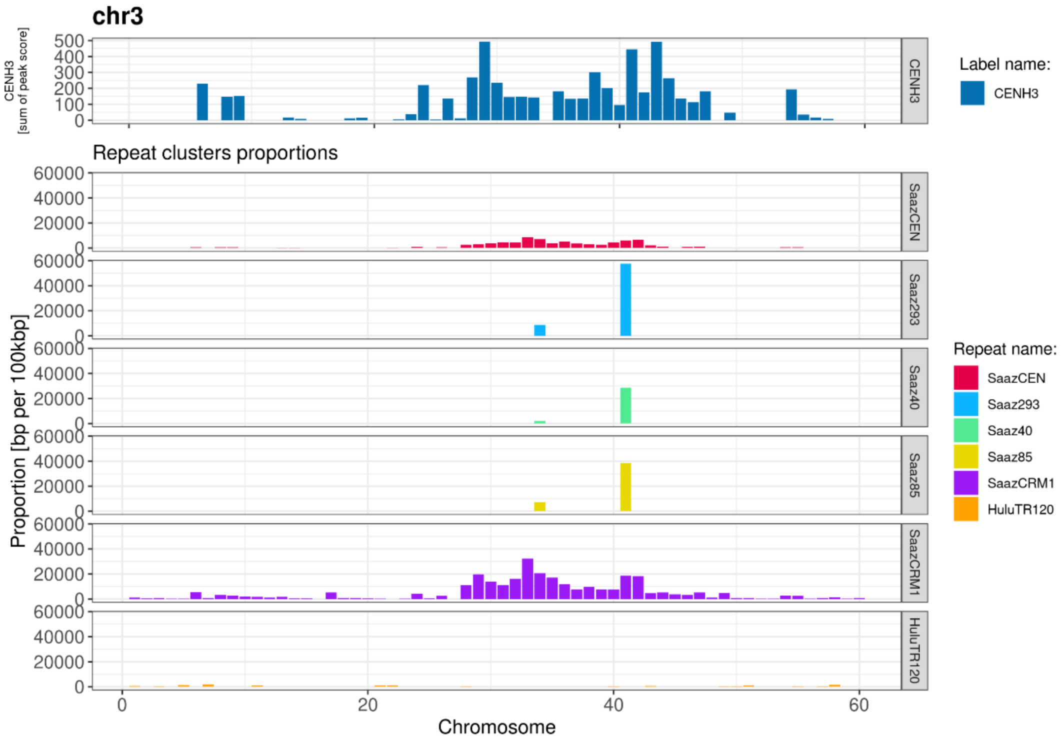

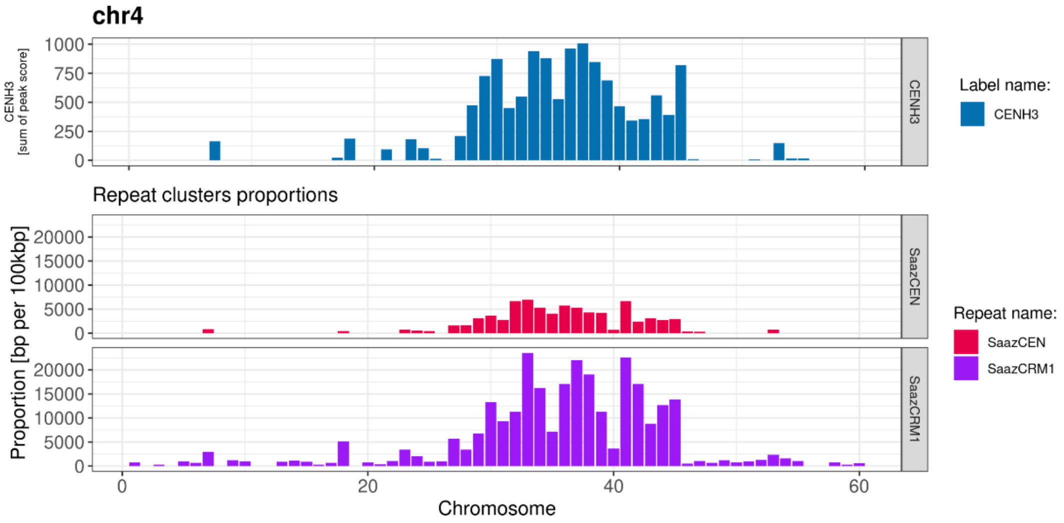

Fig. S5_ part 3 The distribution of major centromeric repeat arrays for each chromosome in the *Humulus lupulus* genome. The first line represents the HICENH3 binding region domain. The second line shows the distribution of major centromere-specific repeats across chromosomes. Main centromeric repeat SaazCEN and centromere-specific retrotransposon SaazCRM1, were found to be shared among all chromosomes. Note the specific distribution of three satellites Saaz40, Saaz85, and Saaz293 on chromosome 6.

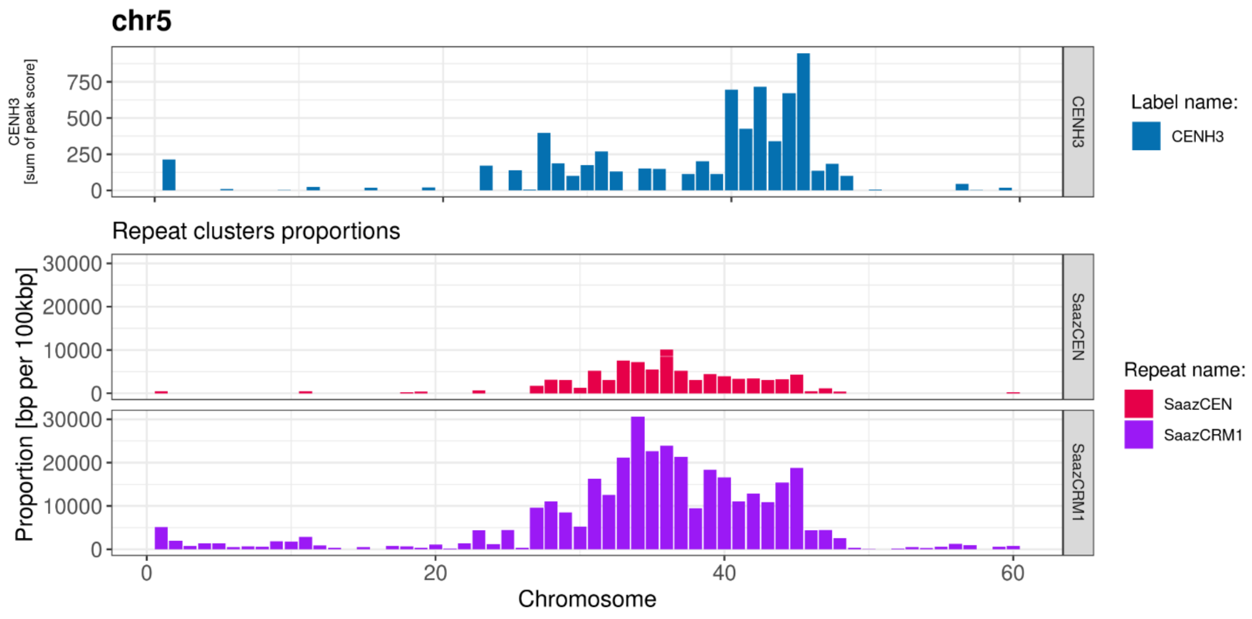

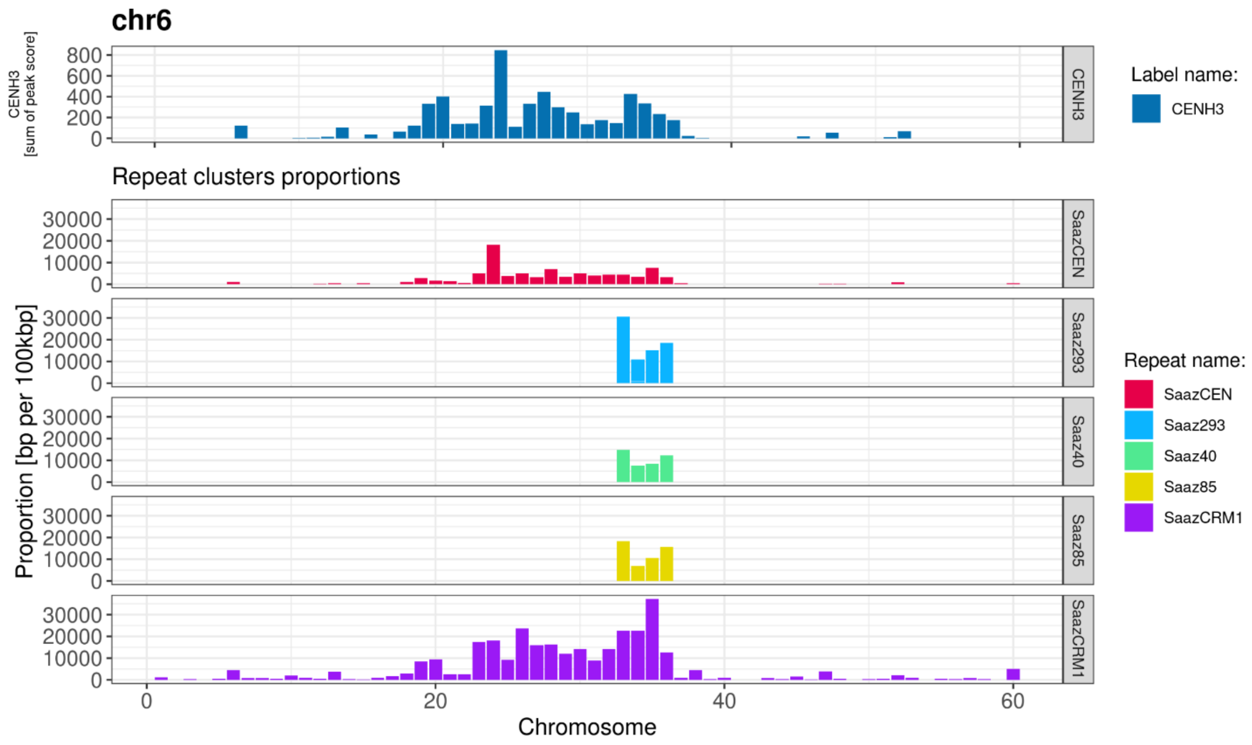

New Phytologist

Fig. S5_ part 4 The distribution of major centromeric repeat arrays for each chromosome in the *Humulus lupulus* genome. The first line represents the HlCENH3 binding region domain. The second line shows the distribution of major centromere-specific repeats across chromosomes. Main centromeric repeat SaazCEN and centromere-specific retrotransposon SaazCRM1, were found to be shared among all chromosomes. Note the specific distribution of three satellites Saaz40, Saaz85, and Saaz293 on chromosome 8.

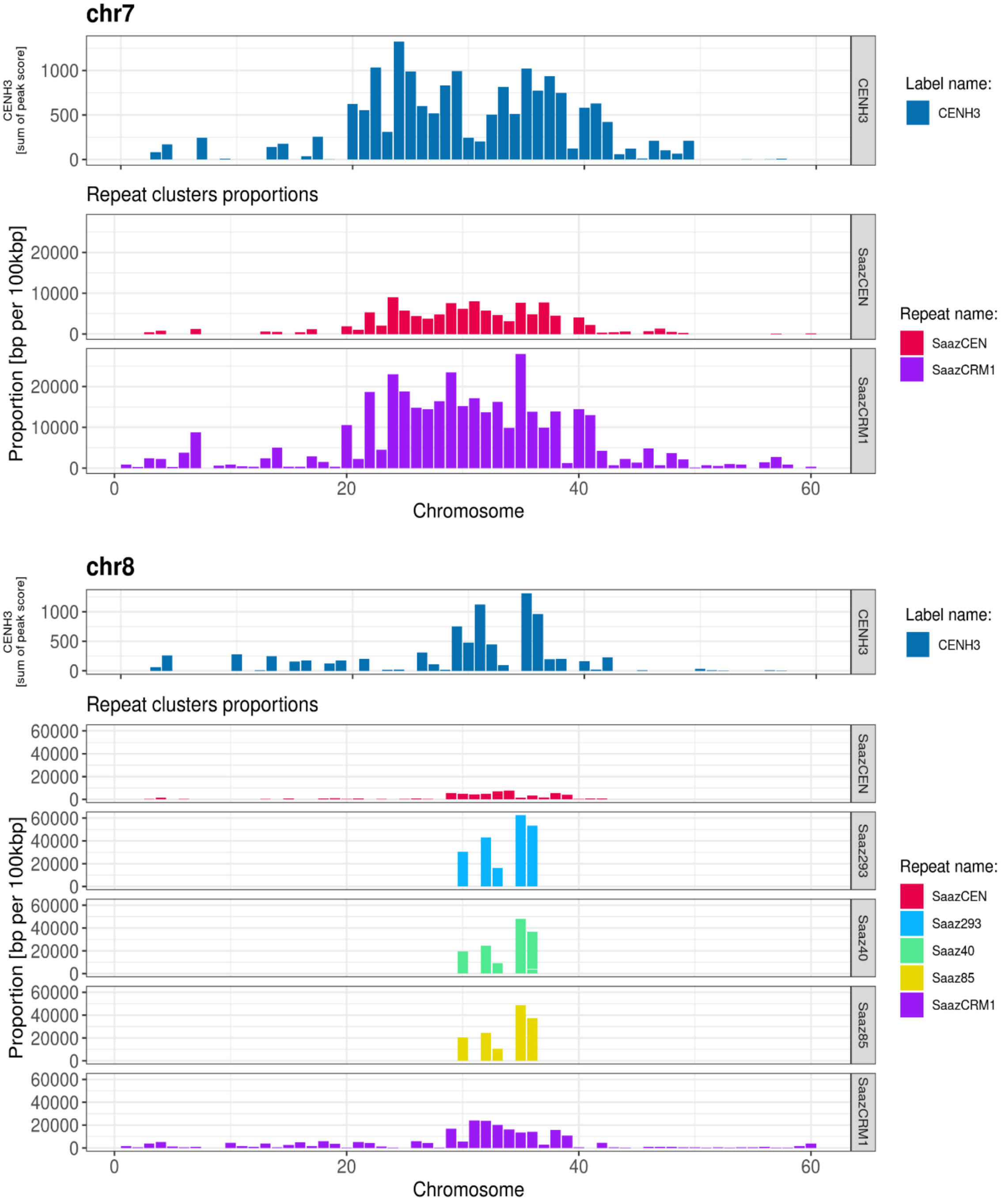

Fig. S5_ part 5 The distribution of major centromeric repeat arrays for each chromosome in the *Humulus lupulus* genome. The first line represents the HlCENH3 binding region domain. The second line shows the distribution of major centromere-specific repeats across chromosomes. Main centromeric repeat SaazCEN and centromere-specific retrotransposon SaazCRM1, were found to be shared among all chromosomes. Note the specific distribution of HuluTR120 and low proportion of SaazCEN and SaazCRM1 on Y chromosome.

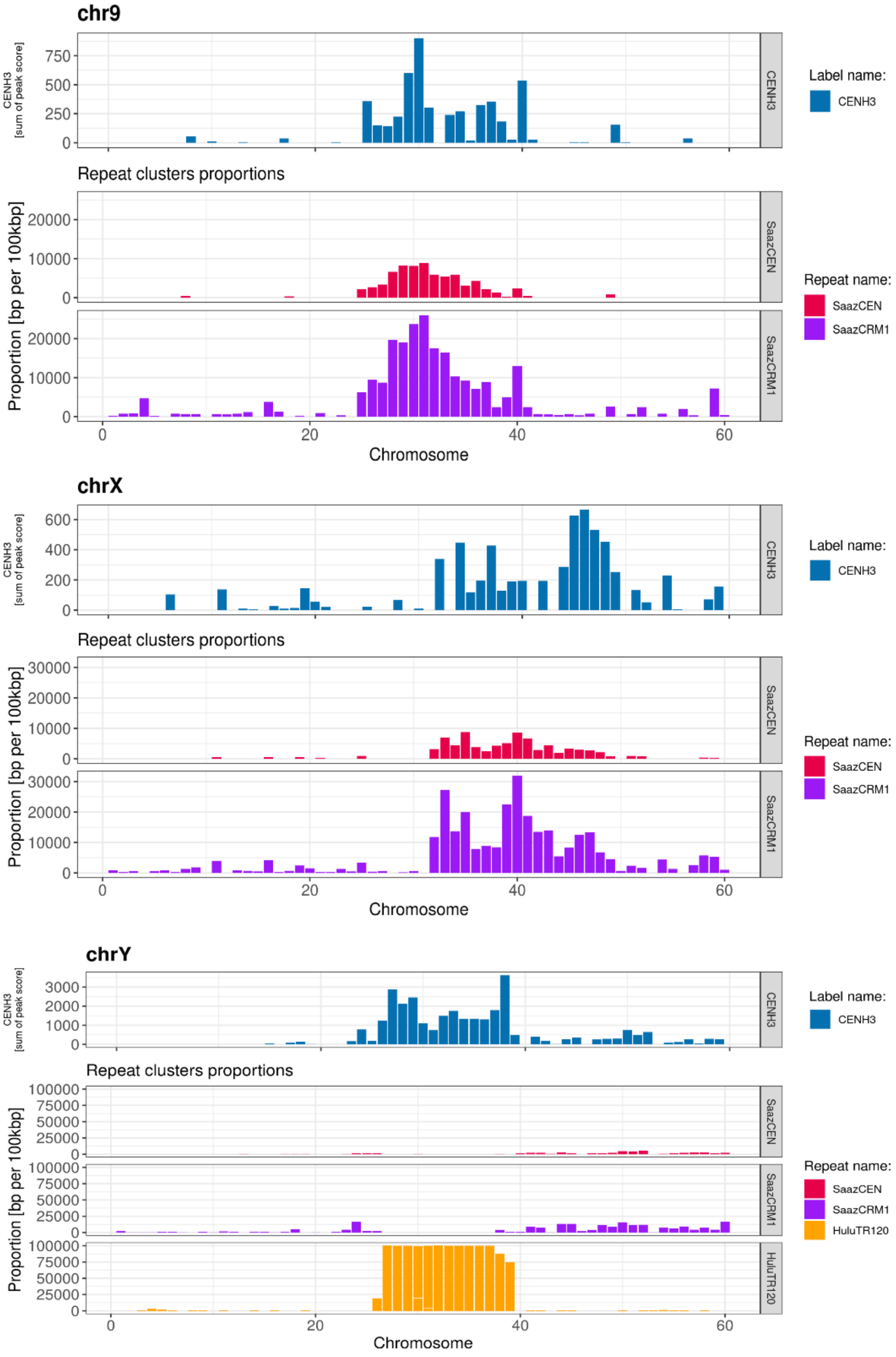

Fig. S6 Distribution of major centromeric repeats on metaphase chromosomes of *Humulus lupulus* Saaz female. Subtelomeric repeat HSR1 (cyan) was used to distinguish two X chromosomes (pericentromeric localization on p-arm and subtelomeric localization on q-arm). (a) Localization of SaazCEN (magenta) and HSR1 (cyan). (b) Simultaneous localization of SaazCEN (magenta), SaazCRM1 (green), and HSR1 (cyan); (c) SaazCEN (magenta), Saaz293 (green), and HSR1 (cyan); (d) HuluTR120 (magenta), Saaz293 (green), and HSR1 (cyan); (e) SaazCEN (magenta), HuluTR120 (green), and HSR1 (cyan). Note the pericentromeric localization of HuluTR120 on chromosome pair 2 and chromosome 3 (only one chromosome from pair). (f) Localization of SaazCEN (magenta), Saaz85 (green), and HSR1 (cyan) satellites. Chromosomes were counterstained with DAPI. Scale bar = 10 µm.

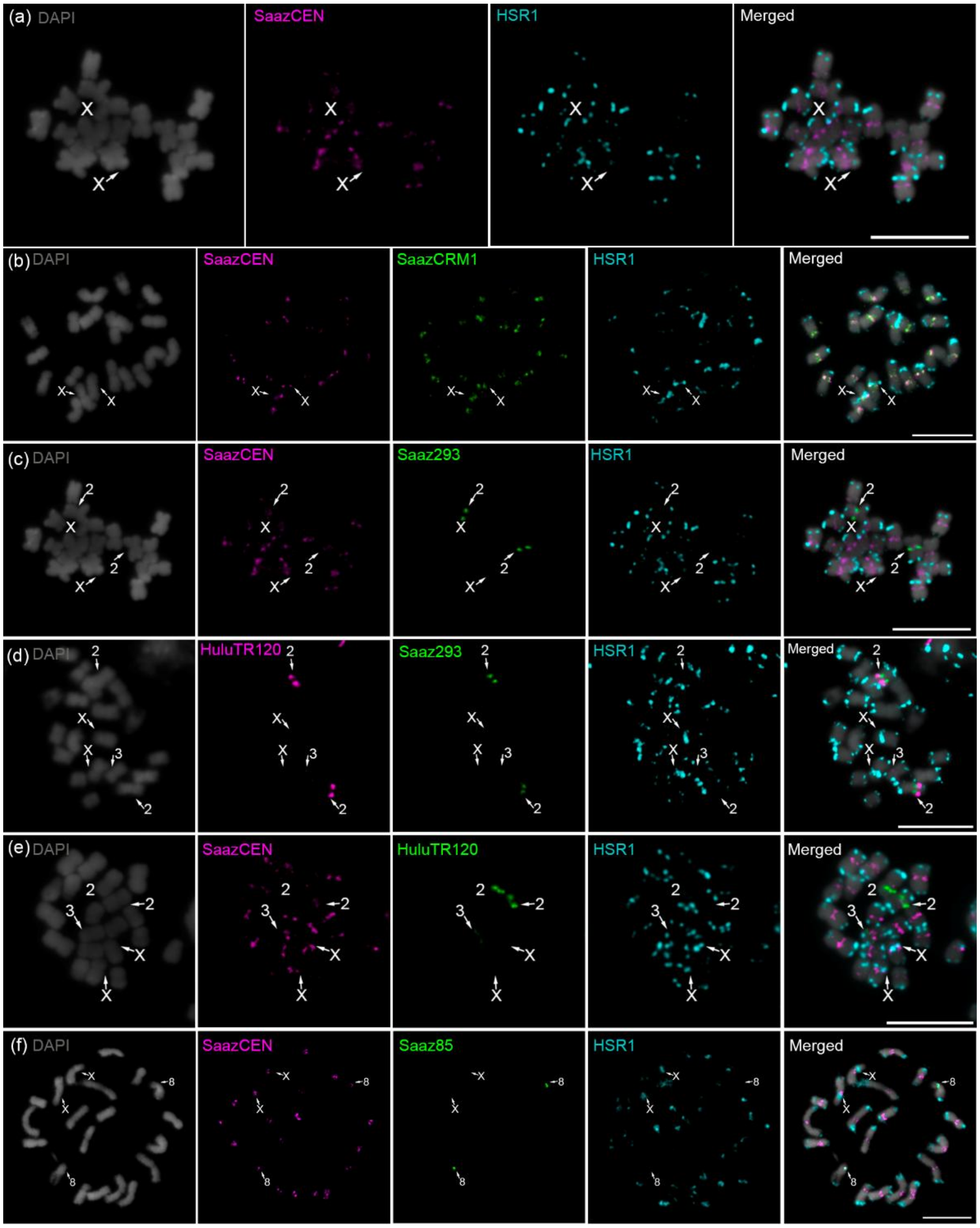

Fig. S7 text below.

Fig. S7 Male karyotype of *Humulus lupulus*, 2n = 20, XY. (a) The centromeric repeat SaazCEN (red; newly identified in this study) and previously characterized subtelomeric repeat HSR1 (cyan) were used to distinguish the p- and q-arms, and X and Y chromosomes. The HSR1 satellite is localized in the subtelomeric region of all autosomes, except for the p-arm of chromosome 8 and the X chromosome (HSR1 signal is in pericentromeric region). In the Y chromosome, HSR1 is present only on the p-arm in subtelomeric region. Mitotic chromosomes were counterstained with DAPI. Scale bar = 10 µm. (b) The distribution and proportion of centromeric repeats across all chromosomes is shown in 100 kb window. Repeats are color-coded according to the provided legend.

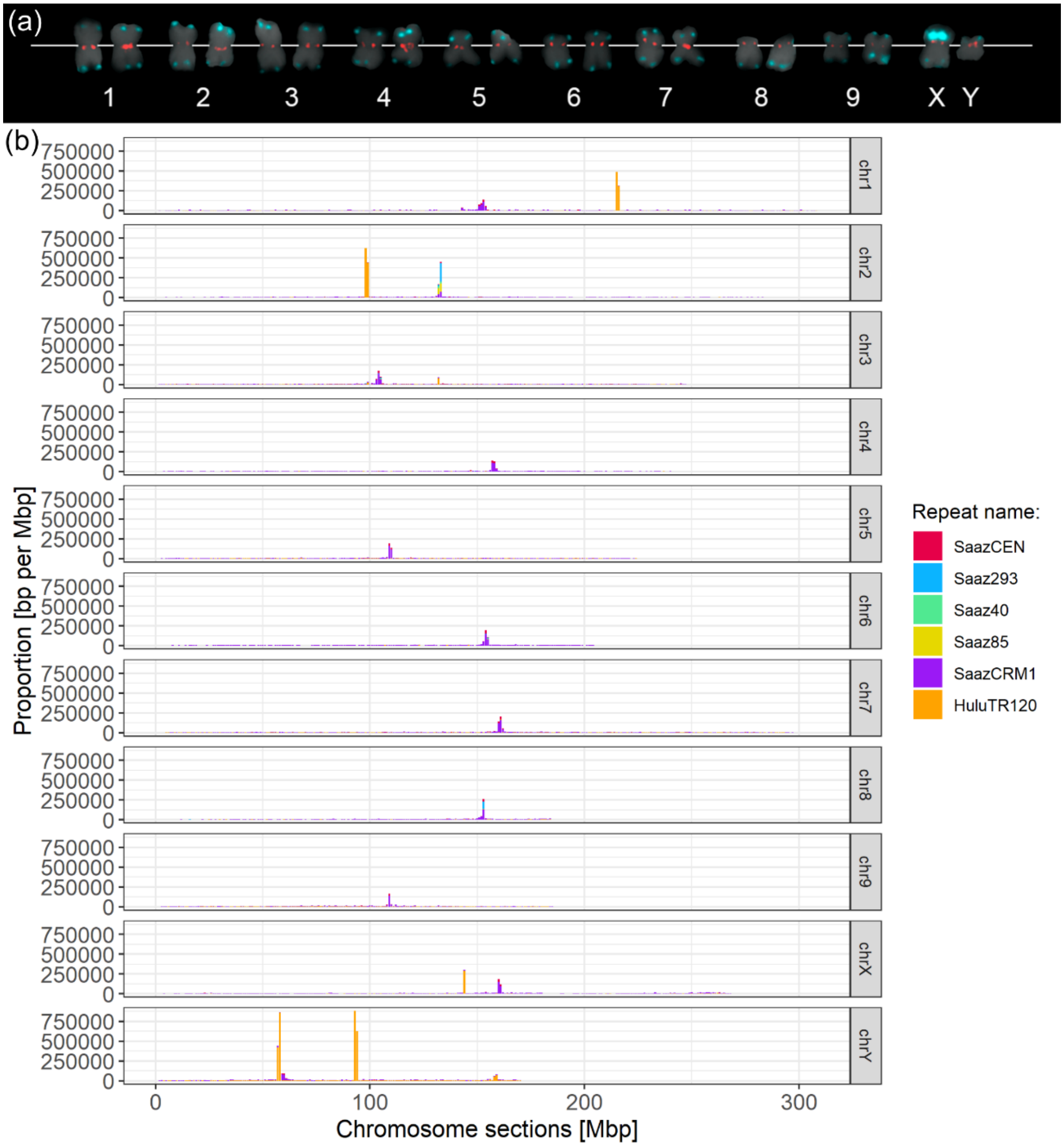

Fig. S8 Detailed analysis of centromere composition in three different *Humulus lupulus* male accessions. The chromosomal composition of three male accessions (a) 15246, (b) 15249, and (c) 15276 was analyzed using three satellites: the pericentromeric HuluTR120 (magenta), the chromosome 2-specific centromeric repeat Saaz293 (green), and subtelomeric repeat HSR1 (cyan). These repeats distinguish chromosomes 2, 3, and Y. In accession 15246 (a), HuluT120 was detected in only one chromosome of autosomal pair 3 (odd number of loci). In contrast, accessions 15249 (b) and 15276 (c) exhibited an even number of HuluTR120 loci. The presence of a single HuluTR120 locus on chromosome 3 in 15246 suggests a chromosome heterozygosity and hybrid origin for chromosome 3 of unknown ancestry. Notably, all studied accessions displayed two HuluTR120 signals on chromosome Y. Chromosomes were counterstained with DAPI. Scale bar = 10 μm.

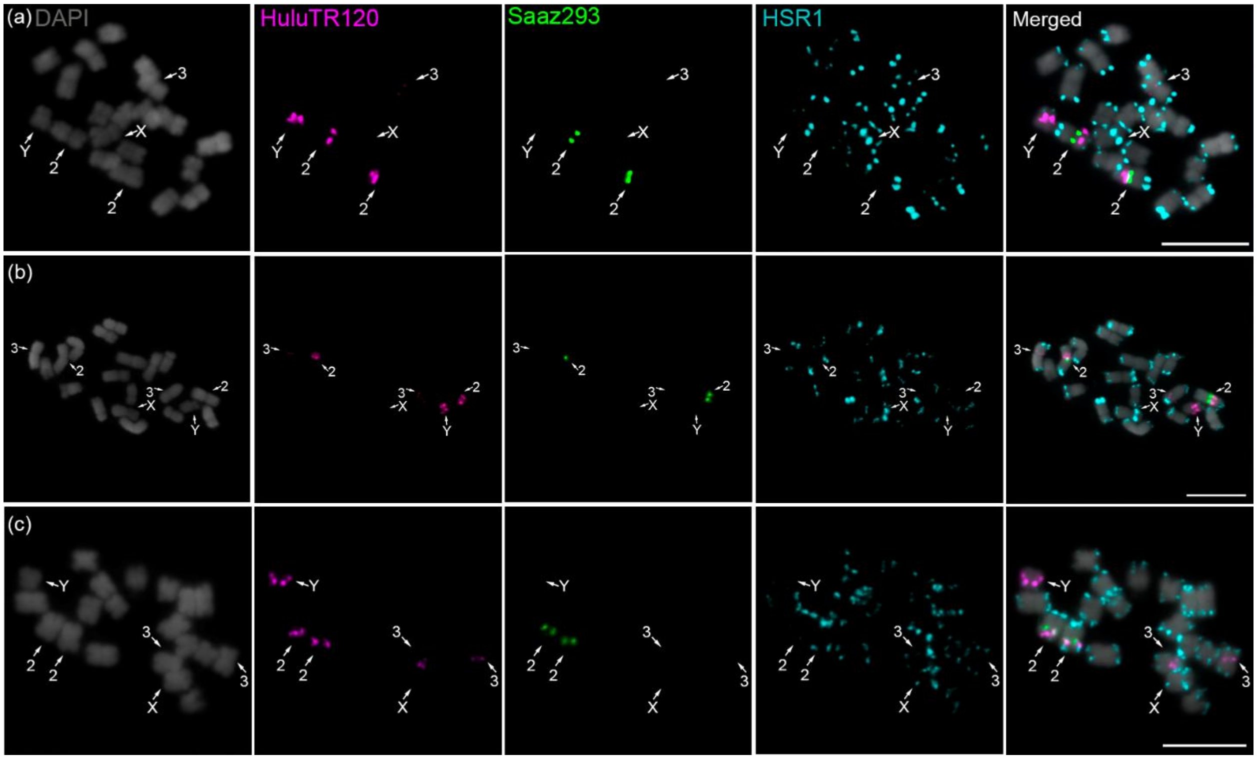

Fig. S9 Localization of the centromeric satellite Saaz40 and 45S rDNA on chromosome 8 in *Humulus lupulus*. The centromeric satellites Saaz40 (magenta) and 45S rDNA (green) were localized on chromosome 8 in (a) male and (b) female *Humulus lupulus*. The Saaz40 satellite exhibits a metaphase chromosome pattern similar to that of Saaz85 satellite (Fig. 1g). Mitotic chromosomes were counterstained with DAPI. Scale bar = 10 µm.

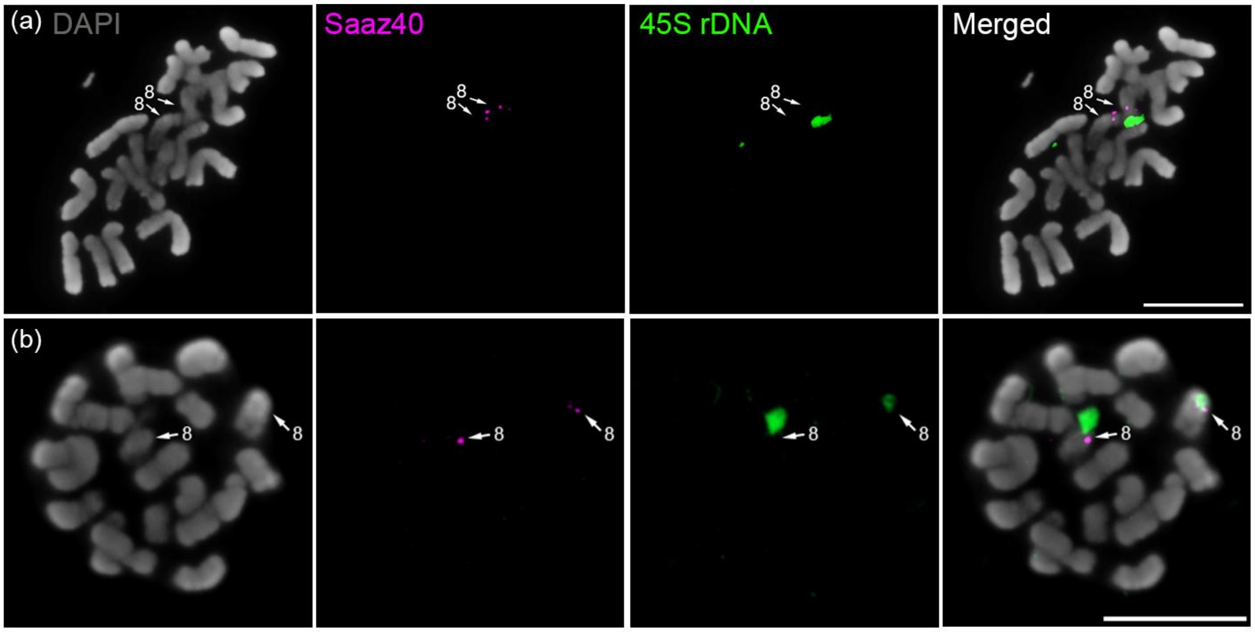

Fig. S10 Localization of the centromeric satellite Saaz85 and 45S rDNA on chromosome 8 in *Humulus lupulus*. The centromeric satellites Saaz85 (magenta) and 45S rDNA (green) were localized on chromosome 8 in (a) male and (b) female *Humulus lupulus*. Subtelomeric repeat HSR1 (cyan) was used to distinguish X and Y chromosomes. Mitotic chromosomes were counterstained with DAPI. Scale bar = 10 μm.

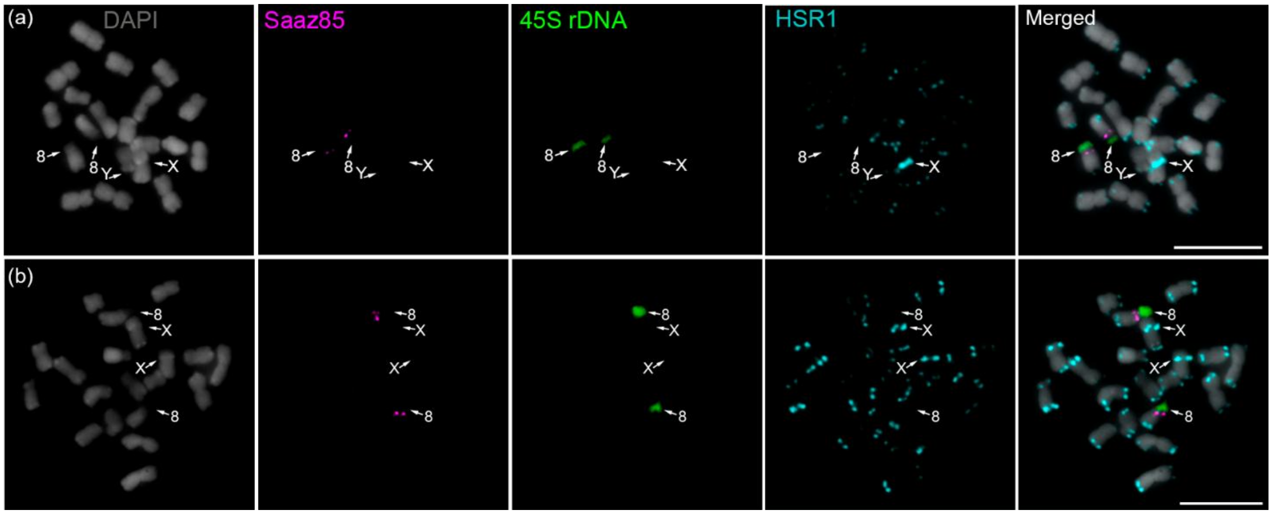

Fig. S11 Chromosome pairing and organization during diakinesis in *Humulus lupulus* Lib male. (a) The subtelomeric satellite HSR1 (magenta) marks the position of the pseudoautosomal region (PAR) and indicates the pairing between X and Y chromosomes. (b) HuluTR120 satellite (green) specifically differentiates the Y chromosome. Meiotic chromosomes were counterstained with DAPI. Scale bar = 10 μm.

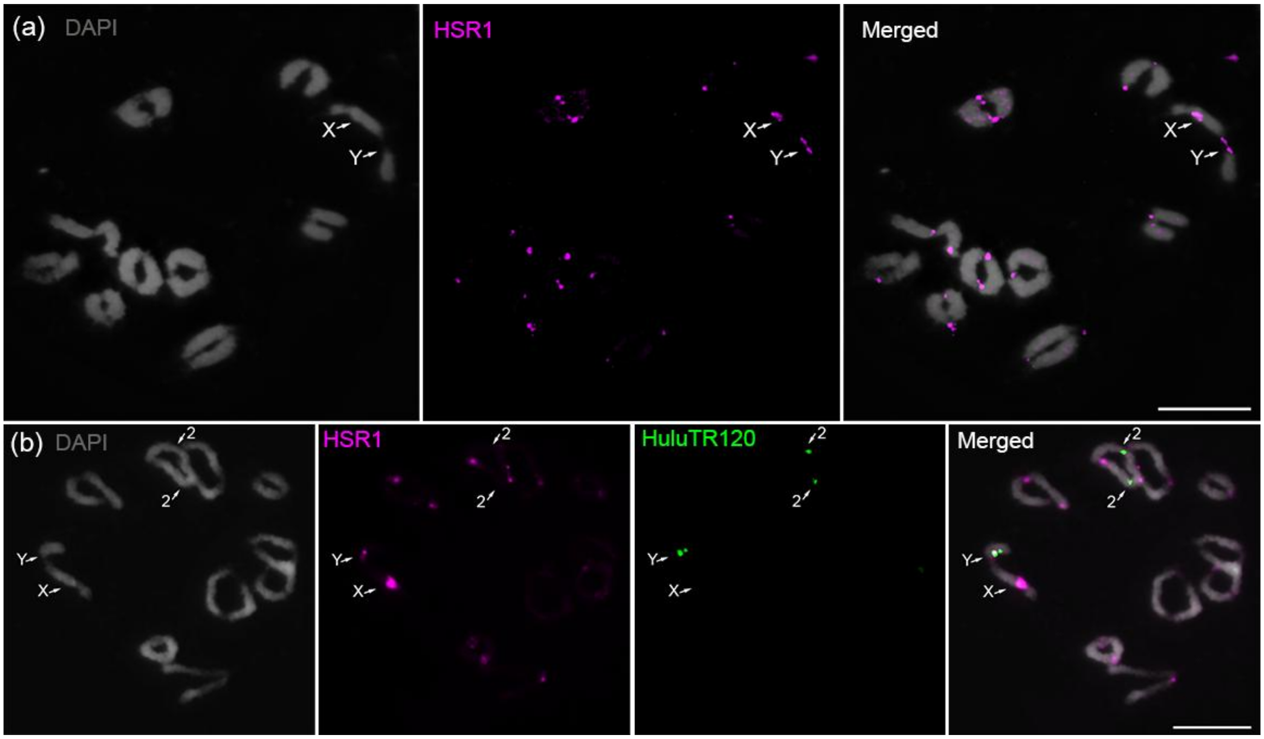

Fig. S12 Distribution of major centromeric repeats on metaphase chromosomes of *Humulus lupulus* Lib male. Subtelomeric repeat HSR1 (cyan) was used to distinguish X (pericentromeric distribution on p-arm) and Y chromosomes (only p-arm). (a) Simultaneous localization of SaazCEN (magenta) and HSR1 (cyan); (b) SaazCEN (magenta), SaazCRM1 (green), and HSR1 (cyan); (c) SaazCEN (magenta), Saaz293 (green), and HSR1 (cyan); (d) HuluTR120 (magenta), Saaz293 (green), and HSR1 (cyan); (e) SaazCEN (magenta), HuluTR120 (green), and HSR1 (cyan); (f) SaazCEN (magenta), Saaz85 (green), and HSR1 (cyan). Chromosomes were counterstained with DAPI. Scale bar = 10 μm.

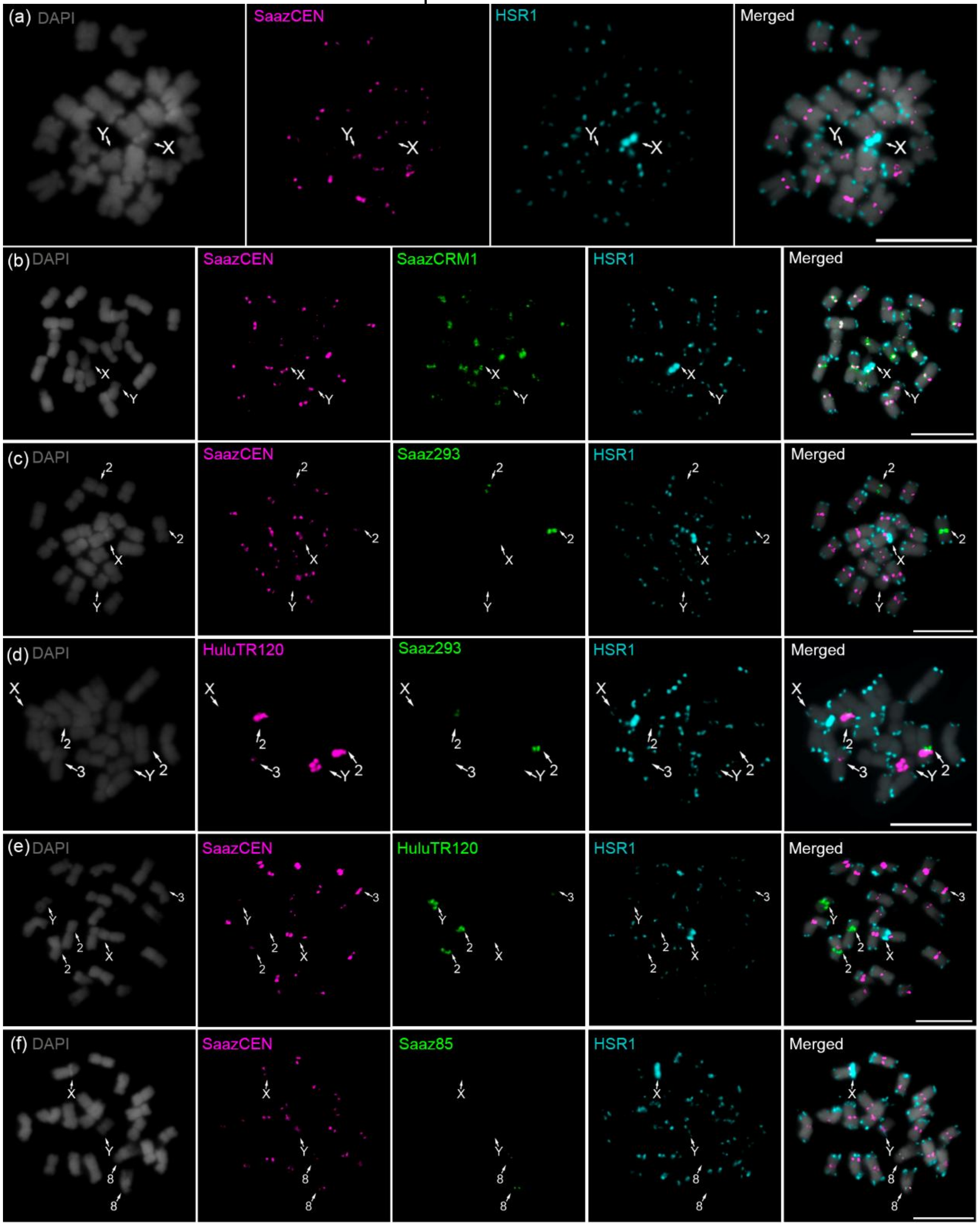

Fig. S13 Dot plot analysis and sequence similarity of three major centromeric satellites, Saaz85, Saaz293, and Saaz40 which are specific for chromosomes 2, 3, 6, and 8 in *Humulus lupulus*. All three satellites exhibit large and conserved regions across all pairwise comparisons.

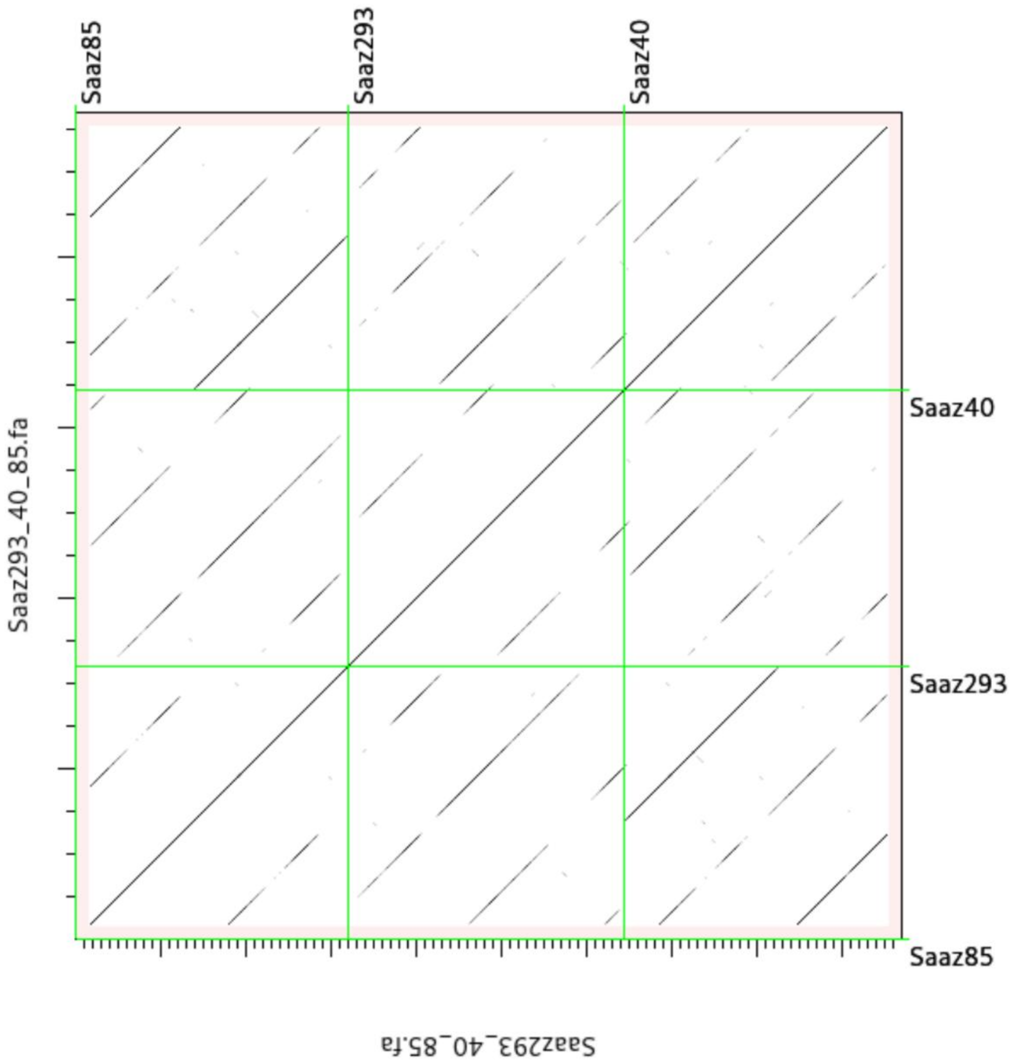

Fig. S14_part 1 The distribution of centromeric repeats in *Humulus lupulus* accessions (Saaz, male "10-12", Cascade, and drHumLupu1). Notably, satellites Saaz85 and Saaz293 are present on chromosomes 2 of Saaz, "10-12" and drHumLupu1, while in Cascade the same chromosome display low proportion of both satellites.

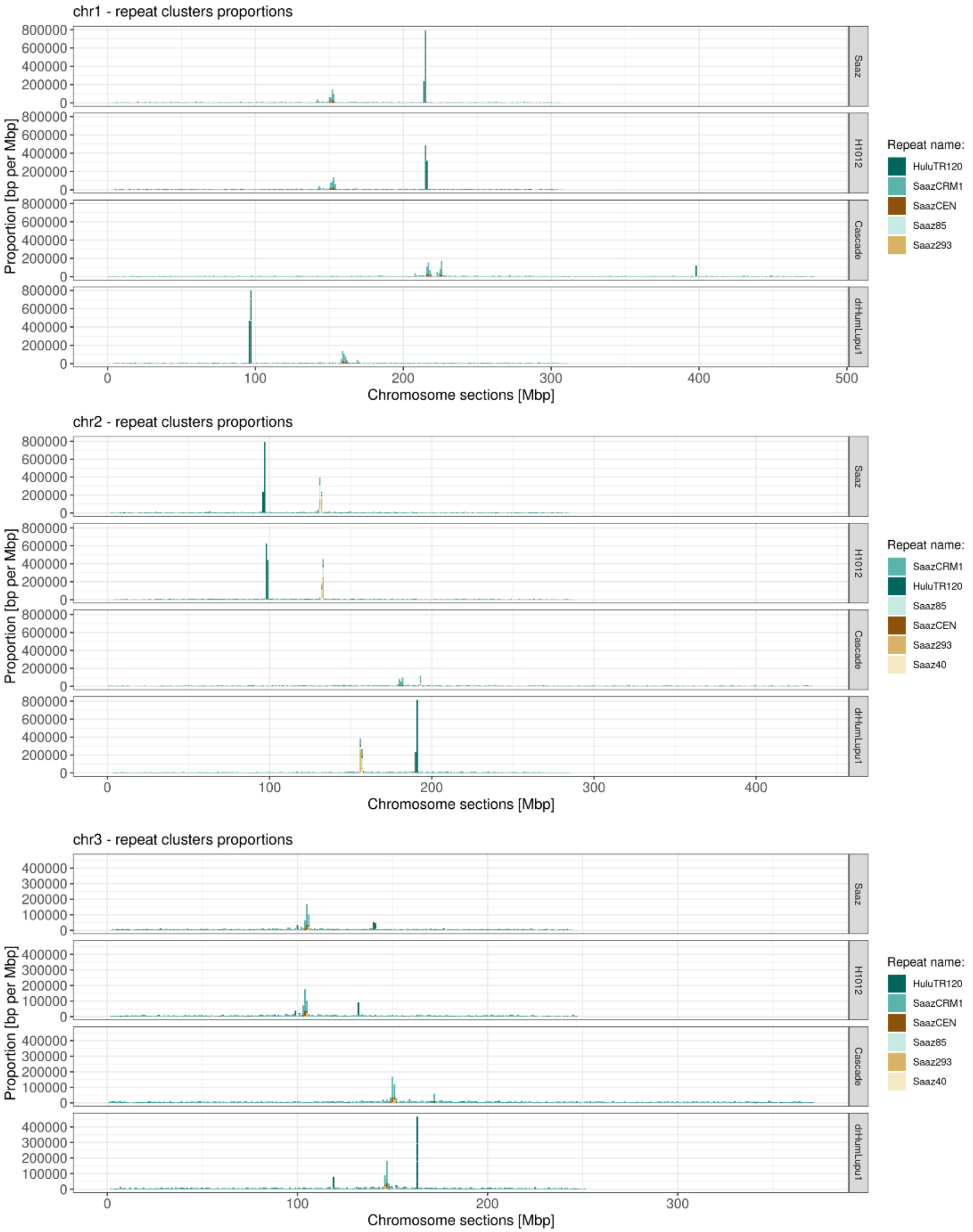

Fig. S14_part 2 The distribution of centromeric repeats in *Humulus lupulus* accessions (Saaz, male "10-12", Cascade, and drHumLupu1). Notably, satellites Saaz85, Saaz40, and Saaz293 are present on chromosomes 6 of Saaz, "10-12" and drHumLupu1, while in Cascade the same chromosome display low proportion of all three satellites.

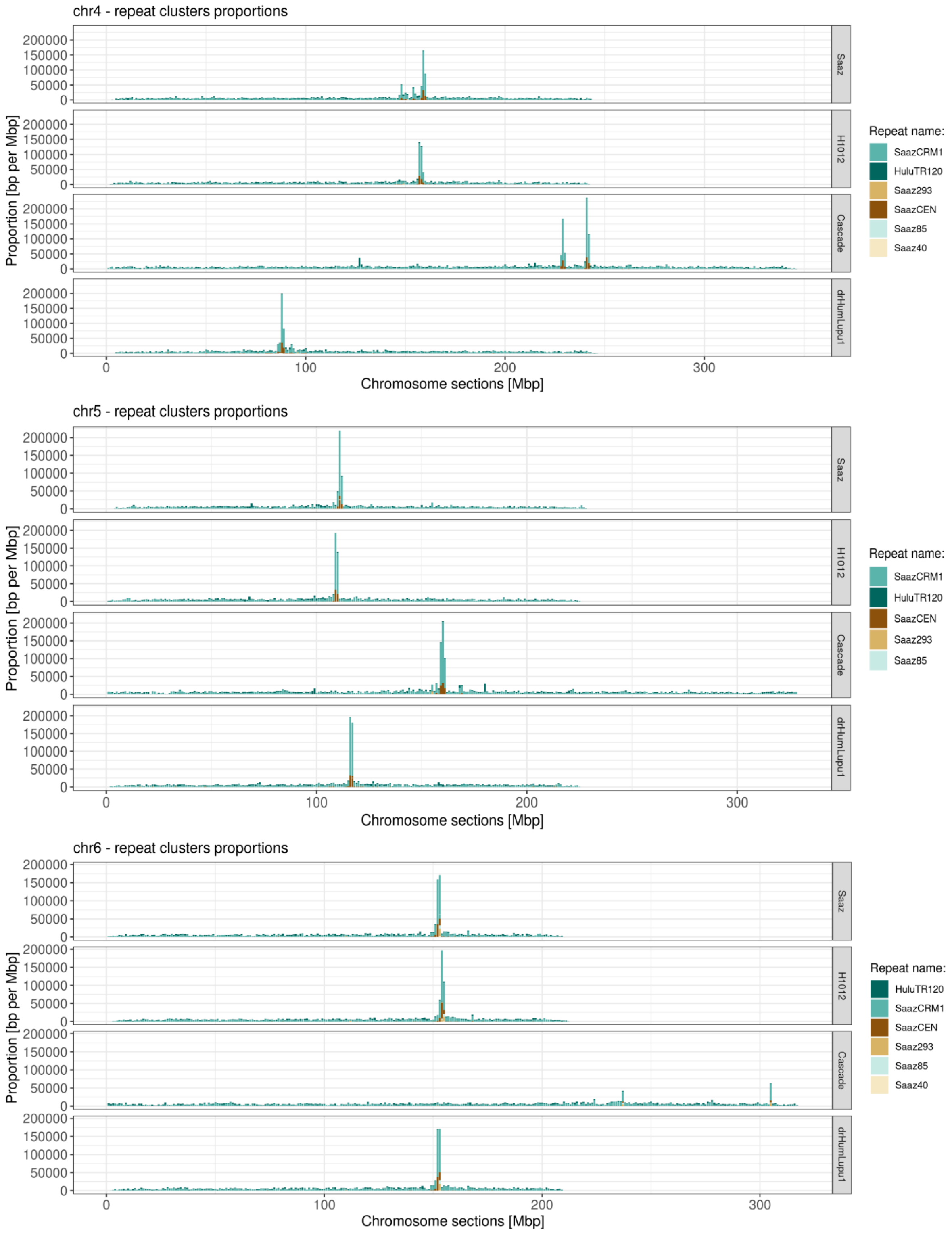

Fig. S14_part 3 The distribution of centromeric repeats in *Humulus lupulus* accessions (Saaz, male "10-12", Cascade, and drHumLupu1). Notably, satellites Saaz85, Saaz40, and Saaz293 are present on chromosomes 8 of Saaz, "10-12" and drHumLupu1, while in Cascade the same chromosome display low proportion of all three satellites.

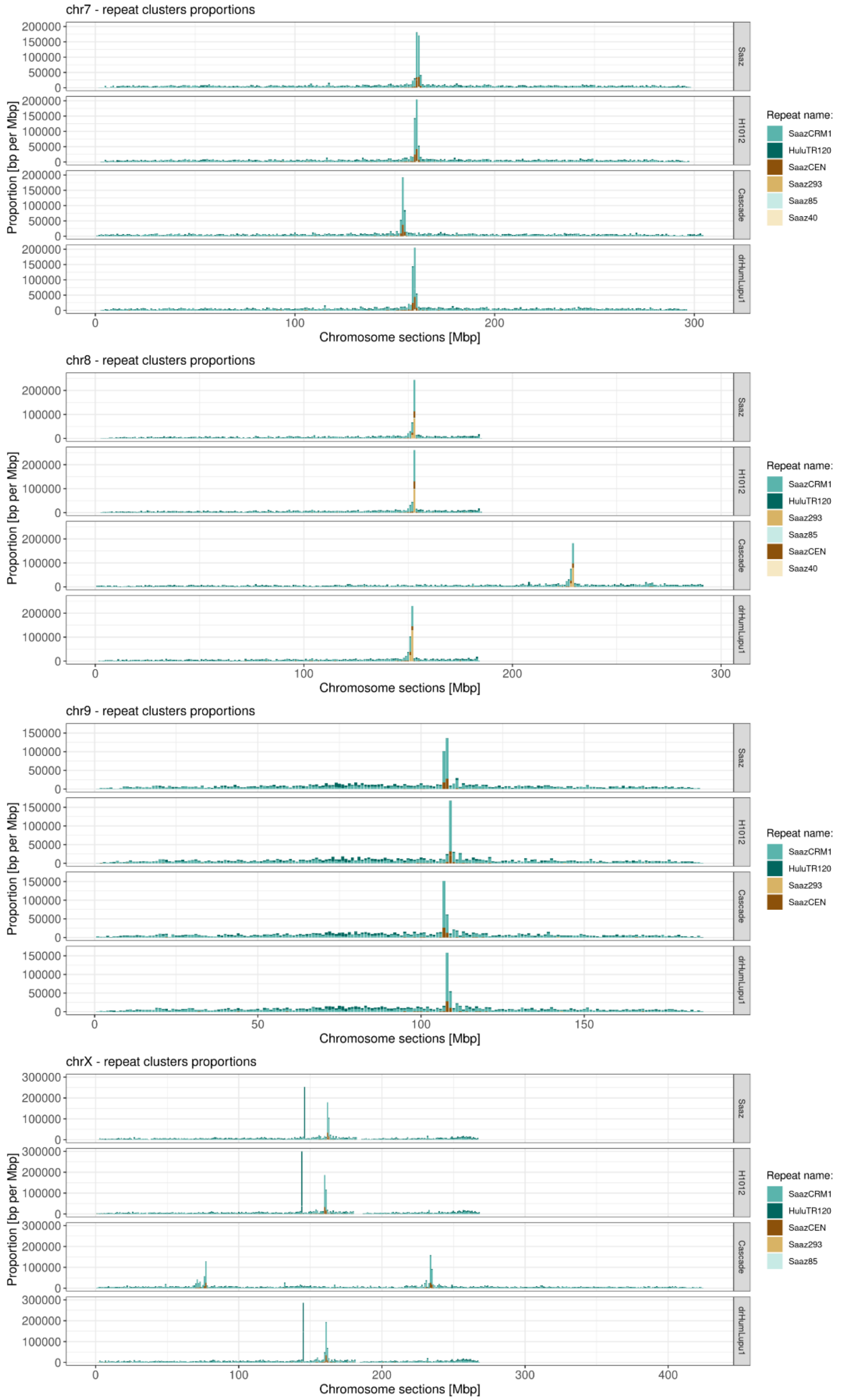

Fig. S15 Sequence analysis of the SaazCEN repeat localized within LTR regions of *Humulus lupulus* CRMs. (a) Dot plot of the SaazCEN sequence reveals a tandem array of 39 bp subunits (highlighted in orange). (b) Coverage analysis of CENH3 ChIP-seq reads shows peak enrichment within the region corresponding to the 39 bp repeat units (highlighted, spanning positions 189–263 bp). (c) Frequency distribution of the 39 bp subunits in LTRs of CRMs inserted within the 6 Mb centromeric region of each chromosome. The highest average number of copies of 39 SaazCEN subunits is between 3 and 4 per one LTR.

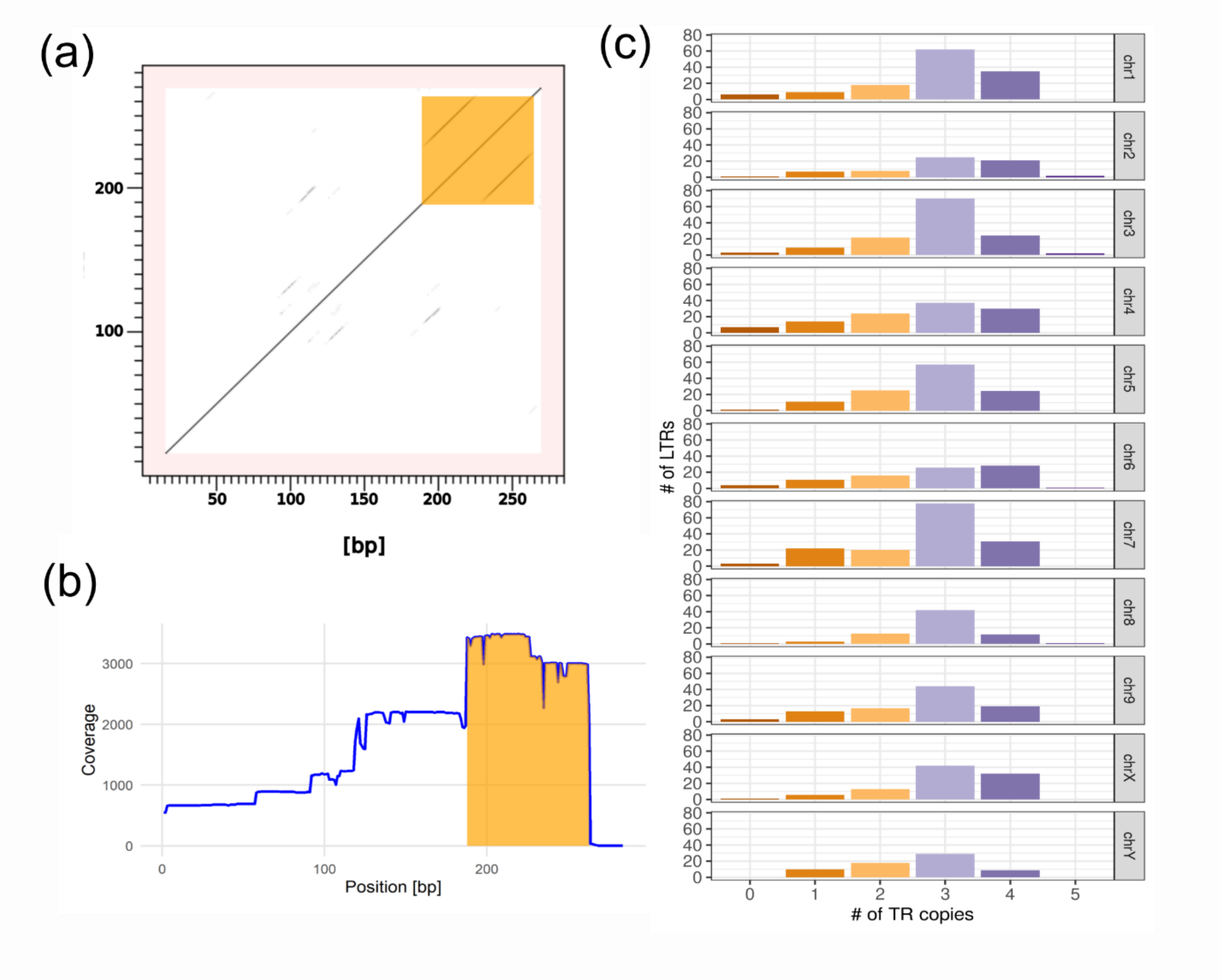

Fig. S16 LTR retrotransposons composition of all centromeres of *Humulus lupulus* genome. (a) Repeat composition of Ty1/*Copia* families. Ale (red) and Angela (orange) clades are presented in the centromeres of all chromosomes, together accounting for the majority of Ty1/*Copia* insertions. Other clades, including Ikeros, SIRE, and TAR, are represented unevenly. The Ikeros clade (light yellow) is specific to the centromeres of chromosomes 2, 3, and X. (b) Repeat composition of Ty3/*Gypsy* families. Tekay and CRM clades are the most abundant transposable elements (TEs), represented in all chromosomes in similar proportions, with the exception of chromosome 3. Retand and Athila constitute a minor fraction of TEs across all chromosomes.

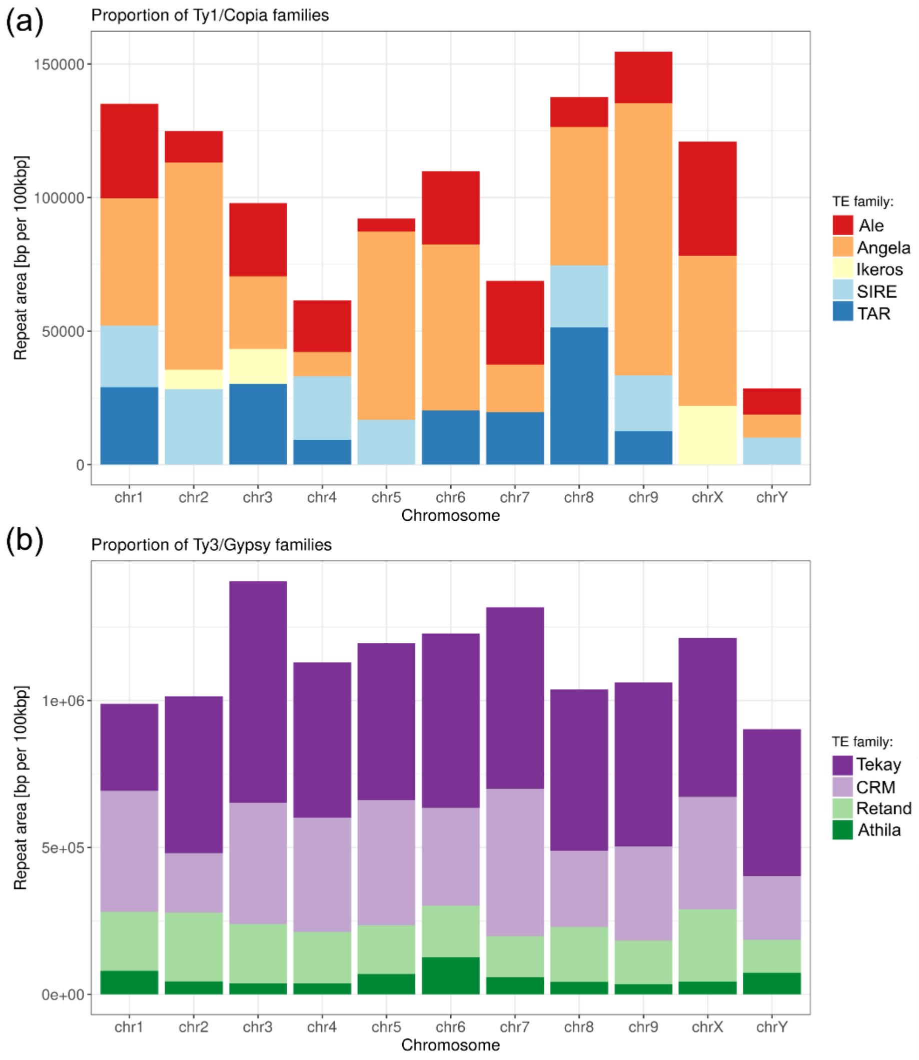

Fig. S17_part 1 The distribution of seven transposable element clades (Athila, CRM, Galadriel, Ogre, Reina, Retand, and Tekay) of Ty3/*Gypsy* family in *Humulus lupulus* accessions (Saaz, male "10-12", Cascade, and drHumLupu1). Notably, CRM sequences (light brown) exhibit significant enrichment at the centromeres of all chromosomes across available accessions.

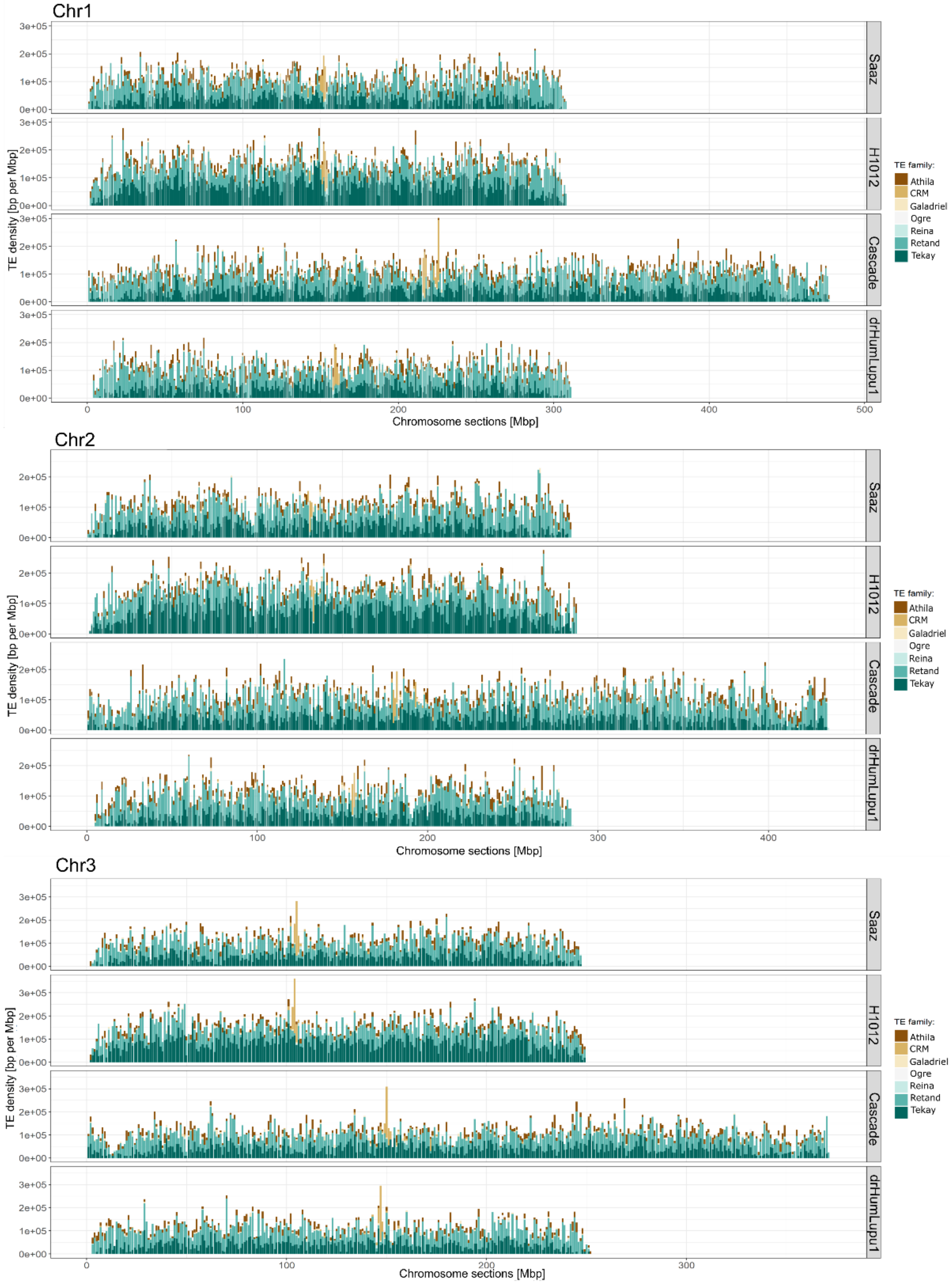

New Phytologist

Fig. S17_part 2 The distribution of seven transposable element clades (Athila, CRM, Galadriel, Ogre, Reina, Retand, and Tekay) of Ty3/*Gypsy* family in *Humulus lupulus* accessions (Saaz, male "10-12", Cascade, and drHumLupu1). Notably, CRM sequences (light brown) exhibit significant enrichment at the centromeres of all chromosomes across available accessions.

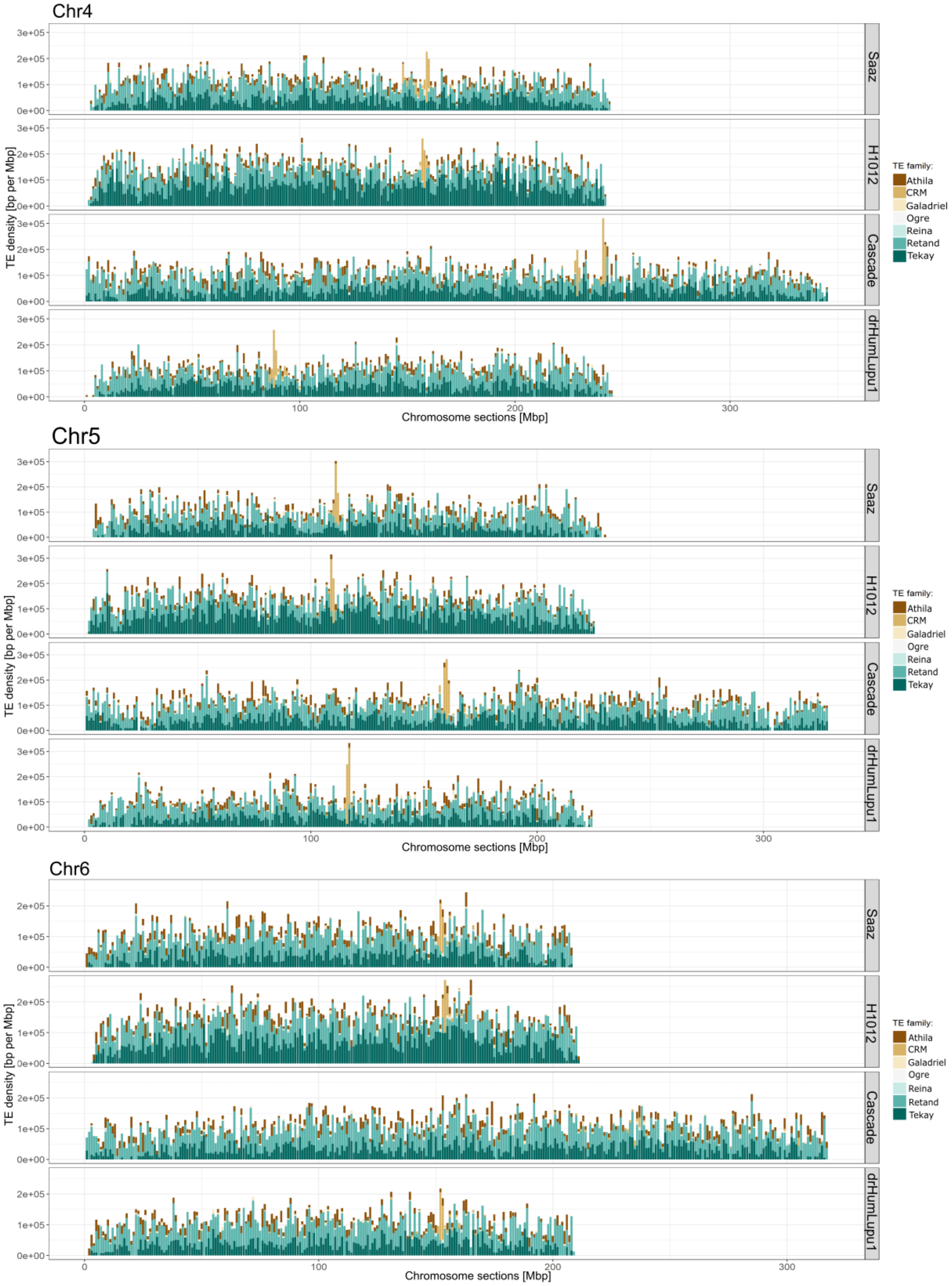

Fig. S17_part 3 The distribution of seven transposable element clades (Athila, CRM, Galadriel, Ogre, Reina, Retand, and Tekay) of Ty3/*Gypsy* family in *Humulus lupulus* accessions (Saaz, male "10-12", Cascade, and drHumLupu1). Notably, CRM sequences (light brown) exhibit significant enrichment at the centromeres of all chromosomes across available accessions.

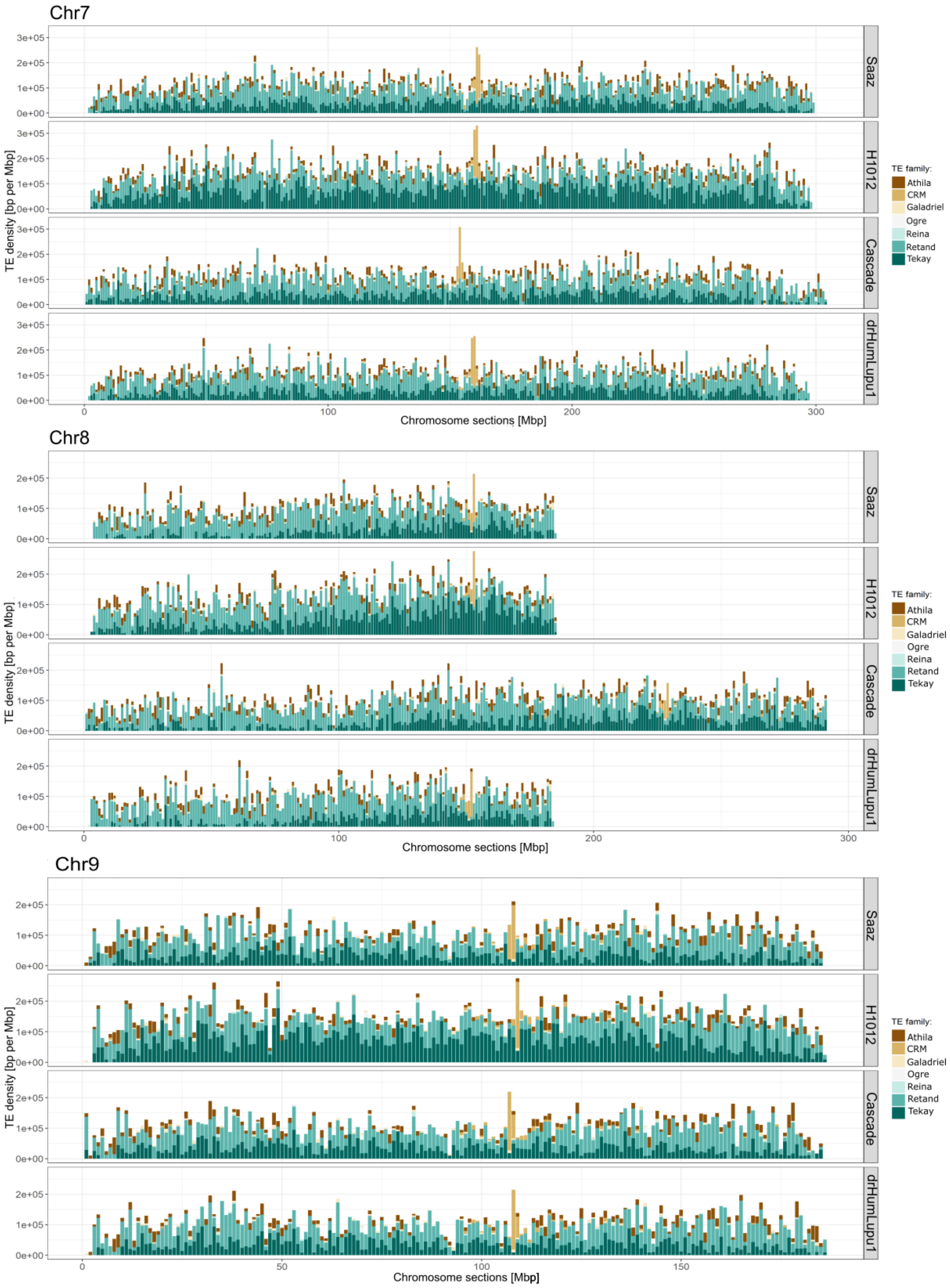

Fig. S17_part 4 The distribution of seven transposable element clades (Athila, CRM, Galadriel, Ogre, Reina, Retand, and Tekay) of Ty3/*Gypsy* family in *Humulus lupulus* accessions (Saaz, male "10-12", Cascade, and drHumLupu1). Notably, CRM sequences (light brown) exhibit significant enrichment at the centromeres of all chromosomes across available accessions.

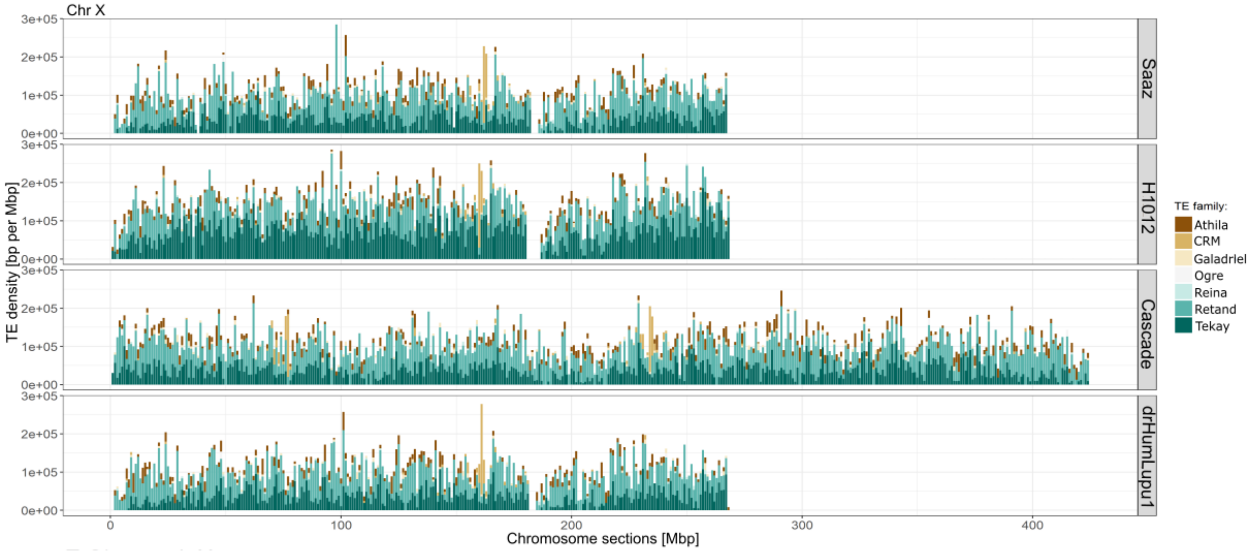

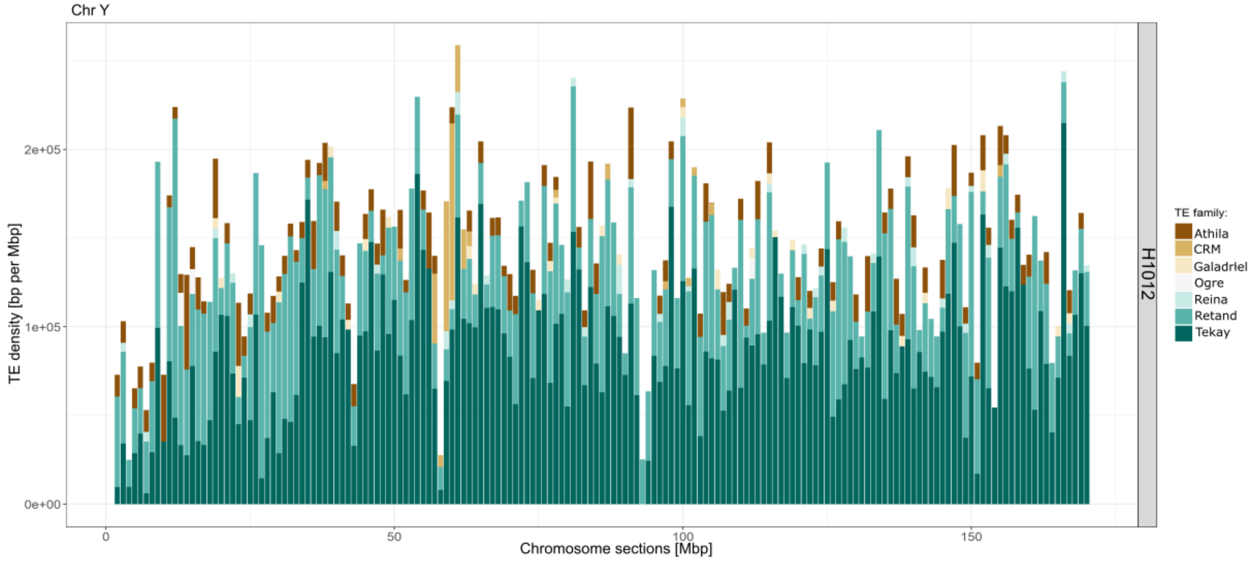

Fig. S18_part 1 Distribution and estimated insertion time of Ty1/*Copia* and Ty3/*Gypsy* LTR retrotransposons families across all chromosomes in the *Humulus lupulus* centromere. The most recent CRM insertions are located within the HICENH3 binding domain. Note that chromosomes 1 and 2 have large, expanded HICENH3-positive regions, with more recent CRM insertions within the main binding domain.

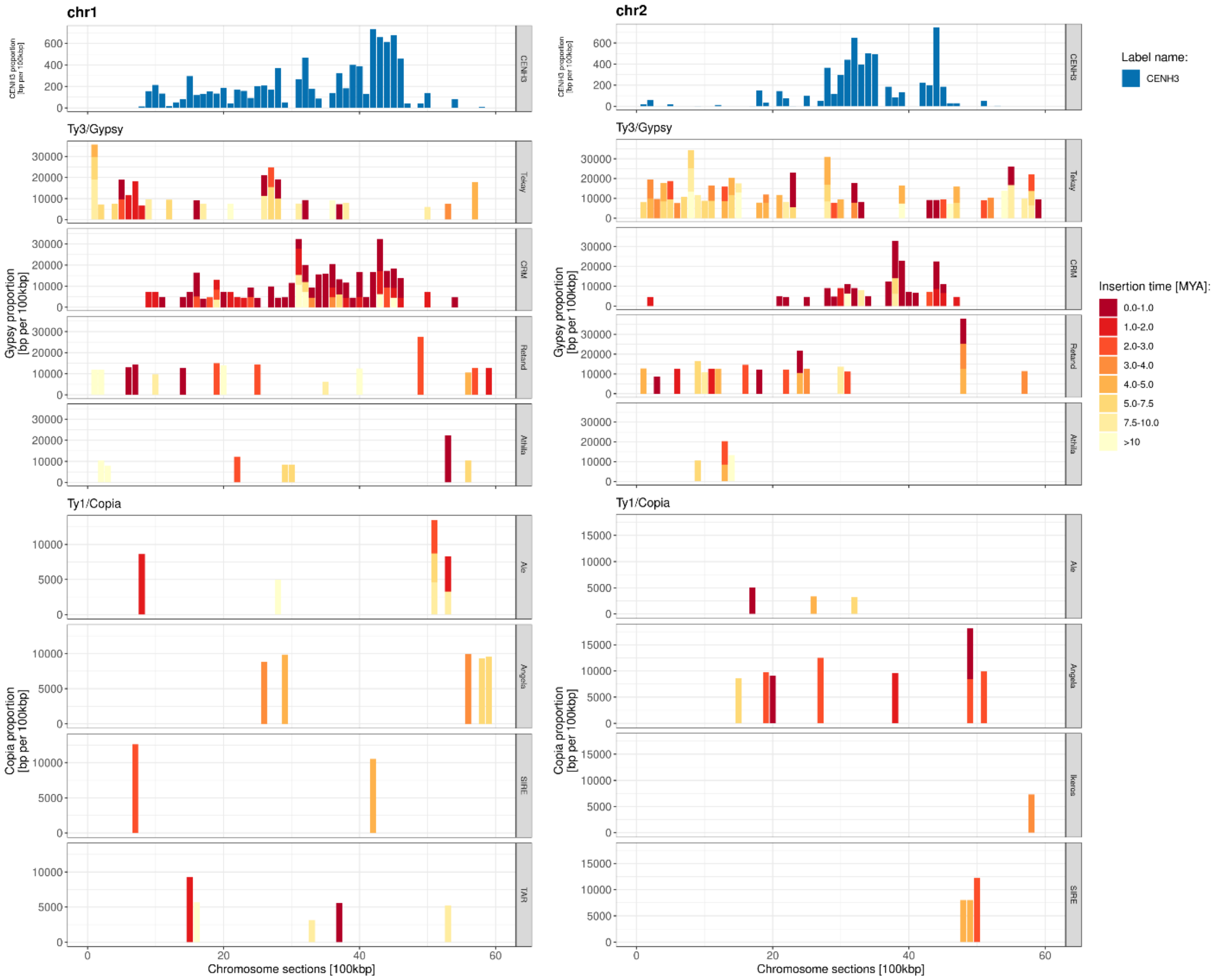

Fig. S18_ part 2 Distribution and estimated insertion time of Ty1/*Copia* and Ty3/*Gypsy* LTR retrotransposons families across all chromosomes in the *Humulus lupulus* centromere. The most recent CRM insertions are located within the HlCENH3 binding domain. Note that chromosome 3 has large, expanded HlCENH3-positive region, with more recent CRM insertions within the main binding domain.

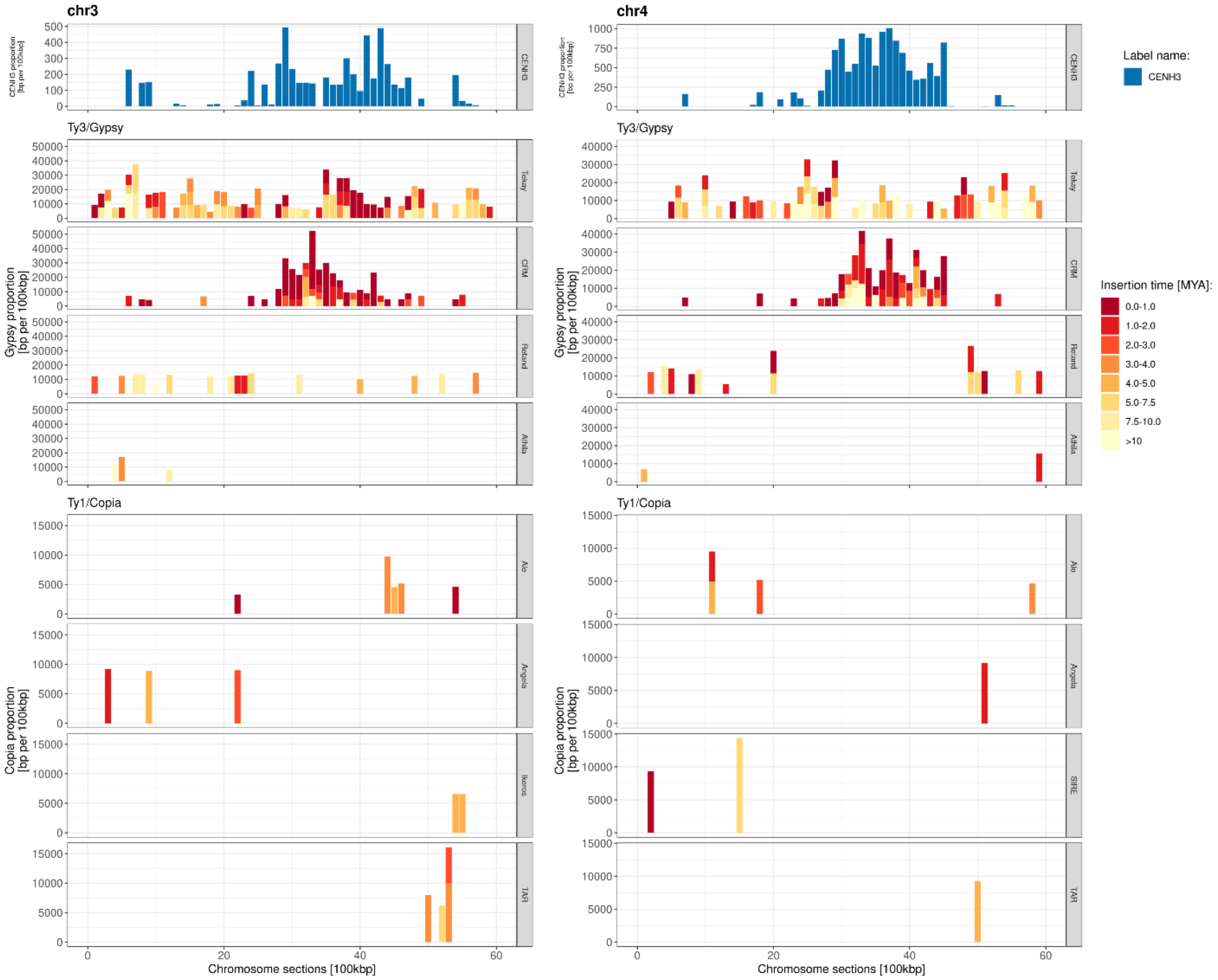

Fig. S18_part 3 Distribution and estimated insertion time of Ty1/*Copia* and Ty3/*Gypsy* LTR retrotransposons families across all chromosomes in the *Humulus lupulus* centromere. The most recent CRM insertions are located within the HlCENH3 binding domain. Note that chromosome 5 has large, expanded HlCENH3-positive region, with more recent CRM insertions within the main binding domain.

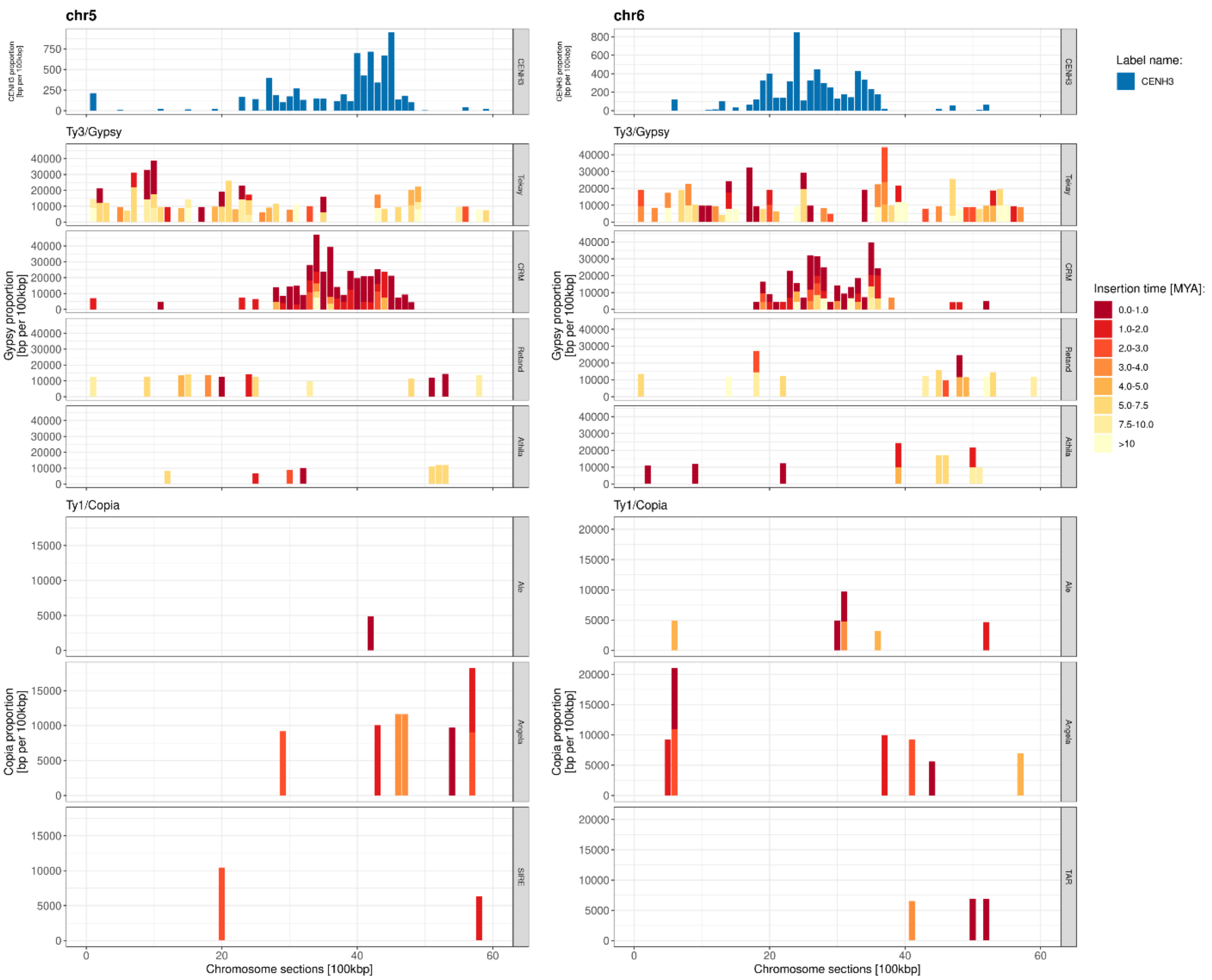

Fig. S18_part 4 Distribution and estimated insertion time of Ty1/*Copia* and Ty3/*Gypsy* LTR retrotransposons families across all chromosomes in the *Humulus lupulus* centromere. The most recent CRM insertions are located within the HlCENH3 binding domain. Note that chromosome 7 has large, expanded HlCENH3-positive region, with more recent CRM insertions within the main binding domain.

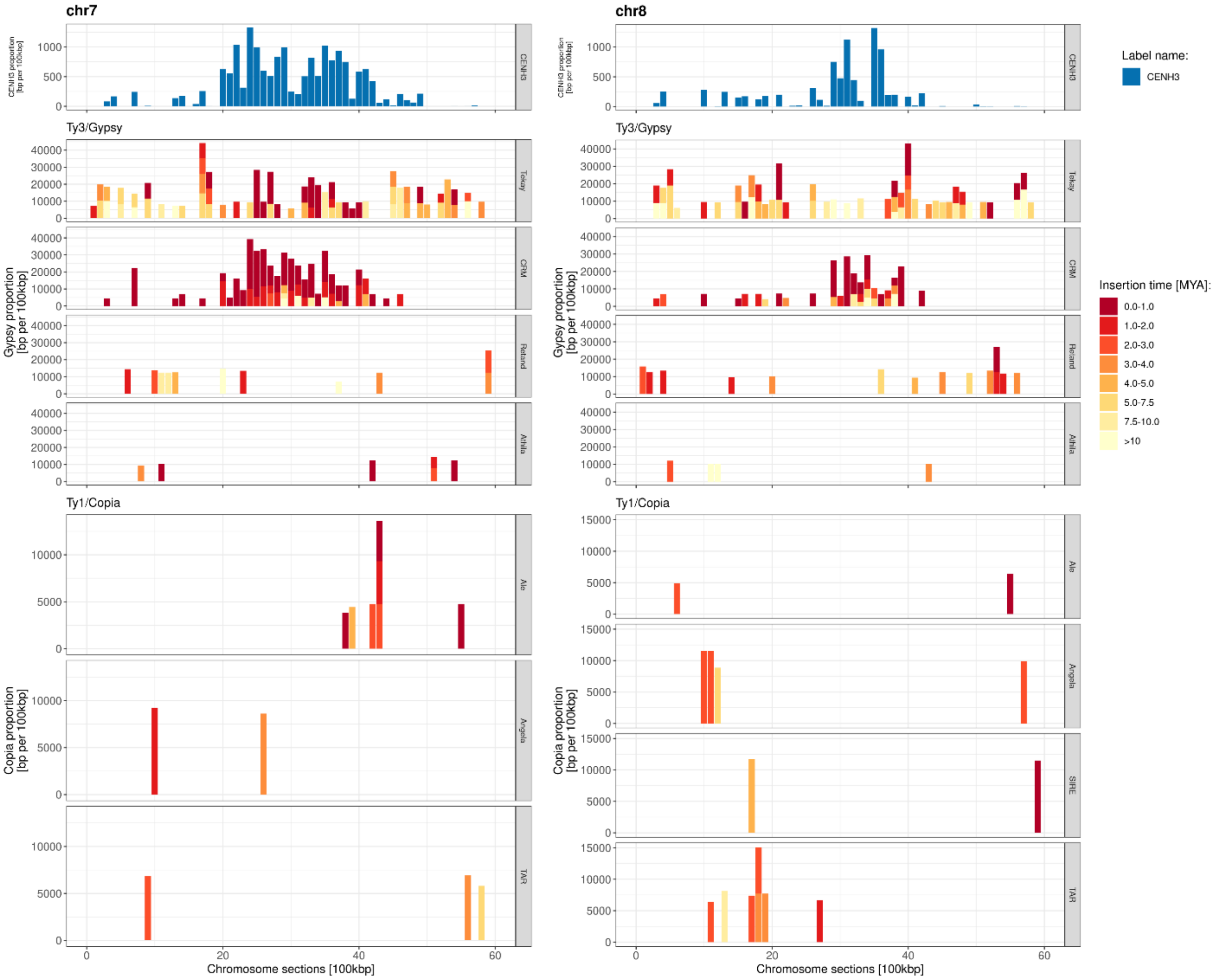

Fig. S18_part 5 Distribution and estimated insertion time of Ty1/*Copia* and Ty3/*Gypsy* LTR retrotransposons families across all chromosomes in the *Humulus lupulus* centromere. The most recent CRM insertions are located within the HICENH3 binding domain.

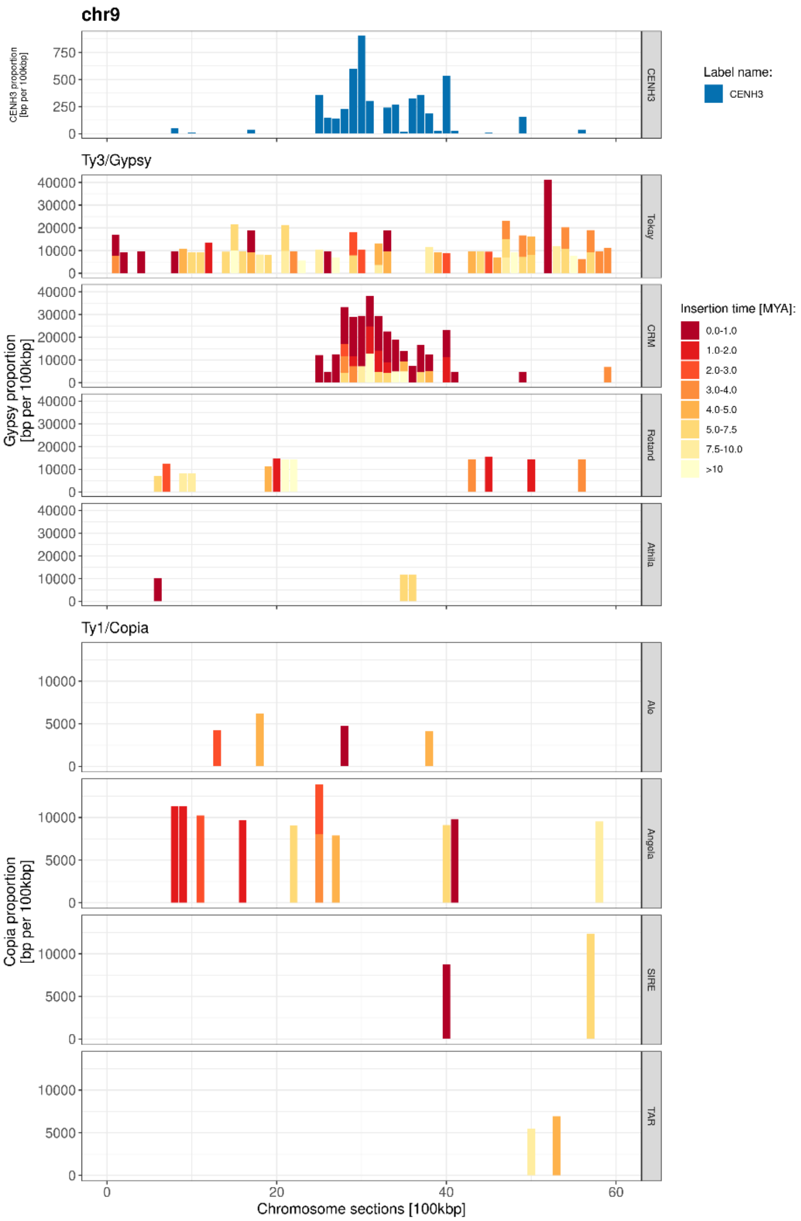

Fig. S18_part 6 Distribution and estimated insertion time of Ty1/*Copia* and Ty3/*Gypsy* LTR retrotransposons families across all chromosomes in the *Humulus lupulus* centromere. The most recent CRM insertions are located within the HlCENH3 binding domain. Note that X chromosome has large, expanded HlCENH3-positive region, with more recent CRM insertions within the main binding domain. Y chromosome has large HlCENH3 region, however the most recent CRM insertions localized in vicinity of HlCENH3 binding domain, discriminating Y centromere boundary.

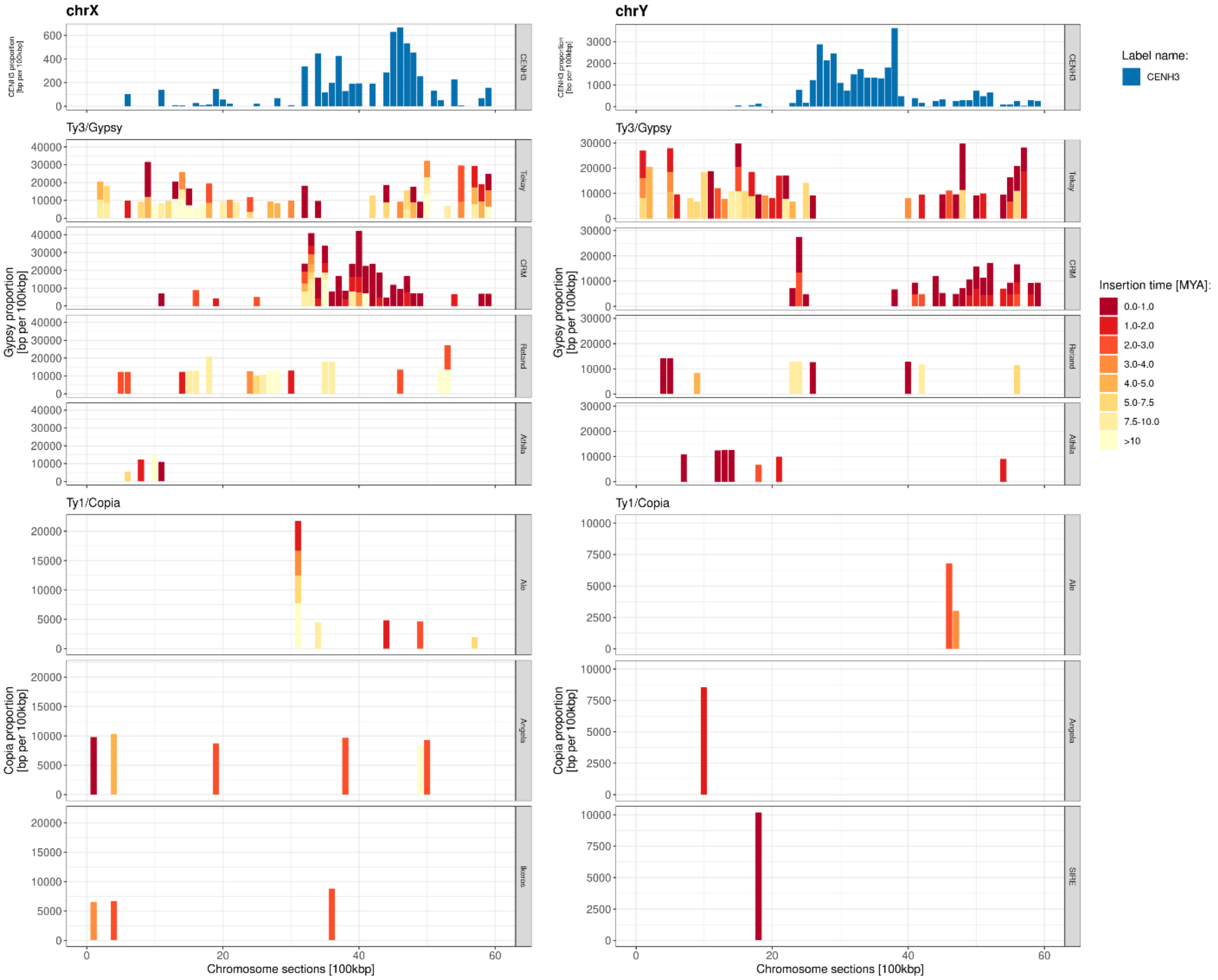

Fig. S19 Insertion times of autonomous (red), dominant non-autonomous (green), and minor non-autonomous (blue) CRM retrotransposons across all chromosomes in the *Humulus lupulus* genome. Chromosomes 4 and X exhibit the oldest insertions of both autonomous and non-autonomous (dominant and minor) CRM groups.

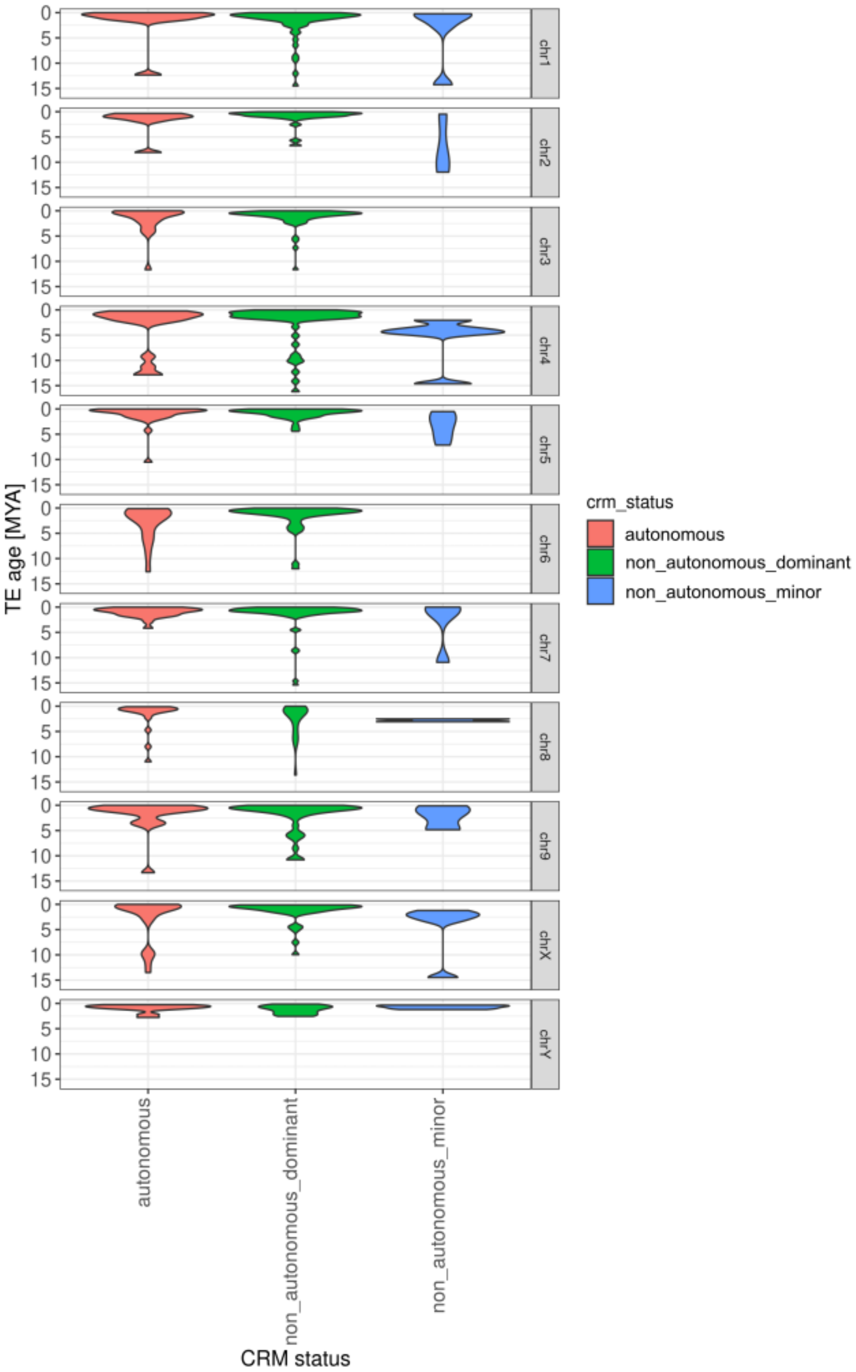

Fig. S20 Insertion time of the Ty3/*Gypsy* family of LTR retrotransposons in *Humulus lupulus* accessions. CRM insertions (green) are the most recent among all clades of the Ty3/*Gypsy* family. Most CRM insertions are up to 1 Mya.

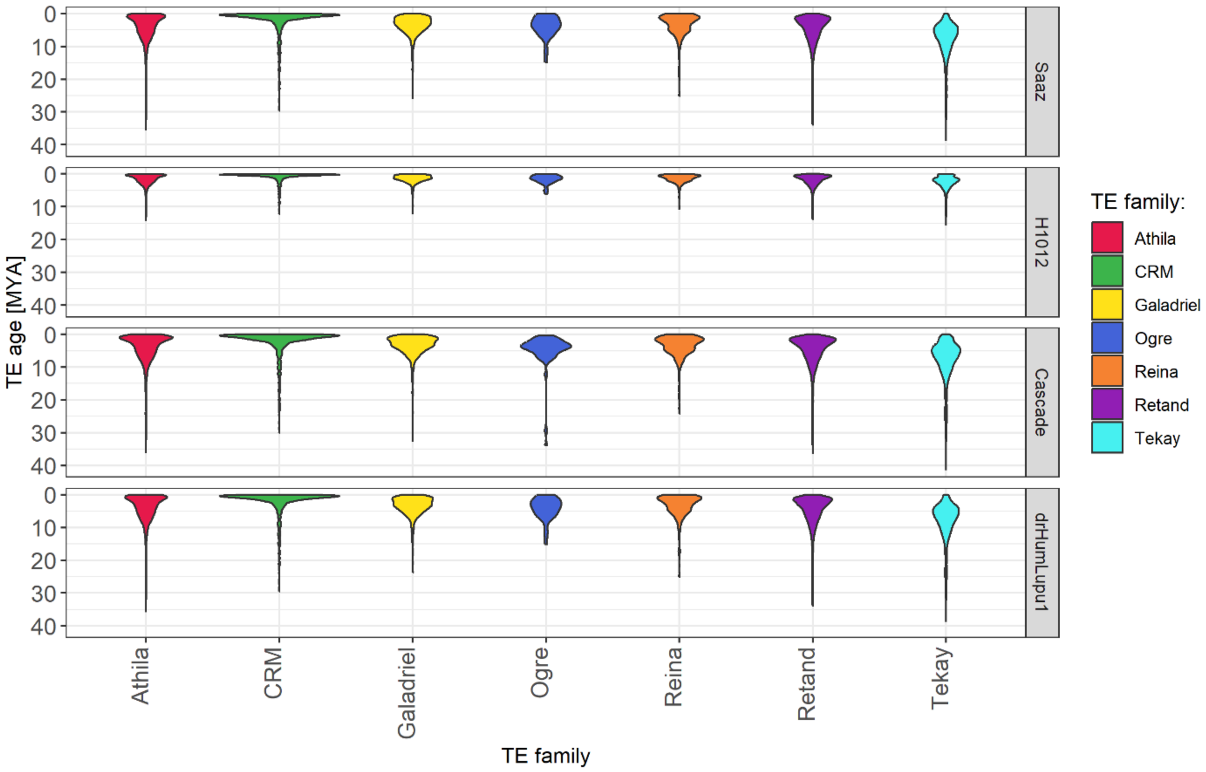

Fig. S21 The overall comparison and sequence length of centromeric Ty3/*Gypsy* CRM retrotransposons in *Humulus lupulus.* Autonomous CRMs (red), composed of one large open reading frame encoding all canonical proteins (reverse transcriptase, RNase H, integrase, chromodomain, and protease) are longer than both groups of non-autonomous CRMs (dominant and minor). Dominant non-autonomous CRMs (green) lack reverse transcriptase, RNase H, and integrase, while minor non-autonomous CRMs (blue) lack additional proteins.

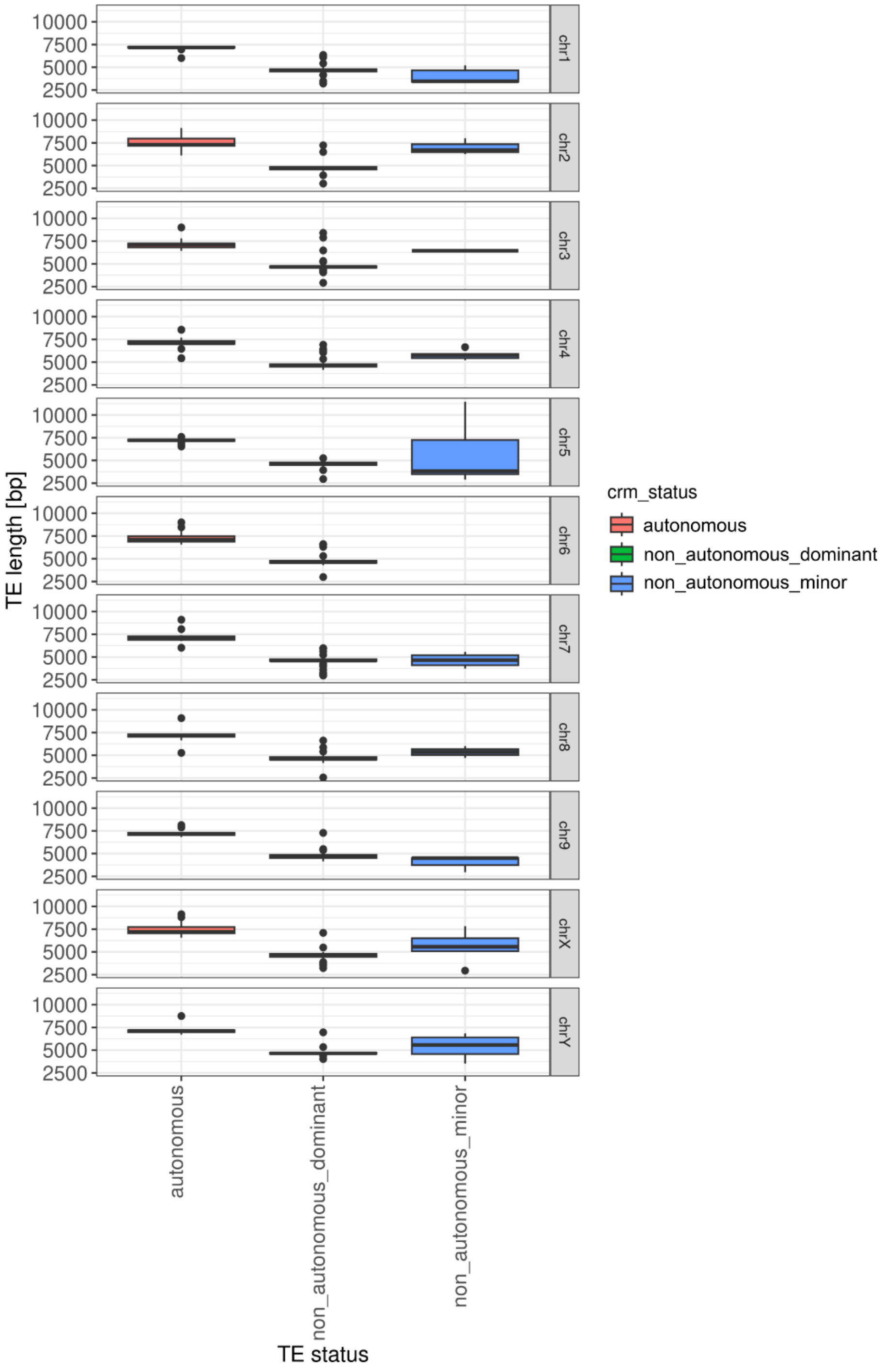

Fig. S22 (a) Phylogenetic analysis of autonomous and non-autonomous CRM retrotransposons in the *Humulus lupulus* centromere, correlated with insertion age (MYA). (b) The most abundant CRM lineages with recent insertions were found in non-autonomous elements (labeled by a gray oval). Insertion age was estimated as described in Material and Methods.

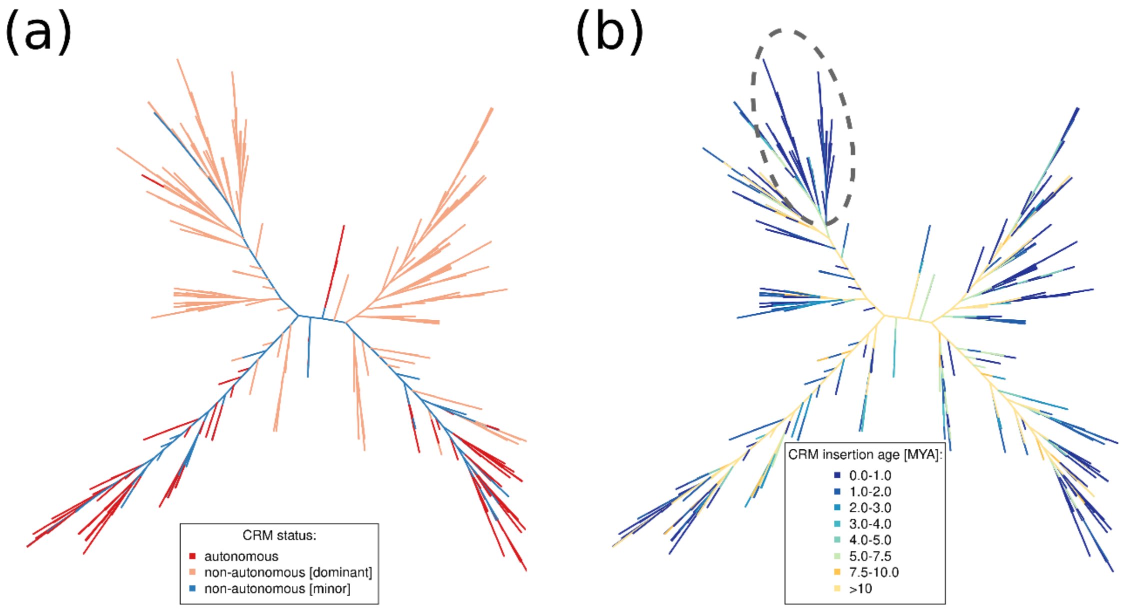

Fig. S23 Distribution of the centromeric repeat SaazCEN within autonomous and non-autonomous CRM retrotransposons in the *Humulus lupulus* centromere. The majority of CRMs (96.4%) contain the SaazCEN repeat (purple), while only a small proportion of CRMs (3.6%; orange) lack this repeat.

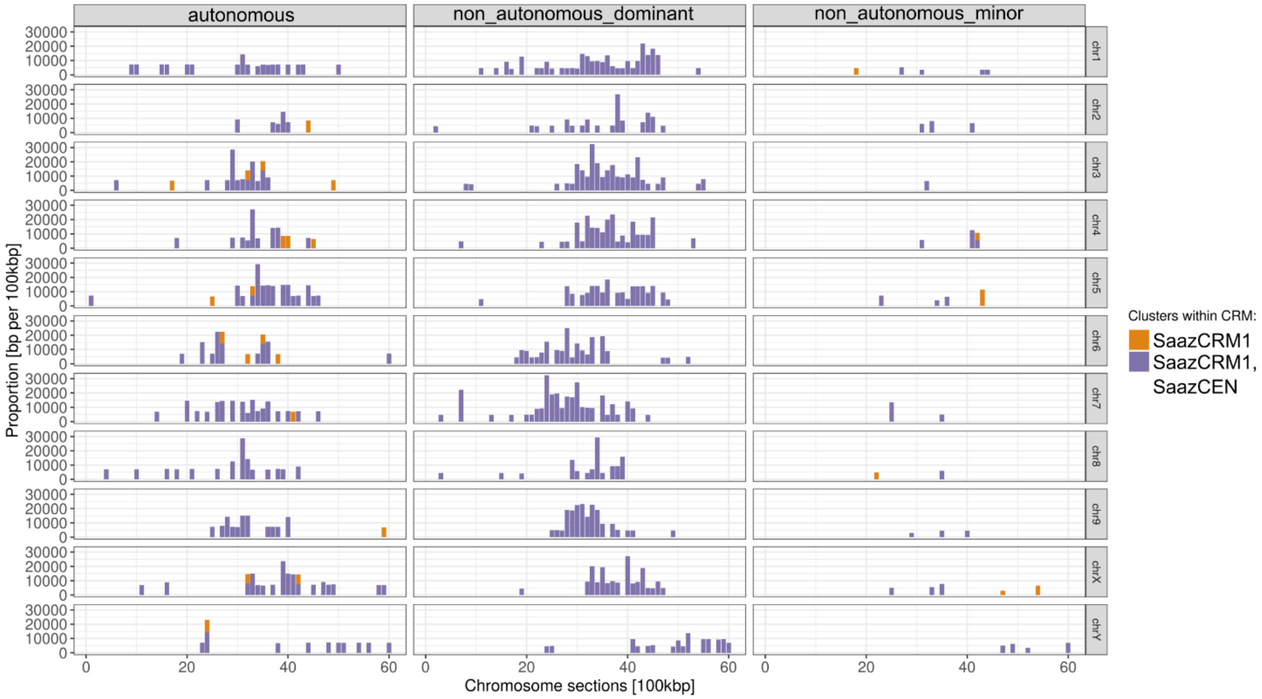

Fig. S24 Localization of HICENH3 summits within CRM retrotransposons in the *Humulus lupulus* centromere. HICENH3 binding is predominantly enriched within the spacer regions and long terminal repeats (LTRs) of CRM retrotransposons, whereas other domains exhibit low or no HICENH3 binding.

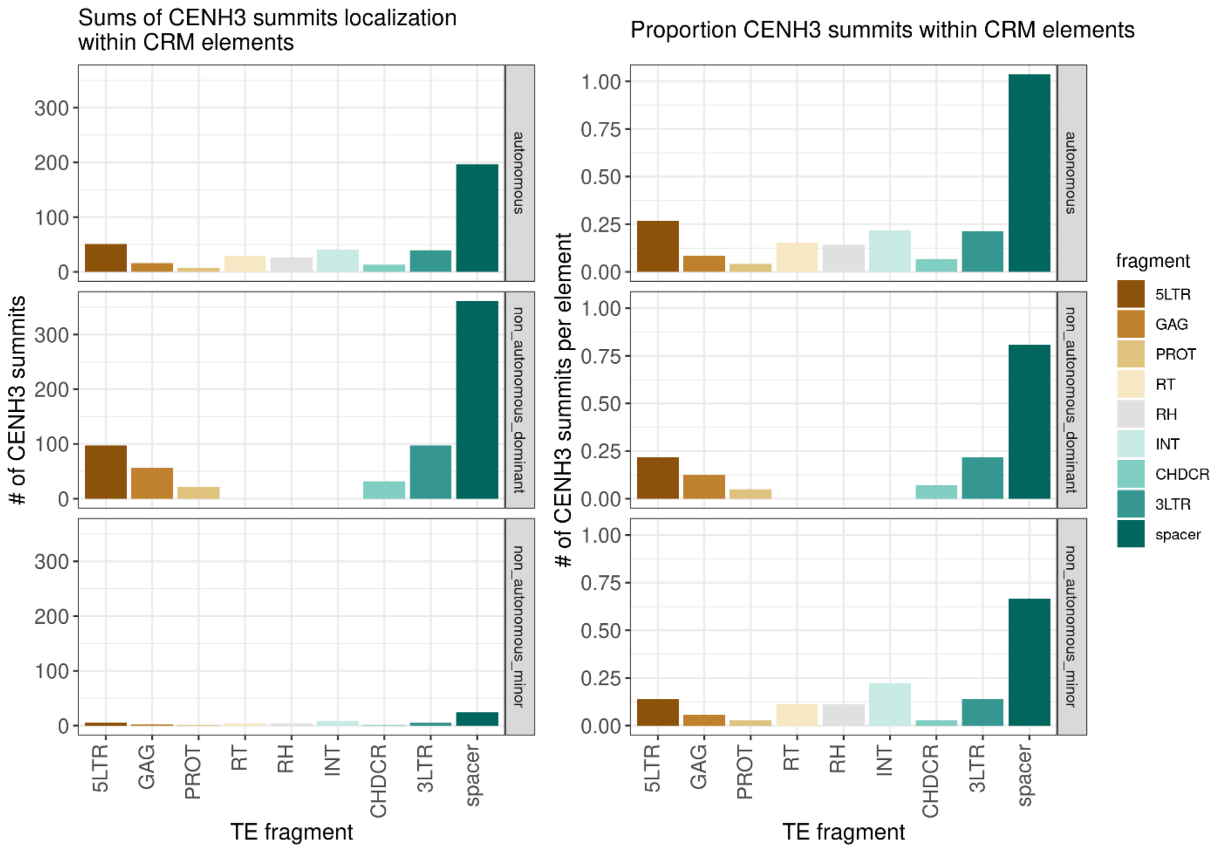

Fig. S25 Localization of satellite repeats on chromosomes 2 and 8 in *Humulus lupulus*. Chromosome 2 is characterized by the presence of HuluTR120 (red), 5S rDNA (green), and centromeric Saaz293 (cyan) repeats. Chromosome 8 is distinguished by the localization of the HSR1 satellite (magenta) and 45S rDNA (yellow). A dashed line indicates the position of a second 45S rDNA site on chromosome 8. Mitotic chromosomes were counterstained with DAPI. Scale bar = 10 μm.

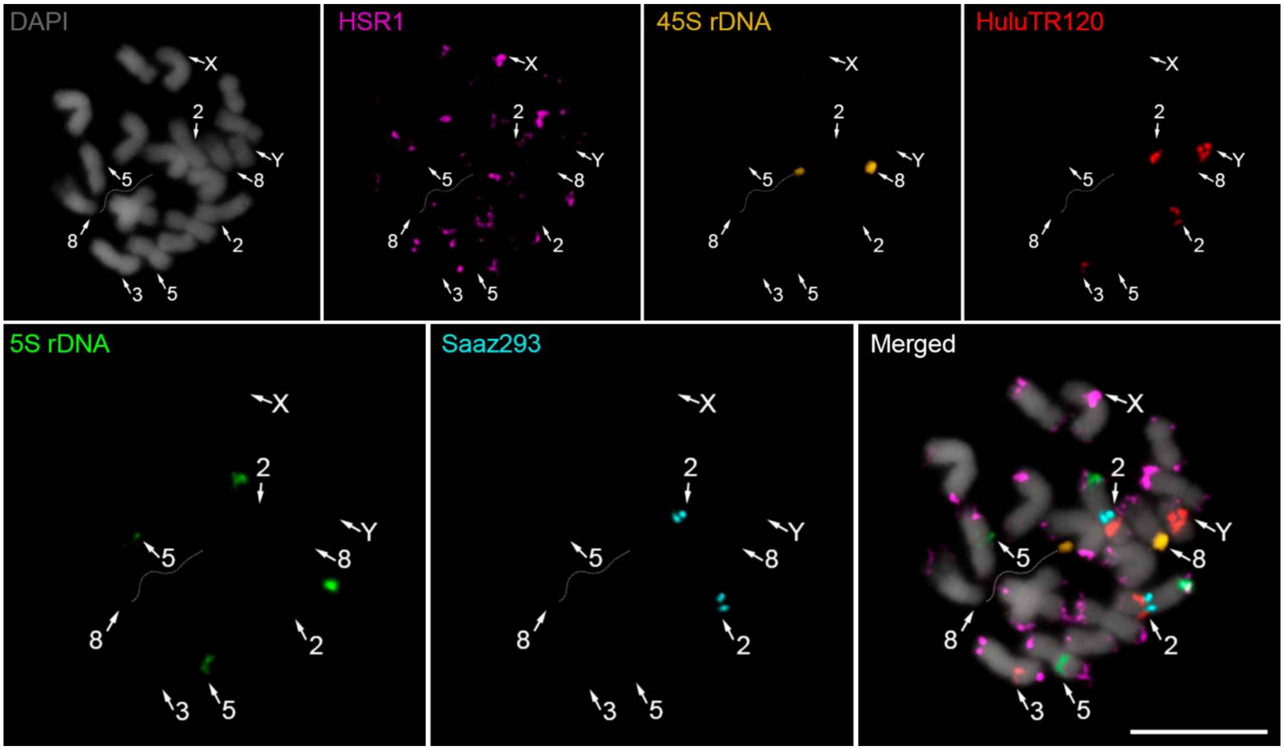

Fig. S26 Positioning of chromosome 2 within the interphase nucleus of male and female *Humulus lupulus*. (a) Two distinct loci of the centromeric satellite Saaz293 in the diploid (2n = 20) male nucleus, and (b) in the female nucleus. Arrows indicate the Saaz293 signal on chromosome 2 (green). The number of HuluTR120 signals (magenta) corresponds to the number of signals observed on metaphase chromosomes in diploid cells, specifically on chromosomes 2, 3, and Y (see Fig. 1d). Notably, both satellite clusters are localized at the nuclear periphery. Nuclei were counterstained with DAPI. Scale bar = 10 μm.

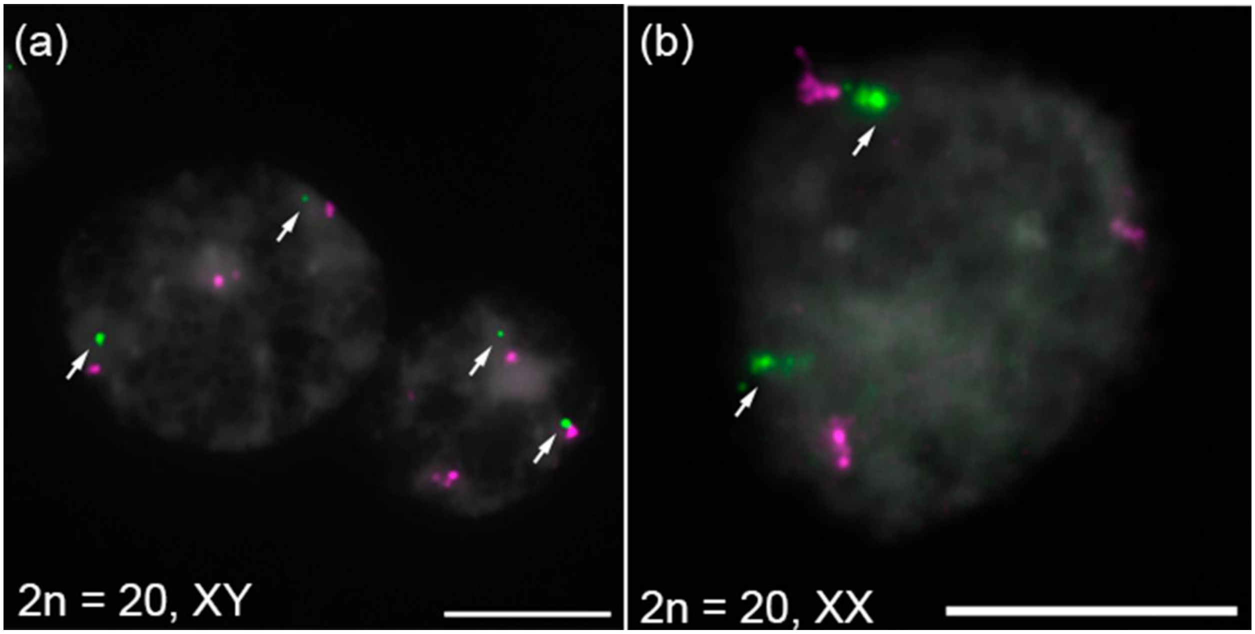

Fig. S27 Meiotic abnormalities observed during male meiosis. (a) A bivalent excluded from the metaphase plate (arrow); (b) unbalance segregation of chromosomes to opposite poles; (c) lagging chromosomes (arrow) during anaphase A; (d) lagging chromosome during anaphase B (arrow); (e) Unequal segregation of chromosomes during anaphase I in dwarf hop. Arrow indicates single signal of Saaz293 (green). (f) Unequal segregation of chromosomes during anaphase I in wild hop II. Arrow indicates single signal of Saaz293 (green). Chromosomes were counterstained with DAPI. Scale bar = 10 μm.

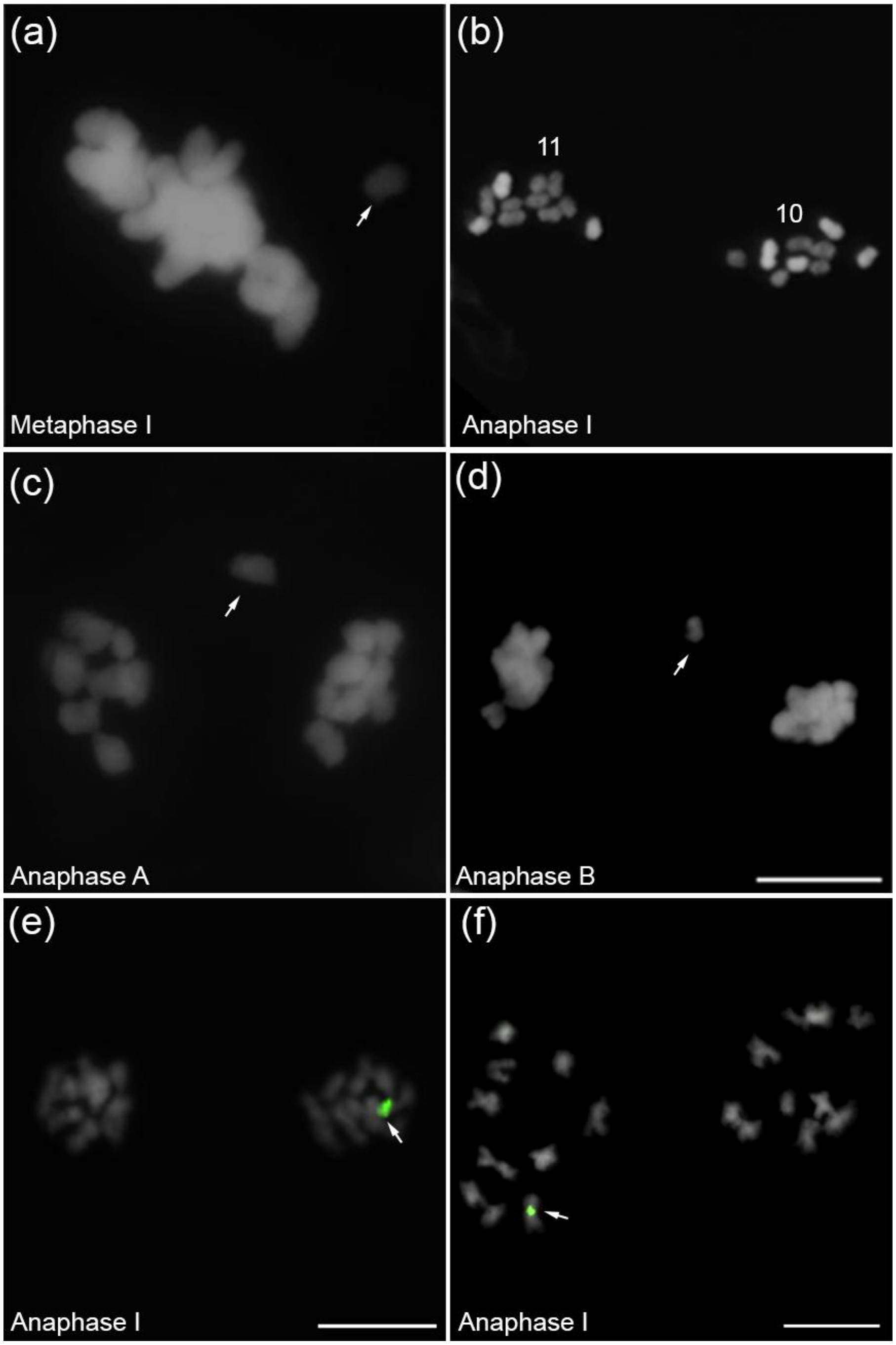

Fig. S28 Schematic model of chromosome 2 aberrant segregation in *Humulus lupulus*. (a) Chromosome mis-segregation due to lagging chromosome 2 (A2, pink), and (b) nondisjunction result in the non-Mendelian segregation patterns.

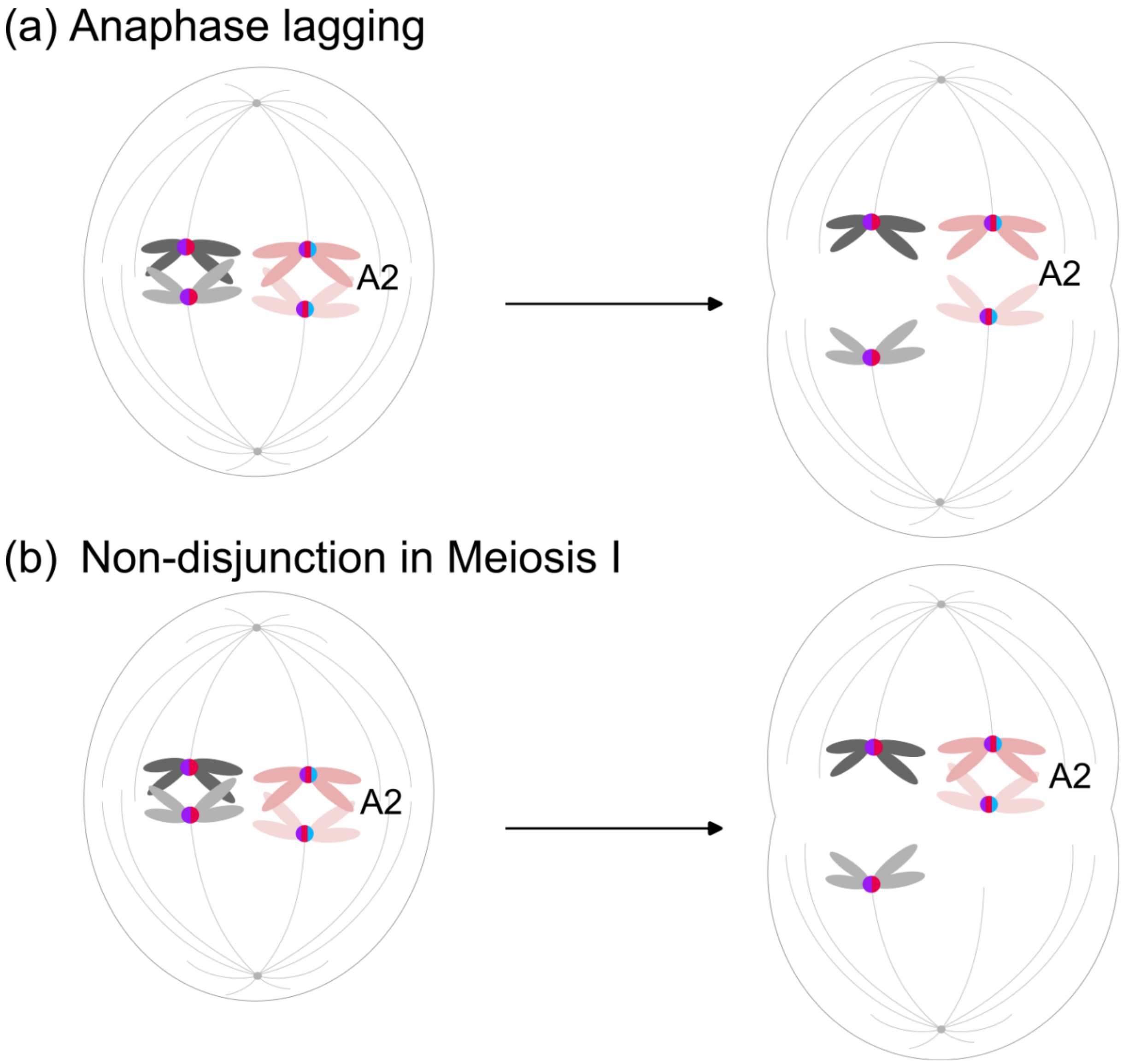

Table S1 List of *Humulus lupulus* plants used in this study.

**Hop cultivars**	**Chromosome number**	**Collected material**
Saaz hop - Osvald's clone 72	2n = 20, XX	Leaves
Liběšice male (Lib male)	2n = 20, XY	Leaves, panicles
15246	2n = 20, XY	Leaves
15249	2n = 20, XY	Leaves
15276	2n = 20, XY	Leaves
F_1 progeny (Osvald's clone 72 x Liběšice male)	2n = 20, XY	Panicles
Wild hop – Brno, Horní Heršpice	2n = 20, XY	Panicles
Dwarf hop	2n = 20, XY	Panicles
Wild hop II. – Brno, Jundrov	2n = 20, XY	Panicles

Table S2 List of primers used for PCR and preparation of FISH probes specific to *Humulus lupulus*

Primer name	Forward primer (5´-3´)	Reverse primer (5´- 3´)	GenBank Accession	References
HlCENH3	CCTCCTCTTCCTCCACTCCA	TTTCCCTCCAAGTCGACGTG		This study
Saaz293	ACAAATTCAATGGTGGCCGT	TCCCTGATATCTTTCTTTCTCCCT		This study
SaazCEN	GGGTTGTCTTTAAATTTCGGT	CAACCCTTTGTCTTCCTAATGA		This study
SaazCRM1	GGGCATGTGAGAGAAAGCATG	TACAACAAAGCCTAGGAAAACAAGC		This study
Saaz85	CCTTGTTCGGGATTTATTGAATCA	GTGGGTTAAACGAGTGAAAAAGA		This study
Saaz40	GGTCCGAGGTAGTGAGTTGTG	TCAAAATTTGTGCAGAAATCGGT		This study
HuluTR120	AGTTCCTGGATATAACCAGGTC	GAAAACTTAGTTCGTGTTAACT	MN537570	(Easterling *et al.*, 2020)
HSR1	CCCTCTGGTGAATTGGAGAT	CCTTTCAGAAATCTTCGATTTCTCTA	GU831574	(Divashuk *et al.*, 2011)
5S rDNA	GTTTTCAGGGGTGCAACACG	CTTACGGCTCAAAAGTTTGT	MN537579	(Easterling *et al.*, 2020)
45S rDNA	TGCCCGTTGCTCTGATGATT	TCCACCAACTAAGAACGGCC	AF223066.1	

New Phytologist

Table S3 Enzyme mixture used for the digestion of young leaves of *Humulus lupulus*.

**Enzyme mixture**	
1% pectolyase	P3026, Sigma-Aldrich
0.75% cellulase R-10 Onozuka	C8001.0005, DuchefaBiochemie
0.75% cellulase	219466, Sigma-Aldrich
1% cytohelicase	C8274, Sigma-Aldrich

Table S4 Genomic fraction of repetitive DNA in the *Humulus lupulus* Saaz female and Lib male genome estimated from genomic abundance of reconstructed contigs using the RepeatExplorer2 pipeline.

**Repeats**	**Superfamily**	**Lineage**	**Clade**	**Genome proportion (%) in *H. lupulus***		
				**Female**	**Male**	**Average**
LTR retrotransposons	Ty1/Copia	Ale		0.10	0.09	0.10
		Alesia		0.02	0.02	0.02
		Angela		7.98	7.90	7.94
		Bianca		0.01	0.00	0.01
		Ikeros		0.35	0.33	0.34
		Ivana		0.01	0.02	0.02
		SIRE		0.82	0.76	0.79
		TAR		2.44	2.35	2.40
		Tork		0.04	0.04	0.04
	Total Ty1/Copia			**11.77**	**11.51**	**11.64**
	Ty3/Gypsy	Chromovirus	CRM	0.20	0.21	0.21
			Galadriel	0.21	0.20	0.21
			Tekay	21.60	21.60	21.60
		Non-chromovirus	Athila	2.53	2.54	2.54
			Retand	15.13	15.26	15.20
	Total Ty3/Gypsy			**39.67**	**39.81**	**39.74**
Total LTR retrotransposons				**51.44**	**51.32**	**51.38**
DNA transposons		EnSpm_CACTA		2.49	2.34	2.42
		MuDR_Mutator		0.43	0.42	0.43
		PIF_Harbinger		0.04	0.05	0.05
		hAT		0.05	0.04	0.05
	Total DNA transposons			**3.01**	**2.85**	**2.93**
Tandem repeats	Satellite			0.35	0.32	0.34
	rDNA			0.52	0.60	0.56
Organelle				2.36	3.42	2.89
Unclassified				6.51	6.29	6.40
**Total**				**64.19**	**64.80**	**64.50**

Table S5 Tandem repeats in *Humulus lupulus* Saaz female and Lib male genome identified using the RepeatExplorer2 pipeline.

**Cluster**	**Supercluster**	**Monomer Length (bp)**	**HL MF ratio**	**Annotation**	**Accession**	**Reference**
HSR1 (Cl124)	60	383	0.89	Satellite	GU831574	(Divashuk *et al.*, 2011)
HSR0 (Cl304)	211	178	1.23	Satellite	MH188533.1	(Easterling *et al.*, 2018)
Saaz293 (Cl293)	200	323	1.32	Satellite		This study

Table S6 Number and frequency of meiotic abnormalities in three male accessions of *Humulus lupulus*.

**Meiotic abnormalities**	**Lib male**	**F1 progeny (Osvald's clone 72 x Lib male)**	**Wild hop**
**Regular tetrad stage**	1260 (87.50%)	1530 (87.28%)	1852 (89.43%)
**Unreduced cells**	133 (9.24%)	94 (5.36%)	129 (6.23%)
**Inviable microspore**	36 (2.50%)	122 (6.96%)	72 (3.48%)
**Micronuclei**	11 (0.76%)	7 (0.40%)	18 (0.87%)
Total	**1440**	**1753**	**2071**

Table S7 Number and frequency of viable and Inviable pollen grain in three male accessions of *Humulus lupulus*.

**Pollen viability**	**Lib male**	**F1 progeny (Osvald's clone 72 x Lib male)**	**Wild hop**
**Viable**	4909 (96.05%)	3994 (99.45%)	4413 (99.9%)
**Inviable**	202 (3.95%)	22 (0.55%)	4 (0.09%)
Total	**5111**	**4016**	**4417**

Methods S1 Extraction of DNA, genome sequencing and Characterization of repetitive DNA Genomic DNA from young leaves of male and female *H. lupulus* was isolated using the NucleoSpin Plant II (740770-50; Macherey-Nagel GmbH and Co. KG.), according to the manufacturer's instructions. The library was prepared using the NEBNext® Ultra™ II DNA Library Prep Kit and was sequenced on Illumina MiSeq sequencer generating 300 bp paired-end-reads at the Centre of Plant Structural and Functional Genomics in Olomouc, Czech Republic. Sequencing reads were checked for quality using the FastQC tool (available at http://www.bioinformatics.babraham.ac.uk/projects/fastqc). Reads were pre-processed based on quality (Q30) with subsequent adaptor trimming, filtering out short or unpaired sequences and all reads were trimmed to a uniform length of 200 bp using Trimmomatic 0.32 (Bolger *et al.*, 2014). The reads were randomly subsampled to reflect the *H. lupulus* genome, giving 710,500 reads for male and female, which is equivalent to ~ 0.1 x coverage of the genome size. These datasets were subjected to RepeatExplorer2 (Novák *et al.*, 2013, 2020) and TAREAN (Tandem repeat analyser) tools (Novák *et al.*, 2017) to identify repetitive DNA composition.

Notes S1 Repeatome analysis of Saaz female and Lib male of *Humulus lupulus*. A detailed comparison of transposable elements (TEs) and satellites reveals only minor differences between male and female accessions (Table S4). The overall repetitive fraction of *H. lupulus* genome comprising 64.50% DNA repeats (Table S4). The predominant repeat categories are Long Terminal Repeat (LTR) retrotransposons, specifically two major lineages of the Ty3/*Gypsy* superfamily (39.74%) and nine lineages of Ty1/*Copia* superfamily (11.64%), which together represent 51.38% of the genome. Within the Ty3/*Gypsy* superfamily, the chromovirus lineage is the most abundant, contributing 22.02% of the genome, including the CRM (0.21%), Galadriel (0.21%), and Tekay (21.60%). Retand and Athila from non-chromovirus lineage of Ty3/*Gypsy* superfamily account for 15.20% and 2.54%, respectively. Among the Ty1/*Copia* superfamily, the Angela lineage is the dominant contributor, making up 7.94% of the genome. Interestingly, DNA transposons form a minor part of the *H. lupulus* genome (2.93%). Other less abundant repeats include rDNA units (0.56%) and unclassified repeats (6.40%). Satellite DNA contributes 0.34% of the *H. lupulus* genome, and we have identified three specific high-order satellites. Of these, satellites cl124 and cl304 match the previously described subtelomeric repeats HSR1 (Divashuk *et al.*, 2011) and HSR0 (Easterling *et al.*, 2018). Additionally, a novel satellite Saaz293 was identified, with an approximate monomer unit length of 323 bp (Table S5).

References

Bolger AM, Lohse M, Usadel B. **2014**. Trimmomatic: A flexible trimmer for Illumina sequence data. *Bioinformatics* **30**: 2114–2120.

Divashuk MG, Alexandrov OS, Kroupin PY, Karlov GI. **2011**. Molecular cytogenetic mapping of Humulus lupulus sex chromosomes. *Cytogenetic and Genome Research* **134**: 213–219.

Easterling KA, Pitra NJ, Jones RJ, Lopes LG, Aquino JR, Zhang D, Matthews PD, Bass HW. **2018**. 3D molecular cytology of hop (humulus lupulus) meiotic chromosomes reveals non-disomic pairing and segregation, aneuploidy, and genomic structural variation. *Frontiers in Plant Science* **871**.

Easterling KA, Pitra NJ, Morcol TB, Aquino JR, Lopes LG, Bussey KC, Matthews PD, Bass HW. **2020**. Identification of tandem repeat families from long-read sequences of Humulus lupulus. *PLoS ONE* **15**.

Novák P, Neumann P, Macas J. **2020**. Global analysis of repetitive DNA from unassembled sequence reads using RepeatExplorer2. *Nature Protocols* **15**: 3745–3776.

Novák P, Neumann P, Pech J, Steinhaisl J, MacAs J. **2013**. RepeatExplorer: A Galaxy-based web server for genome-wide characterization of eukaryotic repetitive elements from next-generation sequence reads. *Bioinformatics* **29**: 792–793.

Novák P, Robledillo LÁ, Koblížková A, Vrbová I, Neumann P, Macas J. **2017**. TAREAN: A computational tool for identification and characterization of satellite DNA from unassembled short reads. *Nucleic Acids Research* **45**.
